# A spatial measure-valued model for radiation-induced DNA damage kinetics and repair under protracted irradiation condition

**DOI:** 10.1007/s00285-024-02046-3

**Published:** 2024-01-29

**Authors:** Francesco G. Cordoni

**Affiliations:** University of Trento: Universita degli Studi di Trento, Trento, TN Italy

**Keywords:** 60Gxx, 60G55, 92Bxx

## Abstract

In the present work, we develop a general spatial stochastic model to describe the formation and repair of radiation-induced DNA damage. The model is described mathematically as a measure-valued particle-based stochastic system and extends in several directions the model developed in Cordoni et al. (Phys Rev E 103:012412, 2021; Int J Radiat Biol 1–16, 2022a; Radiat Res 197:218–232, 2022b). In this new spatial formulation, radiation-induced DNA damage in the cell nucleus can undergo different pathways to either repair or lead to cell inactivation. The main novelty of the work is to rigorously define a spatial model that considers the pairwise interaction of lesions and continuous protracted irradiation. The former is relevant from a biological point of view as clustered lesions are less likely to be repaired, leading to cell inactivation. The latter instead describes the effects of a continuous radiation field on biological tissue. We prove the existence and uniqueness of a solution to the above stochastic systems, characterizing its probabilistic properties. We further couple the model describing the biological system to a set of reaction–diffusion equations with random discontinuity that model the chemical environment. At last, we study the large system limit of the process. The developed model can be applied to different contexts, with radiotherapy and space radioprotection being the most relevant. Further, the biochemical system derived can play a crucial role in understanding an extremely promising novel radiotherapy treatment modality, named in the community *FLASH radiotherapy*, whose mechanism is today largely unknown.

## Introduction

Radiotherapy is, today, a widely used treatment against cancer (Thariat et al. 2013). Conventional radiotherapy is based on X-rays, i.e. photons, but in the last decades constantly increasing attention has been devoted to advanced radiotherapy treatment with ions (Durante and Paganetti 2016). Ion beams have many essential features making them preferable compared to photons, related mostly to the extremely localized energy released in tissues which can lead to a superior biological effect than X-rays. The effect of radiation on biological tissue has been studied by the community over the last decades, and DNA is believed to be the most sensitive target to radiation so DNA damage is the most relevant biological vehicle that leads to cell killing induced by radiation (Durante and Loeffler 2010). Despite the potential superiority of hadrons in theory, additional research is crucial to incorporate this treatment modality into clinical practice fully. One of the primary obstacles to the widespread use of hadrons is in fact accurately estimating the biological effect caused by radiation, a crucial aspect to account for in order to prescribe the best possible treatment. Mathematical models have thus been developed over the years to understand and accurately predict the biological effect of ions on biological tissue (Bellinzona et al. 2021; Hawkins 1994; Hawkins and Inaniwa 2013; Kellerer and Rossi 1974; Herr et al. 2015; Pfuhl et al. 2020; Cordoni et al. 2021), focusing on the DNA damage Double Strand Breaks (DSB). Such mathematical approaches focus on developing models that describe the formation, evolution, and interaction of DSB, with the final goal of predicting the probability that a certain cell survives radiation.

To date, very few models in the context of radiotherapy have a robust mathematical and probabilistic background even if the community widely acknowledges stochastic effects play a major role in the biological effect of radiation. In fact, despite the early development of stochastic models for the description of the kinetic repair of radiation-induced DNA damages (Sachs et al. 1990; Albright 1989), the radiobiological community soon drifted to developing deterministic models of damage repair assuming Poisson fluctuations of the number of damages around the average values (Bellinzona et al. 2021). This type of modelization is strictly linked to a linear-quadratic description of the relation between the logarithm of the cell-survival probability and the absorbed dose, a physical quantity that describes the energy deposited by the particles over the mass of the biological tissue traversed by the particles. Although such models provide a fast way to assess the cell survival fraction, which is a key aspect for the use of such models in clinical applications in which the run time of a model is extremely relevant, in recent years, the need has begun to be felt for more robust modeling from a purely probabilistic point of view. From a mathematical point of view, the Generalized Stochastic Microdosimetric Model (GSM$^2$) recently introduced in Cordoni et al. (2021, 2022a, 2022b); Missiaggia et al. (2024), appears to be a general mathematical model, that includes several relevant stochastic effects emerging in the creation, repair, and kinetics of radiation-induced DNA-damages (Cordoni et al. 2022a). GSM$^2$ considers two types of DNA lesions $\textrm{X}$ and $\textrm{Y}$, representing respectively lesions that can be repaired and lesions that lead to cell inactivation. In the current context, the specific exact meaning of sub-lessons is left unspecified. This is because there are mainly two different ways that cells can be affected by radiation. One is the creation of DNA *Double-Strand Breaks* (DSB) from two *Single-Strand Breaks* (SSB), and the other is the formation of chromosome abnormalities from pairs of chromosome breaks (Kellerer and Rossi 1974). Both of these mechanisms are important in understanding how cells respond to radiation and can be described by the model developed in this work.

The present paper aims at extending GSM$^2$ to include a spatial description allowing for reaction rates that depend on the spatial position, lesion distance, and density. In fact, a true spatial distribution of DNA damage inside the cell nucleus is today almost completely missing in existing models. At the same time, it is widely known in the community that the spatial distribution of DNA damages strongly affects the probability that a cell repairs the induced damages, so spatial stochasticity plays a major role in the modelization of the repair of radiation-induced DNA damages. We will thus model the spatial distribution of DNA damages as a general measure-valued stochastic particle-based system, characterizing existence and uniqueness as well as some relevant martingale properties that, as standard, will play a crucial role in the derivation of the large system limit. Stochastic particle-based systems have been long studied in the mathematical community (Bansaye and Méléard 2015; Popovic et al. 2011; Pfaffelhuber and Popovic 2015). Recently, a lot of attention has been devoted to studying the spatial non-local stochastic particle-based system (Bansaye and Tran 2010; Fontbona and Méléard 2015; Champagnat and Méléard 2007; Ayala et al. 2022), where a measure-valued stochastic process describes the population. The population can interact according to a specific rate leading to either the creation or removal of individuals. Mathematically, these systems are described by Stochastic Differential Equations (SDE) driven by Lévy-type noises that besides a diffusive component include jump operators in the form of Poisson random measure, that account for the creation and removal of individuals from the population (Bansaye and Méléard 2015). Most results focus on birth-and-death spatial processes, meaning that at each time, at most, a single individual can be born or die. In this setting, pairwise interactions, involving either the creation or removal of more than one individual, are not allowed. Such interactions are relevant in many biological and chemical applications so a general mathematical theory that extends and generalizes the birth and death process is greatly desirable. Recently, few papers appeared that include pairwise reactions (Isaacson et al. 2022; Lim et al. 2020; Popovic and Véber 2023), but none of these deal with the existence and uniqueness or regularity of results for such systems, where instead the focus is mostly on the large-population limit. However, it is worth highlighting that the authors discuss useful techniques to show that the system considered is well-defined (Popovic and Véber 2023, Remark 2.7) or (Isaacson et al. 2022, Remark 5.2).

The developed model includes some key features that make the mathematical treatment of the spatial model non-trivial. First, given the application considered, where clusters of DNA lesions are more difficult to repair by the cell and have been recognized as one of the main factors that lead to cell inactivation in radiobiology (Kellerer and Rossi (1974)), we will include pairwise interaction and second-order rates, meaning that a couple of lesions can interact to create an unrepairable lesion that inactivates the cell. It is worth stressing that, already, many existing radiobiological models include parameters to account for the interaction of damages (Hawkins 1994; Sato and Furusawa 2012; Hawkins and Inaniwa 2013; Bellinzona et al. 2021; Cordoni et al. 2021, 2022a). Still, none have a true mathematical spatial formulation and often rely on fixed domains to limit pairwise interaction within a certain distance neglecting nonetheless any true spatiality inside a fixed domain. The latter approach can be restrictive and may lead to overfitting with the inclusion of unnecessary parameters. One of the proposed model’s main strengths is that it considers a true spatial distribution of lesions, allowing for true pairwise interaction that can depend on the distance between lesions, which is a novel and important aspect of the model. As mentioned, the existence and uniqueness results for second-order systems are rare and yet a general theory is missing, so the derived results represent a novelty both from a radiobiological as well as a mathematical perspective.

Another key aspect of the studied model is that we explicitly consider the case of protracted irradiation, that is we consider the situation in which a continuous radiation field induces a random number of lesions in the cell. Such a situation is non-trivial from a purely mathematical perspective as the generation of a random number of damages must be considered. Nonetheless, it is extremely relevant to include protracted irradiation in a biological model since it allows us to better estimate the kinetics repair of radiation-induced damage with benefits both in radioprotection and clinical application. Existing radiobiological models account to a certain extent for the protracted irradiation case (Inaniwa et al. 2013; Manganaro et al. 2017), and a similar rate has already been considered in Cordoni et al. (2021) in a non-spatial setting. In generalizing the setting to include a spatial description, we need to describe a spatial energy deposition pattern within the cell nucleus. We will build such a theory properly generalizing some existing approaches. Nonetheless, a robust theory to account for the spatial formation of radiation-induced DNA lesions is missing and future efforts will be made to derive such a theory. In fact, another relevant aspect of the studied model is the computation of the spatial distribution of radiation-induced DNA damages. Such a description is relevant both in the protracted case and in the instantaneous irradiation case as it describes the initial damage distribution. In particular, the initial damage distributions, $\nu ^\textrm{X}_0$, and $\nu ^\textrm{Y}_0$ can be computed using different methods. One possible approach is the use of Monte Carlo (MC) track structure codes (Nikjoo et al. 2006), to simulate the passage of charged particles in biological tissue and their energy release and to estimate the DNA damage distribution caused by radiation. MC track structure codes have been shown to be effective in accurately characterizing DNA damage formation (Goodhead 1994; Ottolenghi et al. 1995; Cucinotta et al. 2000; Chatzipapas et al. 2022; Kyriakou et al. 2022; Zhu et al. 2020; Thibaut et al. 2023), however, once the initial damage distribution is computed, in order to assess the cell survival probability, these models typically neglect the spatial distribution of damages and focus on average values described by *Ordinary Differential Equations* (ODE). The model developed in this research is unique in that it is able to fully exploit the accuracy of the spatial distribution of damages as predicted by MC track-structure codes. Further, since MC track structure codes simulate all the energy released by a particle along its path, which is referred to in the community as track, as well as all secondary energy releases associated with the original particle, the computational time is extremely demanding. To shorten the computational time, a threshold on energy release can be applied. so that all events that release lower than a certain energy are neglected and incorporated into the deposition that has originated it. Such an approach is called *condensed history* MC (Agostinelli et al. 2003), and it provides accurate results of energy deposition at a lower computational time compared to MC track structure codes.

An alternative approach to MC track-structure codes would be to develop an analytical model for DNA damage formation and distribution. Such a model would be less accurate, but less computationally expensive. Currently, the *Local Effect Model* (LEM) (Friedrich et al. 2012), and the *Microdosimetric Kinetic Model* (MKM) (Hawkins 1994; Kase et al. 2007), which are the only models used in *Treatment Planning Systems* (TPS), take into account the spatial distribution of the absorbed dose without MC codes. These models are based on the *Amorphous Track* (AT) model (Kase et al. 2007), which parametrizes the dose distribution around a track of a particle. However, to eventually assess the cell-survival probability, both models make extensive use of fixed domain so that a true spatial distribution of damages is again neglected. It is worth further stressing that, although the AT model can be used to compute the imparted dose in a fast fashion, it is based on several assumptions, such as the so-called track-segment condition (assumes tracks do not lose energy when traversing the cell nucleus) and uniform radiation fields (cylindrical geometry is often assumed for the cell nucleus and tracks are perpendicular to the cell nucleus). Being the former approach based on track-structure codes, which are beyond the scope of the present work, we will focus on the latter one. Nonetheless, future research will focus on developing a comprehensive analytical model for DNA damage formation that accurately describes energy and spatial stochasticity.

The developed spatial DNA-damage model is expected to play a relevant role in the modelization of a novel radiotherapeutic technique, named in the community FLASH radiotherapy (Favaudon et al. 2014). Up to 2014, most of the mechanisms happening in the interaction of radiation with biological tissue were believed to be known, and therefore in the last two decades models focused on specific applied aspects and general and robust mathematical theories were strongly believed to be unnecessary given the overall understanding of the problem at hand. Starting in 2014 a series of ground-breaking papers (Favaudon et al. 2014; Montay-Gruel et al. 2017; Vozenin et al. 2019) finally showed that an increase in the rate of delivery of ions at Ultra-High Dose Rates (UHDR), namely a high amount of energy released in a small fraction of time, spares healthy tissue and yet maintains the same effect on the tumor. This effect, which was completely unexpected, represents the final goal of any radiotherapeutic treatment (Esplen et al. 2020; Griffin et al. 2020). All available models brutally failed to predict such peculiar effects and hundreds of publications have appeared recently trying to understand the mechanism at the very core of the FLASH effect (Labarbe et al. 2020; Abolfath et al. 2020a, b; Liew et al. 2021; Petersson et al. 2020; Battestini et al. 2023); many physical explanations and mathematical models have been proposed in the last five years, but up to date, no model is believed to be capable neither of predicting nor understanding the origin of the FLASH effect (Weber et al. 2022). Two facts are today believed to be at the very core of the FLASH effect: (i) this effect has its origin in a spatial interaction of ions, that involves besides the physics of ions and biology, also chemistry, and (ii) nonlocal effects of ions while traversing different cells and how their spatial interaction affects the overall chemical and biological environment. The model developed in the present research could, when coupled with an adequate description of the chemical environment affected by radiation, help unravel the mechanism behind the FLASH effect. In order to do that, we couple the spatial model with a reaction–diffusion equation that describes the evolution of the chemical system. In the following treatment, we will not specify a particular chemical description. This is because there are several possible choices, and the choice depends on the specific application. The chemical stage can be broadly divided into two stages: (i) the heterogenous chemical stage and (ii) the homogeneous chemical stage. The former is characterized by a heterogeneous spatial distribution and occurs immediately after particles hit the cell nucleus, i.e. between $10^{-12}$ seconds and $10^{-6}$ seconds. The latter is characterized by a homogeneous distribution and occurs after the heterogeneous stage, i.e. between $10^{-6}$ seconds to $10^0$ seconds. Mathematically, this means that while the homogeneous stage can be described by ODEs that characterize the time evolution of the concentration of chemicals within the domain (Labarbe et al. 2020; Abolfath et al. 2020a), the heterogeneous stage requires advanced mathematical tools since the system is highly nonlinear and the reactions occur locally. Therefore, most of the literature is devoted to the development of simulation codes (Clifford et al. 1986; Pimblott et al. 1991; Boscolo et al. 2020; Ramos-Méndez et al. 2020). To date, there is no general mathematical formulation via local reaction–diffusion PDEs that exists in literature, even though it could provide an accurate representation of the system and fast computational time. Also, general results of well-posedness that cover relevant non-local chemical systems are not available in the literature due to the highly complex mathematical formulation needed. For these reasons, a deep mathematical study of such a system is left for future research. Relevant systems to study could include a spatial non-local version of the homogeneous chemical systems presented in Labarbe et al. (2020); Abolfath et al. (2020a) or an analytical formulation in terms of highly dimensional non-local reaction–diffusion PDEs of Boscolo et al. (2020); Ramos-Méndez et al. (2020). In this paper, we will instead limit ourselves to considering a general reaction–diffusion PDE with coefficients satisfying rather general assumptions that could in principle include several relevant examples. In particular, almost any chemical description includes bimolecular reactions, meaning that the resulting PDE has quadratic terms. A significant effort has been made in the literature to study reaction–diffusion PDEs under the most general assumptions on the coefficients in order to include as many examples as possible (Pierre 2010). In this direction, a mass control assumption has been typically seen as a general condition that allows obtaining the existence and uniqueness of the equations in many cases dropping the standard global Lipschitz condition. For the sake of simplicity, we will consider the system introduced in Fellner et al. (2020), which allows for quadratic growth of the coefficients, adding random discontinuity due to the effect of radiation. Therefore, we prove the existence and uniqueness of a stochastic particle-based system coupled with a reaction–diffusion system with random jumps. We will show later that this system can be generalized to other relevant systems. A future effort will be devoted to the study of general local reaction–diffusion systems similar to the ones studied in Isaacson et al. (2022).

At last, we will also characterize the large-system behavior. Such a limiting system can be obtained with standard arguments proving the tightness of the measure and identifying the limiting process, which can be proved to admit a unique solution. Although the techniques are standard, the result is new in the literature of radiation biology since no stochastic system allowing for pairwise interaction and creation of random numbers of particles has ever been studied before. In fact, the resulting governing equation can be useful to study the behavior of the system at high doses, where the number of damages within the cell increases arbitrarily. It is worth stressing that, the high-dose case is recognized to be non-trivial and most of the existing models fail to predict the behavior of the system at high doses. For this reason, often, correction terms are included in the model to better match experimental data (Bellinzona et al. 2021).

Since the early study of radiation-induced biological damage, it has been understood that the micrometer scale was extremely relevant to both asses and understand the main mechanism at the origin of DNA damage formation and repair. Among the first mathematical models developed to link the physics of radiation to the biological effect, there is the Theory of Dual Radiation Action (Kellerer and Rossi 1974), where it is conjectured that a site in the order of micrometers is the most relevant domain to be considered. Nonetheless, experiments immediately emerged suggesting that, besides a micron scale, a smaller scale in the order of nanometer should be considered (Goodhead 1982; Goodhead et al. 1978). Such scale could give a better resolution in accounting for track interaction and damage clustering, pathways that could be lost in a coarse averaging within fixed domains. This observation led to the development of the Generalized Theory of Dual Radiation Action (Kellerer and Rossi 1978), where a damage recombination pathway has been introduced based on the distance between lesions. Perhaps difficulties in the advanced formalism used and in the experimental benchmark have severely limited the usage of such a model. Consequently, the community leaned towards predominantly adopting models grounded in fixed domains, as they continued to yield results that reasonably matched biological experimental data. However, recent years have witnessed a significant boost in computational power, which has greatly promoted the utilization of advanced computational tools capable of accurately modeling intricate biological targets and simulating biological damage with micrometer-level precision. With this resurgence of interest, there arises a crucial need to formulate advanced mathematical models capable of not only capturing the temporal repair of damages but also elucidating how this repair process is influenced by spatial distribution. Such advancements hold the potential not only for enhanced predictions of specific biological outcomes but, perhaps even more importantly, for a deeper comprehension of the fundamental biological mechanisms underpinning these endpoints.

The main contributions of the present paper are:

(i) to provide a general mathematical description of a spatial model governing the formation and kinetics of radiation induced-damages;
(ii) to study the well-posedness of a measure-valued stochastic particle system with pairwise interaction and random creation of damages;
(iii) to propose a multi-scale model to couple biology and chemistry that could possibly describe the FLASH effect;
(iv) to study the large-system limit of the system with pairwise interaction and protracted irradiation.

## The microdosimetric master equation

The main goal of the present section is thus to introduce the classic setting for the GSM$^2$ (Cordoni et al. 2021, 2022b). GSM$^2$ models the time-evolution of the probability distribution of the number of sub-lethal and lethal lesions denoted by $\left( \textrm{X}(t),\textrm{Y}(t)\right)$, where $\textrm{X}$ and $\textrm{Y}$ are two $\mathbb {N}-$valued random variables counting the number of the lethal and sub-lethal lesion, respectively. In the following, we will consider a standard complete filtered probability space $\left( \Omega ,\mathcal {F},\left( \mathcal {F}_t\right) _{t \ge 0},\mathbb {P}\right)$ satisfying usual assumptions, namely right–continuity and saturation by $\mathbb {P}$–null sets.

We thus assume that a sub-lethal lesion $\textrm{X}$ can undergo three different pathways: (i) at rate $\textrm{r}$ a sub-lethal lesion is repaired, (ii) at rate $\textrm{a}$ a sub-lethal lesion is left unrepaired by the cell and thus it becomes a lethal lesion and (iii) at rate $\textrm{b}$ two sub-lethal lesion form a cluster that cannot be repaired by the cell and thus become a lethal lesion. Any lethal lesion leads to cell inactivation. These three pathways can be summarized as follows

1 $$\begin{aligned} \begin{aligned}&\textrm{X}\xrightarrow {\textrm{r}} \emptyset \,,\\&\textrm{X}\xrightarrow {\textrm{a}} \textrm{Y}\,,\\&\textrm{X}+ \textrm{X}\xrightarrow {\textrm{b}} \textrm{Y}\,.\\ \end{aligned} \end{aligned}$$

Denoting by

$$\begin{aligned} p(t,y,x) = \mathbb {P}\left( \left( \textrm{Y}(t),\textrm{X}(t)\right) = \left( y,x\right) \right) \,, \end{aligned}$$

the probability to have at time *t* exactly *x* sub-lethal lesion and *y* lethal lesions, following (Cordoni et al. 2021), we can obtain the *microdosimetric master equation* (MME)

2 $$\begin{aligned} {\left\{ \begin{array}{ll} \frac{\partial }{\partial t} p(t,y,x) = \left( E^{0,1} -1\right) \left[ x r p(t,y,x)\right] + \left( E^{-1,1} -1\right) \left[ x \textrm{a}p(t,y,x)\right] \\ \qquad \qquad \qquad \qquad + \left( E^{-1,2} -1\right) \left[ x(x-1) \textrm{b}p(t,y,x)\right] \,,\\ p(0,y,x) = p_0(y,x)\,, \end{array}\right. } \end{aligned}$$

where we have denoted the creation operator as

$$\begin{aligned} \left( E^{i,j}-1\right) \left[ f(y,x)\right] := f(y+i,x+j) - f(y,x)\,. \end{aligned}$$

In Cordoni et al. (2022b) a closed-form solution is derived for the survival probability as predicted by the MME (2), defined as the probability of having no lethal lesions $\textrm{Y}$. Further, GSM$^2$ is closely connected with one of the most used radiobiological models to predict the survival probability of cell nuclei when exposed to ionizing radiation, that is the Microdosiemtric Kinetic Model (MKM) (Hawkins 1994). The main equations of the MKM describe the time-evolution of the average value $\bar{y}$, resp. $\bar{x}$, of the number of lethal, resp. sub-lethal, lesions, and are given by

3 $$\begin{aligned} {\left\{ \begin{array}{ll} \frac{d}{dt} \bar{y}(t) = \textrm{a}\bar{x} + b \bar{x}^2\,,\\ \frac{d}{dt} \bar{x}(t) = - (\textrm{a}+\textrm{r}) \bar{x} - 2 \textrm{b}{\bar{x}^2}\,.\\ \end{array}\right. } \end{aligned}$$

The model further assumes that $\bar{y}$ is the average of a Poisson random variable so that by describing the average values we have complete knowledge of all the moments.

To obtain a suitable analytical solution to the Eq. (3), it is often assumed that $(\textrm{a}+ \textrm{r}) \bar{x}>> 2\textrm{b}{\bar{x}^2}$, so that above equation is reduced to

4 $$\begin{aligned} {\left\{ \begin{array}{ll} \frac{d}{dt} \bar{y}(t) = \textrm{a}\bar{x} + b \bar{x}^2\,,\\ \frac{d}{dt} \bar{x}(t) = - (\textrm{a}+\textrm{r}) \bar{x} \,.\\ \end{array}\right. } \end{aligned}$$

This highlights why in the high dose case the MKM must be corrected including additional terms. In fact, even if it is typically true that $(\textrm{a}+ \textrm{r})>> 2\textrm{b}$, at sufficiently high doses, the number of lesions $\bar{x}$ increases so that $(\textrm{a}+ \textrm{r}) \bar{x}$ does not dominate anymore $2\textrm{b}{\bar{x}^2}$ and therefore the omission of the term in Eq. (4) becomes non-negligible.

Further, it has been shown in Cordoni et al. (2021), that the average of the MME coincides with the MKM equations (3) under a suitable *mean-field assumption*, that is

$$\begin{aligned} \mathbb {E}\left[ \textrm{X}(t)(\textrm{X}(t)-1)\right] \approx \mathbb {E}\left[ \textrm{X}(t)\right] ^2\,, \end{aligned}$$

which in turn coincides exactly with the requirement that $\textrm{X}$ follows a Poisson distribution. It has thus been shown in Cordoni et al. (2022a) that the GMS$^2$ is able to give a more general description of many stochastic effects relevant to the formation and repair of radiation-induced DNA lesions that play a crucial role in estimating the surviving probability of a cell nucleus.

It can be further shown (Weinan et al. 2021; Bansaye and Méléard 2015), that Eq. (2) describes the time evolution for the probability density function associated with the following *stochastic differential equation* (SDE)

5 $$\begin{aligned} {\left\{ \begin{array}{ll} \textrm{Y}(t) = \textrm{Y}_0 + \int _0^t \int _{\mathbb {R}_+} f^{\textrm{Y}}(\textrm{X}(s^-),z) N^{\textrm{Y}}(ds,dz),\\ \textrm{X}(t) = \textrm{X}_0 - \int _0^t \int _{\mathbb {R}_+} f^{\textrm{X}}(\textrm{X}(s^-),z) N^{\textrm{X}}(ds,dz),\\ \end{array}\right. } \end{aligned}$$

with

6 $$\begin{aligned} f^{\textrm{Y}}(\textrm{X}(s^-),z)= & {} \mathbb {1}_{\left\{ z \le \textrm{a}\textrm{X}(s^-)\right\} } + \mathbb {1}_{\left\{ \textrm{a}\textrm{X}(s^-) \le z \le \textrm{a}\textrm{X}(s^-) + \textrm{b}{\textrm{X}(s^-)\left( \textrm{X}(s^-) -1\right) }\right\} }\nonumber \\ f^{\textrm{X}}(\textrm{X}(s^-),z)= & {} \mathbb {1}_{\left\{ z \le (\textrm{a}+\textrm{r}) \textrm{X}(s^-)\right\} } + 2 \mathbb {1}_{\left\{ (\textrm{a}+ \textrm{r}) \textrm{X}(s^-) \le z \le (\textrm{a}+ \textrm{r}) \textrm{X}(s^-) + \textrm{b}{\textrm{X}(s^-)\left( \textrm{X}(s^-) -1\right) }\right\} }.\nonumber \\ \end{aligned}$$

Above in Eq. (5), $N^Y(ds,dz)$ and $N^X(ds,dz)$ are two independent Poisson point measure with intensity $ds\,dz$ on $\mathbb {R}_+ \times \mathbb {R}_+$, see, e.g. Applebaum (2009). The main contribution of the present work will be to provide a spatial description of the SDE (5) so that $\textrm{X}$ and $\textrm{Y}$ are replaced by random measures.

### On the initial distribution

To later generalize the initial damage distribution, we introduce in the current section the distribution introduced in Cordoni et al. (2021, 2022a, 2022b). For a detailed treatment, we refer the interested reader to the mentioned papers or to Bellinzona et al. (2021).

Among the most powerful approaches to describe the formation of DNA lesions is using microdosimetry (Zaider et al. 1996). Microdosimetry is the branch of physics that investigates the energy deposition in domains comparable to cell nuclei, that is of the order of some microns. At that scale, energy deposition is purely stochastic, so the main objects used in microdosimetry are random variable and their corresponding distributions (Missiaggia et al. 2023, 2020). Over the years many models have been developed based on microdosimetric principles (Kellerer and Rossi 1974; Zaider et al. 1996), and both the MKM and GSM$^2$ assess the formation of DNA lesions using microdosimetry (Hawkins 1994; Bellinzona et al. 2021; Cordoni et al. 2021, 2022b).

The main microdosimetric quantity of interest from the point of view of radiobiological models is the *specific energy* *z* (Zaider et al. 1996). The *specific energy* *z* is the ratio between energy imparted by a finite number of energy depositions $\varepsilon$ over the mass *m* of the matter that has received the radiation, that is

$$\begin{aligned} z = \frac{\varepsilon }{m}\,. \end{aligned}$$

The stochastic nature of $\varepsilon$ implies that also *z* is inherently stochastic. The single–event distribution $f_{1}(z)$ of energy deposition on a domain (Zaider et al. 1996), is the probability density distribution describing the energy deposition due to a single event, typically a particle traversing the domain. Such distribution is associated with a random variable *Z* that describes the specific energy imparted on a certain domain of mass *m*. The average values of the random variable *Z*, referred to in the literature as *fluence-average specific energy*, that is the mean specific energy deposition, is typically denoted in literature as $z_F$. By additivity property, the specific energy distribution resulting from $\nu$ tracks can be computed convolving $\nu$ times the single event distribution (Zaider et al. (1996)). Therefore, the distribution $f_{\nu }$ of the imparted energy *z* is computed iteratively as

$$\begin{aligned} \begin{aligned} f_{2}(z)&:= \int _0^\infty f_{1}(\bar{z})f_{1}(z-\bar{z})d\bar{z}\,,\\&\dots \,,\\ f_{\nu }(z)&:= \int _0^\infty f_{1}(\bar{z})f_{\nu -1}(z-\bar{z})d\bar{z}\,.\\ \end{aligned} \end{aligned}$$

We denote by $p_e(\nu |D,z_F)$ a discrete probability density distribution denoting the probability of registering $\nu$ events. Typically such distribution is assumed to be dependent on the total dose absorbed by the mass and the fluence of the incident particles. The standard assumption is that, since events are in a microdosimetric framework assumed to be independent, the distribution $p_e$ is a Poisson distribution of average $\frac{D}{z_F}$ so that we have

$$\begin{aligned} p_e(\nu |D,z_F) := \frac{e^{- \frac{D}{z_F}}}{\nu !}\left( \frac{D}{z_F}\right) ^{\nu }\,. \end{aligned}$$

Therefore, microdosimetry postulates that the actual energy deposition on a certain domain can be obtained via the *multi-event specific energy distribution*

$$\begin{aligned} f(z|D) := \sum _{\nu = 0}^\infty \frac{e^{- \frac{D}{z_F}}}{\nu !}\left( \frac{D}{z_F}\right) ^{\nu } f_{\nu }(z)\,. \end{aligned}$$

At last, given a certain specific energy deposition *z* by $\nu$ events, the induced number of lethal and sub-lethal lesions is again a random variable, with a discrete probability density function denoted by *p*. In general the average number of lethal, resp. sub-lethal, lesions is assumed to be a function of *z*, namely $\kappa (z)$, resp. $\lambda (z)$. Again, by independence on the number of created lesions, such distribution is assumed to be a Poisson distribution. Overall, the probability of inducing *x* sub-lethal and *y* lethal lesions can be computed as (Cordoni et al. 2021),

7 $$\begin{aligned} p_0(x,y) = \sum _{\nu = 0}^\infty \int _0^\infty p(x,y|z) p_e(\nu |D,z_F) f_{\nu }(z) dz \,, \end{aligned}$$

or assuming Poissonian distributions

8 $$\begin{aligned} {p_0(x,y) = \sum _{\nu = 0}^\infty \int _0^\infty e^{-\kappa (z)}\frac{\left( \kappa (z)\right) ^x}{x!}e^{-\lambda (z)}\frac{\left( \lambda (z)\right) ^y}{y!}\frac{e^{- \frac{D}{z_F}}}{\nu !}\left( \frac{D}{z_F}\right) ^{\nu } f_{\nu }(z) dz \,,} \end{aligned}$$

for suitable functions $\kappa (z)$ and $\lambda (z)$. These quantities summarize the free-radical reactions that result in a lesion. It is a function of the type of ionizing particle, details of the track structure, radical diffusion, and reaction rates, the point in the cell cycle, and the chemical environment of the cell. In the following, we will explicitly model these functions so that they depend on chemical concentration.

The classical assumption, which has been also considered in Cordoni et al. (2021), is to assume such functions to be linear in *z*. Notable enough, it has been shown in Cordoni et al. (2022b) that, also assuming a Poissonian distribution on both $p_e$ and *p*, the resulting discrete probability density function (8) is not a Poisson distribution; as a matter of a fact, it has been shown to be a microdosimetric extension of the so-called *Neyman distribution* (Neyman 1939), which is a well-known distribution in radiobiological modeling to treat the number of radiation-induced DNA damages. To have a better grasp on the distribution (7), a stochastic chain of interconnected events can describe it: (i) given a certain dose *D* and fluence average specific energy $z_F$, a given random number of events $\nu$ is registered in a cell nucleus; then (ii) such $\nu$ events deposits a certain random specific energy $z=z_1+\dots +z_\nu$. At last, (iii) the specific energy deposited *z* induces a random number of lethal and sub-lethal lesions *y* and *x*.

## The spatial radiobiological model

The current section aims at generalizing the radiobiological model as introduced in Sect. 2 to consider a spatial measure-valued process. Consider a closed convex bounded regular enough domain $\textrm{Q}\subset \mathbb {R}^d$, $d \ge 1$, which should represent a cell nucleus. We assume that $\textrm{Q}$ has a smooth boundary $\partial \textrm{Q}$, and denote by *n*(*q*) the outward normal direction to the boundary $\partial \textrm{Q}$ at the point *q*. It is worth stressing that most of the subsequent analysis holds true also for non-convex domains. The convexity is required to ease the treatment regarding some sampling measures used later in the paper.

We consider two possible types of DNA damage, $\textrm{S}= \{\textrm{X},\textrm{Y}\}$, where $\textrm{X}$ denotes sub-lethal lesions and $\textrm{Y}$ are lethal lesions. We assume sub-lethal and lethal lesions can undergo three different pathways, *a*, *b*, and *r*, as introduced in Sect. 2.

We consider thus a process that lives in the state space

$$\begin{aligned} \textrm{P}:= \textrm{Q}\times \textrm{S}\ni P_i = \left( q_i,s_i\right) \,, \end{aligned}$$

encoding the i-th lesion position $q_i$ and type $s_i$. For a metric space *E*, we define by $\mathcal {M}_F(E)$ the space of finite measure over *E*, endowed with the weak topology; given a regular enough function $f : E \rightarrow \mathbb {R}$ and a measure $\nu \in \mathcal {M}_F(E)$, $\mathcal {M}_F(E)$ is equipped with

$$\begin{aligned} \left\langle f,\nu \right\rangle _E:=\int _E f(x)\nu (dx)\,. \end{aligned}$$

Also, we denote by $\mathcal {M}(E)$ the space of point measure over *E*, defined as

$$\begin{aligned} \mathcal {M}(E) := \left\{ \sum _{i=1}^N \delta _{x_i} \,:\, x_i \in E\,,\, N \in \mathbb {N} \right\} \,, \end{aligned}$$

equipped with, for $f : E \rightarrow \mathbb {R}$ and a measure $\nu \in \mathcal {M}(E)$,

$$\begin{aligned} \left\langle f,\nu \right\rangle _E:= \sum _{i=1}^N f(x_i)\,. \end{aligned}$$

In general, in the following, we will often consider either $E=\textrm{P}$ or $E=\textrm{Q}$; if no confusion is possible, we will omit the subscript in the scalar product.

Fix a finite time horizon $T<\infty$, for $t \in [0,T]$, we define the concentration measure of lesion at time *t*, as

9 $$\begin{aligned} \nu (t) := \sum _{i=1}^{N(t)} \delta _{P_i(t)} = \sum _{i=1}^{N(t)} \delta _{q_i(t)}\delta _{s_i}\,, \end{aligned}$$

with

$$\begin{aligned} N(t) = \left\langle \textbf{1},\nu (t) \right\rangle \,, \end{aligned}$$

the total number of lesions at time *t*. We further denote by $\nu ^\textrm{X}(t)$ and $\nu ^\textrm{Y}(t)$ the marginal distributions

10 $$\begin{aligned} \nu ^\textrm{X}(t)(\cdot ) := \nu (t)\left( \, \cdot \,,\textrm{X}\right) \,,\quad \nu ^\textrm{Y}(t)(\cdot ) := \nu (t)\left( \, \cdot \,,\textrm{Y}\right) \,. \end{aligned}$$

Analogously to the previous notation, $N^\textrm{X}(t)$, resp. $N^\textrm{Y}(t)$, denote the total number of lesions of type $\textrm{X}$, resp. $\textrm{Y}$, at time *t*.

Besides lesion concentration, we will often use a vector listing all lesions in the system; thus, given a system state $\nu (t)$, we denote by

$$\begin{aligned} \textrm{H}(\nu (t)):= & {} \left( \left( q^{1;\textrm{X}}(t),\textrm{X}\right) ,\dots ,\left( q^{N^\textrm{X}(t);\textrm{X}}(t),\textrm{X}\right) ,\right. \\{} & {} \left. \times \left( q^{1;\textrm{Y}}(t),\textrm{Y}\right) ,\dots ,\left( q^{N^\textrm{Y}(t);\textrm{Y}}(t),\textrm{Y}\right) ,0,\dots \right) \,, \end{aligned}$$

the position and type of all lesions in the system at time *t*. It is worth stressing that, since lesions of the same type are indistinguishable, the chosen ordering is arbitrary and there is no ambiguity in $\textrm{H}(\nu (t))$. We denote for short by $\textrm{H}^i(\nu (t)) \in \textrm{P}$, the $i-$th entry of the vector $\textrm{H}(\nu (t))$. With a similar notation, we denote

$$\begin{aligned} \begin{aligned} \textrm{H}(\nu ^{\textrm{X}}(t))&:= \left( q^{1;\textrm{X}}(t),\dots ,q^{N^\textrm{X}(t);\textrm{X}}(t),0,\dots \right) \,,\\ \textrm{H}(\nu ^{\textrm{Y}}(t))&:= \left( q^{1;\textrm{Y}}(t),\dots ,q^{N^\textrm{Y}(t);\textrm{Y}}(t),0,\dots \right) \,. \end{aligned} \end{aligned}$$

the vector containing only the positions of lesions of type $\textrm{X}$ and $\textrm{Y}$, respectively.

### The model

Each lesion *i*, characterized by its position and lesion type $P_i = \left( q_i,s_i\right)$, can move and undergo three different pathways. Such rates can be described by the system

11 $$\begin{aligned} {\left\{ \begin{array}{ll} &{} \textrm{X}\xrightarrow {\textrm{r}} \emptyset \,,\\ &{} \textrm{X}\xrightarrow {\textrm{a}} Y\,,\\ &{} \textrm{X}+ \textrm{X}\xrightarrow {\textrm{b}} {\left\{ \begin{array}{ll} \textrm{Y}&{} \text{ with } \text{ probability } \quad \textrm{p}\in [0,1]\,,\\ \emptyset &{} \text{ with } \text{ probability } \quad 1-\textrm{p}\in [0,1]\,,\\ \end{array}\right. }\,.\\ \end{array}\right. } \end{aligned}$$

and can be characterized as follows:

(i) - Repair each lesion in the class of sub-lethal lesions $\textrm{X}$ can be repaired at a rate
  $$\begin{aligned} \textrm{r}:\textrm{Q}\times \mathbb {R}\rightarrow \mathbb {R}_+\,,\quad \textrm{r}\left( q, \left\langle \Gamma ^{\textrm{r}}_q,\nu \right\rangle \right) \,, \end{aligned}$$
  that depends on the spatial position of the $i-$th lesion and on the concentration of the system via a suitable function $\Gamma ^{\textrm{r}}_q : \textrm{Q}\times \textrm{S}\rightarrow \mathbb {R}_+$ to be formally introduced later in the paper. A sub-lethal lesion that repairs disappear from the system. The *repair rate* $\textrm{r}$ is associated to a Poisson point measure
  $$\begin{aligned} \textrm{N}^\textrm{r}(ds,d\textbf{i},d\theta ) \quad \text{ on } \quad \mathbb {R}_+ \times \mathbb {N}_0 \times \mathbb {R}_+\,. \end{aligned}$$
  The index $\textbf{i}\in \mathbb {N}_0$ gives the sampled lesion in $\textrm{X}$ to repair. The corresponding intensity measure associated with $N^\textrm{r}$ is
  $$\begin{aligned} \lambda ^\textrm{r}(ds,d\textbf{i},d\theta ) := ds \otimes \left( \sum _{k \ge 0} \delta _k(\textbf{i})\right) \otimes d\theta \,. \end{aligned}$$
  We denote with $\tilde{\textrm{N}}^\textrm{r}$ the compensated Poisson measure defined as
  $$\begin{aligned} \tilde{\textrm{N}}^\textrm{r}(ds,d\textbf{i},d\theta ) := \textrm{N}(ds,d\textbf{i},d\theta ) - \lambda ^\textrm{r}(ds,d\textbf{i},d\theta )\,. \end{aligned}$$
(ii) - Death each lesion in the class of sub-lethal lesions $\textrm{X}$ can die at a rate
  $$\begin{aligned} \textrm{a}:\textrm{Q}\times \mathbb {R}\rightarrow \mathbb {R}_+\,,\quad \textrm{a}\left( q, \left\langle \Gamma ^\textrm{a}_q,\nu \right\rangle \right) \,, \end{aligned}$$
  that depends on the spatial position of the $i-$th lesion and on the concentration of the system via a suitable function $\Gamma ^\textrm{a}_q : \textrm{Q}\times \textrm{S}\rightarrow \mathbb {R}_+$ to be formally introduced later in the paper. A sub-lethal lesion at position ${q_1 \in \textrm{Q}}$ that dies generates a lethal lesion $\textrm{Y}$ at a new position ${q \in \textrm{Q}}$ according to the probability distribution ${m^\textrm{a}(q|q_1)}$. The *death rate* $\textrm{a}$ is associated to a Poisson point measure
  $$\begin{aligned} \textrm{N}^\textrm{a}(ds,d\textbf{i},dq,d\theta _1,d\theta _2)\quad \text{ on } \quad \mathbb {R}_+ \times \mathbb {N}_0 \times \textrm{Q}\times \mathbb {R}_+ \times \mathbb {R}_+\,. \end{aligned}$$
  The index $\textbf{i}\in \mathbb {N}_0$ gives the sampled lesion in $\textrm{X}$ to die and become a lethal lesion in $\textrm{Y}$ in position *q* sampled from $m^\textrm{a}(q|{q_i})$, $q \in \textrm{Q}$. The corresponding intensity measure associated with $N^\textrm{a}$ is
  $$\begin{aligned} \lambda ^\textrm{a}(ds,d\textbf{i},d\theta _1,d\theta _2) := ds \otimes \left( \sum _{k \ge 0} \delta _k(\textbf{i})\right) \otimes dq \otimes d\theta _1 \otimes d\theta _2\,. \end{aligned}$$
  We denote with $\tilde{\textrm{N}}^\textrm{a}$ the compensated Poisson measure defined as
  $$\begin{aligned} \tilde{\textrm{N}}^\textrm{a}(ds,d\textbf{i},d\theta _1,d\theta _2) := \textrm{N}^\textrm{a}(ds,d\textbf{i},d\theta _1,d\theta _2) - \lambda ^\textrm{a}(ds,d\textbf{i},d\theta _1,d\theta _2)\,. \end{aligned}$$
(iii) - Pairwise interaction two lesions in the class of sub-lethal lesions $\textrm{X}$ can interact at a rate
  $$\begin{aligned} \textrm{b}:\textrm{Q}\times \textrm{Q}\times \mathbb {R}\rightarrow \mathbb {R}_+\,,\quad \textrm{b}\left( q_1,q_2, \left\langle \Gamma ^\textrm{b}_{q_1,q_2},\nu \right\rangle \right) \,, \end{aligned}$$
  that depends on the spatial position of the $(i_1, i_2)-$th lesions and on the concentration of the system via a suitable function $\Gamma ^\textrm{b}_{q_1,q_2} : \textrm{Q}\times \textrm{S}\rightarrow \mathbb {R}_+$ to be formally introduced later in the paper. Two sub-lethal lesions that interact can either (i) die with probability *p*, generating a lethal lesion $\textrm{Y}$ at a new position *q* according to the distribution $m^\textrm{b}(q|q_1,q_2)$, $q \in \textrm{Q}$, or (ii) repair with probability $1-p$ and disappear from the system. The probability *p* depends also on the positions of the sampled lesions, namely
  $$\begin{aligned} \textrm{p}:\textrm{Q}\times \textrm{Q}\rightarrow [0,1]\,,\quad \textrm{p}\left( q_1,q_2\right) \,. \end{aligned}$$
  In the following, we consider three relevant scenarios for the Poisson point measure associated with the *pairwise interaction rate* $\textrm{b}$ depending on the considered sample measure $m^b(q|q_1,q_2)$.

1. if, for any $q_1$ and $q_2 \in \textrm{Q}$, the sampling measure $m^b(\cdot |q_1,q_2)$ is absolutely continuous with respect to the Lebesgue measure, then the *pairwise interaction rate* $\textrm{b}$ is associated to two Poisson point measures
  $$\begin{aligned} \begin{aligned} \textrm{N}^{\textrm{b};p}(ds,d\textbf{i},dq,d\theta _1,d\theta _2)\quad&\text{ on } \quad \mathbb {R}_+ \times \mathbb {N}_0 \times \mathbb {N}_0 \times \textrm{Q}\times \mathbb {R}_+ \times \mathbb {R}_+\,,\\ \textrm{N}^{\textrm{b};1-p}(ds,d\textbf{i},d\theta )\quad&\text{ on } \quad \mathbb {R}_+ \times \mathbb {N}_0 \times \mathbb {N}_0 \times \mathbb {R}_+\,. \end{aligned} \end{aligned}$$
  The index $\textbf{i}= (\textbf{i}_1,\textbf{i}_2) \in \mathbb {N}_0 \times \mathbb {N}_0$ gives the sampled lesions in $\textrm{X}$ to either become a lethal lesion in $\textrm{Y}$ in position *q* sampled from $m(q|q_{i_1},q_{i_2})$, $q\,,\,q_{i_1}\,,\,q_{i_2} \in \textrm{Q}$, or repair and be removed from the system. The corresponding intensity measures associated with $\textrm{N}^{\textrm{b};p}$ and $\textrm{N}^{\textrm{b};1-p}$ are
  $$\begin{aligned} \begin{aligned} \lambda ^{\textrm{b};p} (ds,d\textbf{i},dq,d\theta _1,d\theta _2)&:= ds \otimes \left( \sum _{k \ge 0} \delta _k(\textbf{i}_1) \wedge \sum _{k \ge 0} \delta _k(\textbf{i}_2) \right) \otimes dq \otimes d\theta _1 \otimes d\theta _2\,,\\ \lambda ^{\textrm{b};1-p} (ds,d\textbf{i},d\theta )&:= ds \otimes \left( \sum _{k \ge 0} \delta _k(\textbf{i}_1) \wedge \sum _{k \ge 0} \delta _k(\textbf{i}_2) \right) \otimes d\theta \,. \end{aligned} \end{aligned}$$
  We denote with $\tilde{\textrm{N}}^\textrm{b}$ the compensated Poisson measure defined as
  $$\begin{aligned} \begin{aligned} \tilde{\textrm{N}}^{\textrm{b};p}(ds,d\textbf{i},dq,d\theta _1,d\theta _2)&:= \textrm{N}^{\textrm{b};p}(ds,d\textbf{i},dq,d\theta _1,d\theta _2) - \lambda ^{\textrm{b};p} (ds,d\textbf{i},dq,d\theta _1,d\theta _2)\,,\\ \tilde{\textrm{N}}^{\textrm{b};1-p}(ds,d\textbf{i},d\theta )&:= \textrm{N}^{\textrm{b};1-p}(ds,d\textbf{i},d\theta ) - \lambda ^{\textrm{b};1-p} (ds,d\textbf{i},d\theta )\,.\\ \end{aligned} \end{aligned}$$
2. if the sampling measure $m^b(\,\cdot \,|q_{i_1},q_{i_2})$ is of the form
  12 $$\begin{aligned} \tilde{m}^b\left( \alpha q_{i_1} + (1-\alpha )q_{i_2} \right) \,,\quad \alpha \in [0,1]\,, \end{aligned}$$
  for a probability density function $\tilde{m} : [0,1] \rightarrow \mathbb {R}$, then the *pairwise interaction rate* $\textrm{b}$ is associated to two Poisson point measures
  $$\begin{aligned} \begin{aligned} \textrm{N}^{\textrm{b};p}(ds,d\textbf{i},d\alpha ,d\theta _1,d\theta _2)\quad&\text{ on } \quad \mathbb {R}_+ \times \mathbb {N}_0 \times \mathbb {N}_0 \times [0,1] \times \mathbb {R}_+ \times \mathbb {R}_+\,,\\ \textrm{N}^{\textrm{b};1-p}(ds,d\textbf{i},d\theta )\quad&\text{ on } \quad \mathbb {R}_+ \times \mathbb {N}_0 \times \mathbb {N}_0 \times \mathbb {R}_+\,, \end{aligned} \end{aligned}$$
  with corresponding associated intensity measures
  $$\begin{aligned} \begin{aligned} \lambda ^{\textrm{b};p} (ds,d\textbf{i},d\alpha ,d\theta _1,d\theta _2)&:= ds \otimes \left( \sum _{k \ge 0} \delta _k(\textbf{i}_1) \wedge \sum _{k \ge 0} \delta _k(\textbf{i}_2) \right) \otimes d\alpha \otimes d\theta _1 \otimes d\theta _2\,,\\ \lambda ^{\textrm{b};1-p} (ds,d\textbf{i},d\theta )&:= ds \otimes \left( \sum _{k \ge 0} \delta _k(\textbf{i}_1) \wedge \sum _{k \ge 0} \delta _k(\textbf{i}_2) \right) \otimes d\theta \,. \end{aligned} \end{aligned}$$
3. if the sampling measure $m^b(\,\cdot \,|q_{i_1},q_{i_2})$ takes positive values over a discrete set $\{1,\dots ,J\}$, $J < \infty$, that is it is of the form
  13 $$\begin{aligned} p_j := \tilde{m}^b \left( \alpha _j q_{i_1} + (1-\alpha _j)q_{i_2} \right) \,,\quad \alpha _j \in [0,1]\quad \text{ and } \quad \sum _{j=1}^J p_j=1\,, \end{aligned}$$
  then the *pairwise interaction rate* $\textrm{b}$ is associated to two Poisson point measures
  $$\begin{aligned} \begin{aligned} \textrm{N}^{\textrm{b};p}(ds,d\textbf{i},d\alpha _j,d\theta _1,d\theta _2)\quad&\text{ on } \quad \mathbb {R}_+ \times \mathbb {N}_0 \times \mathbb {N}_0 \times \{1,\dots ,J\} \times \mathbb {R}_+ \times \mathbb {R}_+\,,\\ \textrm{N}^{\textrm{b};1-p}(ds,d\textbf{i},d\theta )\quad&\text{ on } \quad \mathbb {R}_+ \times \mathbb {N}_0 \times \mathbb {N}_0 \times \mathbb {R}_+\,. \end{aligned} \end{aligned}$$
  with corresponding associated intensity measures
  $$\begin{aligned} \begin{aligned} \lambda ^{\textrm{b};p} (ds,d\textbf{i},d\alpha _j,d\theta _1,d\theta _2)&:= ds \otimes \left( \sum _{k \ge 0} \delta _k(\textbf{i}_1) \wedge \sum _{k \ge 0} \delta _k(\textbf{i}_2) \right) \\&\quad \otimes \sum _{j=1}^J \delta _j(\alpha _j) \otimes d\theta _1 \otimes d\theta _2\,,\\ \lambda ^{\textrm{b};1-p} (ds,d\textbf{i},d\theta )&:= ds \otimes \left( \sum _{k \ge 0} \delta _k(\textbf{i}_1) \wedge \sum _{k \ge 0} \delta _k(\textbf{i}_2) \right) \otimes d\theta \,. \end{aligned} \end{aligned}$$

#### Remark 3.1

It is worth stressing that, the three different cases for the sampling measure $m^b(\,\cdot \,|q_1,q_2)$ have been chosen to include possible relevant examples. However, other choice can be made so that the next theory still apply. The choice of restricting to some particular case has been made to cover specific examples in the application under study.

In particular, the case in Eq. (12) considers the situation where the new lethal lesion $\textrm{Y}$ created is on the segment connecting $q_1$ and $q_2$ on a position sampled according to $\tilde{m}^b$. A meaningful choice would be to consider, for instance, $\tilde{m}^b$ to be the uniform distribution over [0, 1].

Further, concerning the sampling measure in Eq. (13), it assumes that the new lethal lesion is generated on some discrete points on the segment connecting $q_1$ and $q_2$ according to a certain probability; in this case, $m^b$ is a discrete probability distribution. In such a scenario, two relevant choices are to consider:

(i) $J=2$, $\alpha _1=1$ and $\alpha _2=0$, so that the new lethal lesion is created at $q_1$; sampling at position $q_2$ can be assumed with the choice $\alpha _1=0$ and $\alpha _2=1$;
(ii) $J=1$, $\alpha _1=\alpha _2=\frac{1}{2}$, and the new lethal lesion is generated in the middle point between $q_1$ and $q_2$.

In the following, with a slight abuse of notation, we will use the notation formally valid for the case (*iii*).1, specifying how the notation should be changed accordingly to consider either case (*iii*).2 or (*iii*).3.

(iv) - Spatial diffusion each lesion of type $\textrm{X}$ and $\textrm{Y}$ moves around the domain $\textrm{Q}$ with diffusion term
  $$\begin{aligned} \begin{aligned}&\sigma ^\textrm{X}: \textrm{Q}\rightarrow \mathbb {R}^{d \times d} \,,\quad \sigma ^\textrm{X}\left( q\right) \,,\\&\sigma ^\textrm{Y}: \textrm{Q}\rightarrow \mathbb {R}^{d \times d} \,,\quad \sigma ^\textrm{Y}\left( q\right) \,,\\ \end{aligned} \end{aligned}$$
  and drift term

$$\begin{aligned} \begin{aligned}&\mu ^\textrm{X}: \textrm{Q}\rightarrow \mathbb {R}^d \,,\quad \mu ^\textrm{X}\left( q\right) \,,\\&\mu ^\textrm{Y}: \textrm{Q}\rightarrow \mathbb {R}^d\,,\quad \mu ^\textrm{Y}\left( q\right) \,. \end{aligned} \end{aligned}$$

In the following, we will also denote

$$\begin{aligned} \begin{aligned}&\Sigma ^\textrm{X}: \textrm{Q}\rightarrow \mathcal {S}_+\left( \mathbb {R}^d \right) \,,\quad \Sigma ^\textrm{X}\left( q\right) := \sigma ^\textrm{X}\left( q\right) \left( \sigma ^\textrm{X}\left( q\right) \right) ^T\,,\\&\Sigma ^\textrm{Y}: \textrm{Q}\rightarrow \mathcal {S}_+\left( \mathbb {R}^d \right) \,,\quad \Sigma ^\textrm{Y}\left( q\right) := \sigma ^\textrm{Y}\left( q\right) \left( \sigma ^\textrm{Y}\left( q\right) \right) ^T\,,\\ \end{aligned} \end{aligned}$$

with $\mathcal {S}_+\left( \mathbb {R}^d \right)$ the space of symmetric non-negative $d \times d$ matrices.

To describe lesion motion, we introduce a countable collection of standard independent Brownian motion $\left( W^{n;\textrm{X}}(t)\right) _{n \in \mathbb {N}}$ and $\left( W^{n;\textrm{Y}}(t)\right) _{n \in \mathbb {N}}$ on $\mathbb {R}^d$. Brownian motion is assumed to reflect with normal derivative at the boundary of the domain $\textrm{Q}$. In particular, denote by $T_{n}$ and $T_{n+1}$ two successive jump times of the process $\nu$, and assume that at time $T_n$ we have $N^\textrm{X}(T_n)$, resp. $N^\textrm{Y}(T_n)$, lesions of type $\textrm{X}$, resp. $\textrm{Y}$. It is worth stressing that in $t \in [T_{n},T_{n+1})$, the number of lesions remains constant so that the process is solely subject to the diffusive component. Thus, for any $t \in [T_{n},T_{n+1})$ each lesion evolves according to the following SDE with reflection at the boundaries

14 $$\begin{aligned} {\left\{ \begin{array}{ll} \textrm{X}^{i_\textrm{X}}(t) &{}= \textrm{X}^{i_\textrm{X}}(T_n) + \int _{T_n}^{t} \sigma ^\textrm{X}\left( \textrm{X}^{i_\textrm{X}}(s)\right) dW^{i_\textrm{X};\textrm{X}}(s)+ \int _{T_n}^{t} \mu ^\textrm{X}\left( \textrm{X}^{i_\textrm{X}}(s) \right) ds - \kappa ^{i_\textrm{X}}(t)\,,\\ |\kappa ^{i_\textrm{X}}|(t) &{}= \int _{T_n}^{t} \mathbb {1}_{\left\{ \textrm{X}^{i_\textrm{X}}(s) \in \partial \textrm{Q}\right\} } d|\kappa ^{i_\textrm{X}}|(s)\,,\quad \kappa ^{i_\textrm{X}}(t) = \int _{T_n}^{t} n\left( \textrm{X}^{i_\textrm{X}}(s) \right) d|\kappa ^{i_\textrm{X}}|(s)\,,\\ \textrm{X}^{i_\textrm{X}}(t) &{}\in \bar{\textrm{Q}}\,,\quad i_\textrm{X}{=} 1,\dots ,N^\textrm{X}(t)\,,\\ \textrm{Y}^{i_\textrm{Y}}(t) &{}= \textrm{Y}^{i_\textrm{Y}}(T_n) + \int _{T_n}^{t} \sigma ^\textrm{Y}\left( \textrm{Y}^{i_\textrm{Y}}(s)\right) dW^{i_\textrm{Y};\textrm{Y}}(s)+ \int _{T_n}^{t} \mu ^\textrm{Y}\left( \textrm{Y}^{i_\textrm{Y}}(s)\right) ds - \kappa ^{i_\textrm{Y}}(t)\,,\\ |\kappa ^{i_\textrm{Y}}|(t) &{}= \int _{T_n}^{t} \mathbb {1}_{\left\{ \textrm{Y}^{i_\textrm{Y}}(s) \in \partial \textrm{Q}\right\} } d|\kappa ^{i_\textrm{Y}}|(s)\,,\quad \kappa ^{i_\textrm{Y}}(t) = \int _{T_n}^{t} n\left( \textrm{Y}^{i_\textrm{Y}}(s) \right) d|\kappa ^{i_\textrm{Y}}|(s)\,, \\ \textrm{Y}^{i_\textbf{Y}}(t) &{}\in \bar{\textrm{Q}}\,,\quad i_\textrm{Y}{=} 1,\dots ,N^\textrm{Y}(t)\,,\\ \end{array}\right. } \end{aligned}$$

where we denoted by *dW*(*t*) the integration in the sense of Itô.

#### Remark 3.2

Above, for the sake of simplicity, we have considered the case where each lesion moves according to a single Brownian motion and that different Brownian motions are independent. From a purely biological standpoint, it would be more meaningful to either assume that a certain group of lesions moves according to the same Brownian motion or to consider correlated Brownian motions. The diffusion of lesions around the cell nucleus described the drift of DNA filaments inside the cell nucleus (Nykypanchuk et al. 2002; Serag and Habuchi 2017). The former case can be straightforwardly included in the treatment done in the paper, whereas for the latter, a slight modification is needed. In fact (Shreve 2004), using a Cholesky decomposition, a multidimensional vector of correlated Brownian motions *B* motion can be written in terms of uncorrelated Brownian motions as

$$\begin{aligned} dB(t) = C(t) dW(t)\,, \end{aligned}$$

for a uncorrelated Brownian motion *W*, being *C* a lower triangular matrix defined by the relation

$$\begin{aligned} \rho (t) = C(t) C^T(t)\,, \end{aligned}$$

with $\rho$ the correlation matrix

$$\begin{aligned} d\langle B^i,B^j\rangle (t) = \rho ^{ij}(t)dt\,. \end{aligned}$$

Therefore, the correlated case can be reduced to the uncorrelated case treated in the present paper.

In the following, we will consider a filtered and complete probability space $\left( \Omega ,\mathcal {F},\left( \mathcal {F}_t\right) _{t \in \mathbb {R}_+},\mathbb {P}\right)$ satisfying standard assumptions, namely right–continuity and saturation by $\mathbb {P}$–null sets. In particular, $\left( \mathcal {F}_t\right) _{t \in \mathbb {R}_+}$ is the filtration generated by the processes defined in $(i)-(ii)-(iii)-(iv)$ as well as a $\mathcal {M}\times \mathcal {M}-$valued initial distribution ${\varvec{\nu }}_0 = (\nu ^\textrm{X}_0,\nu ^\textrm{Y}_0)$.

#### Remark 3.3

Notice that, compared to the original interaction rates as introduced in Cordoni et al. (2021), we included in the present version of the model a further possible pathway, namely

$$\begin{aligned} \textrm{X}+ \textrm{X}\xrightarrow {\textrm{b}} {\left\{ \begin{array}{ll} \textrm{Y}&{} \text{ with } \text{ probability } \quad \textrm{p}\in [0,1]\,,\\ \emptyset &{} \text{ with } \text{ probability } \quad 1-\textrm{p}\in [0,1]\,,\\ \end{array}\right. }\,.\\ \end{aligned}$$

This is done since, as noted in early versions of advanced radiobiological models (Sachs et al. 1992), pairwise interaction of damages, can result also in correct repairs; such a process is called, for instance, in Sachs et al. (1992) as *complete exchange*. $\triangle$

Through the paper, we will assume the following hypothesis to hold:

#### Hypothesis 3.4

1. Jump components:
  (2.i) the *repair rate* $\textrm{r}$ is uniformly bounded over compact subsets, that is for $N \ge 0$ it exists $\bar{\textrm{r}}$ such that
    $$\begin{aligned} \sup _{q \in \textrm{Q}} \sup _{v \in [0,N]} \textrm{r}(q,v)< \bar{\textrm{r}} < \infty \,; \end{aligned}$$
  (2.ii) the *death rate* $\textrm{a}$ satisfies a linear growth condition, that is, there exists a positive constant $\bar{\textrm{a}}$ such that, for all $q \in \textrm{Q}$ it holds
    $$\begin{aligned} \begin{aligned}&0 \le \textrm{a}(q,v) \le \bar{\textrm{a}}(1+|v|)\,;\\ \end{aligned} \end{aligned}$$
  (2.iii) the *pairwise interaction rate* $\textrm{b}$ satisfies a linear growth condition, that is, there exists a positive constant $\bar{\textrm{b}}$ such that, for all $q_1$ and $q_2 \in \textrm{Q}$, it holds
    $$\begin{aligned} \begin{aligned}&0 \le \textrm{b}(q_1,q_2,v) \le \bar{\textrm{b}}(1+|v|)\,;\\ \end{aligned} \end{aligned}$$
  (2.iv) the *pairwise interaction death* $\textrm{p}$ is a probability, that is, for all $q_1$ and $q_2 \in \textrm{Q}$, it holds
    $$\begin{aligned} \begin{aligned}&\textrm{p}(q_1,q_2) \in [0,1]\,;\\ \end{aligned} \end{aligned}$$
2. Diffusive components:
  (2.i) there exist positive constants $L^\textrm{X}$ and $L^\textrm{Y}$ such that, for any $q_1$, $q_2 \in \textrm{Q}$, it holds
    $$\begin{aligned} \begin{aligned}&|\sigma ^\textrm{X}(q_1)-\sigma ^\textrm{X}(q_2)|+|\mu ^\textrm{X}(q_1)-\mu ^\textrm{X}(q_2)|\le L^\textrm{X}|q_1 - q_2| \,,\\&|\sigma ^\textrm{Y}(q_1)-\sigma ^\textrm{Y}(q_2)|+|\mu ^\textrm{Y}(q_1)-\mu ^\textrm{Y}(q_2)|\le L^\textrm{Y}|q_1 - q_2| \,. \end{aligned} \end{aligned}$$
3. Kernel components:
  (3.i) for all $q\,,\,q_1\,,\,q_2 \in \textrm{Q}$ the functions $\Gamma ^\textrm{r}_q : \textrm{Q}\times \textrm{S}\rightarrow \mathbb {R}_+$, $\Gamma ^\textrm{a}_q : \textrm{Q}\times \textrm{S}\rightarrow \mathbb {R}_+$ and $\Gamma ^\textrm{b}_{q_1,q_2} : \textrm{Q}\times \textrm{S}\rightarrow \mathbb {R}_+$ are continuous and uniformly bounded, that is, there exist constants $\bar{\Gamma }^\textrm{r}$, $\bar{\Gamma }^\textrm{a}$ and $\bar{\Gamma }^\textrm{b}$ such that
    $$\begin{aligned} \begin{aligned}&\sup _{q \in \textrm{Q}}\, \sup _{(\bar{q},\bar{s}) \in \textrm{Q}\times \textrm{S}} \Gamma ^\textrm{r}_q (\bar{q},\bar{s})< \bar{\Gamma }^\textrm{r}<\infty \,,\\&\sup _{q \in \textrm{Q}}\, \sup _{(\bar{q},\bar{s}) \in \textrm{Q}\times \textrm{S}} \Gamma ^\textrm{a}_q (\bar{q},\bar{s})< \bar{\Gamma }^\textrm{a}<\infty \,,\\&\sup _{q_1\,,\,q_2 \in \textrm{Q}}\, \sup _{(\bar{q},\bar{s}) \in \textrm{Q}\times \textrm{S}} \Gamma ^\textrm{b}_{q_1,q_2} (\bar{q},\bar{s})< \bar{\Gamma }^\textrm{b}<\infty \,;\\ \end{aligned} \end{aligned}$$
  (3.ii) the sampling measure ${m^\textrm{b}(q|q_1,q_2)}$ or ${\tilde{m}^\textrm{b}(q)}$ is either an absolutely continuous or discrete probability density, that is
    $$\begin{aligned}{} & {} \int _\textrm{Q}m^\textrm{b}(q|q_1,q_2)dq =1\,,\quad \int _0^1 \tilde{m}^\textrm{b}(\alpha q_1 + (1-\alpha )q_2)d\alpha =1\,,\\{} & {} \quad \sum _{j=1}^J \tilde{m}^\textrm{b}(\alpha _j q_1 + (1-\alpha _j)q_2)=1\,. \end{aligned}$$
    The sampling measure ${m^\textrm{a}}(q|q_1)$ is an absolutely continuous probability density, that is
    $$\begin{aligned} {\int _\textrm{Q}m^\textrm{a}(q|q_1)dq =1\,.} \end{aligned}$$

#### Remark 3.5

It is worth remarking that, slightly adapting the Poisson point measure introduced in Hypothesis 3.4 (*ii*) to be

$$\begin{aligned} \textrm{N}^\textrm{a}(ds,d\textbf{i},d\theta _1)\quad \text{ on } \quad \mathbb {R}_+ \times \mathbb {N}_0 \times \mathbb {R}_+\,, \end{aligned}$$

we could assume that the new lethal lesion is formed at position $q \in \textrm{Q}$ where the reaction $\textrm{a}$ happened, allowing thus to consider the trivial case of the sampling measure

$$\begin{aligned} m^\textrm{a}(q|q_1) = \delta _{q_1}(q)\,. \end{aligned}$$

For ease of notation, we will not consider this case in the following, following instead on the case of $m^\textrm{a}(q|q_1)$ being absolutely continuous with respect to the Lebesgue measure. $\triangle$

In the following, we consider the next class of cylindrical test functions: for $F \in \mathcal {C}^2_b (\mathbb {R}\times \mathbb {R})$, that is, *F* is bounded with continuous and bounded second order derivative, and for $f^\textrm{X}\,,\,f^\textrm{Y}\in \mathcal {C}^{2}_0(\textrm{Q})$, that is $f^\textrm{X}$ and $f^\textrm{Y}$ are continuous with bounded second order derivative in the domain variable $\textrm{Q}$, satisfying $\nabla _q f(q) \cdot n(q)=0$, for ${\varvec{\nu }} = (\nu ^\textrm{X},\nu ^\textrm{Y})$, we consider $F_{(f^\textrm{X},f^\textrm{Y})}:\mathcal {M}\times \mathcal {M}\rightarrow \mathbb {R}$ of the form

15 $$\begin{aligned} F_{(f^\textrm{X},f^\textrm{Y})}(\nu ) = F \left( \left\langle f^\textrm{X},\nu ^\textrm{X} \right\rangle , \left\langle f^\textrm{Y},\nu ^\textrm{Y} \right\rangle \right) \,. \end{aligned}$$

In the following, we will denote by $\frac{\partial }{\partial x}$, resp. $\frac{\partial }{\partial x}$, the derivative with respect to the first argument, resp. the second argument, of the function *F*. Also, $\nabla$, resp. $\Delta$, resp. *Tr*, resp. *Hess*, denotes the gradient with respect to the space variable *q*, resp. the Laplacian operator with respect to the space variable *q*, resp. the trace operator, resp. the Hessian matrix. Cylindrical functions (15) are a standard class generating the set of bounded and measurable functions from $\mathcal {M}\times \mathcal {M}$ into $\mathbb {R}$ (Fontbona and Méléard 2015; Champagnat and Méléard 2007; Dawson et al. 1993).

#### Remark 3.6

(i) The kernels $\Gamma ^\textrm{r}_q$, $\Gamma ^\textrm{a}_q$ and $\Gamma ^\textrm{b}_{q_1.q_2}$ account for changes in the reaction rates due to the state of the system. The most intuitive case would be to assume that the system’s mass near the position where the reactions $\textrm{a}$ and $\textrm{r}$ take place affects the overall rate. A natural choice for the kernel $\Gamma ^\textrm{r}_q$ and $\Gamma ^\textrm{a}_q$ would be to assume that only nearby mass affects the overall rate; in such a case, we have, for $q \in \textrm{Q}$,
  $$\begin{aligned} \Gamma ^\textrm{r}_q (\bar{q},\bar{s}) := \mathbb {1}_{\left\{ |q-\bar{q}|<\epsilon \right\} } (\bar{q},\bar{s})\,. \end{aligned}$$
  Therefore, only lesions at most distant $\epsilon$ from the position *q* where the reaction happens to participate in the reaction. For the biological interpretation of the above kernels, besides the already known clustered effect of DNA lesions, there is experimental evidence that the repair rate of DNA lesions created by densely ionizing radiation, such as LET, is different from the repair of lesions induced by sparsely ionizing radiation, such as X-rays (Russ et al. 2022; Guerra Liberal et al. 2023). This difference could be imputed to the different complexity of the created lesions. To date, this dependence of the repair rate on the type of radiation is often neglected in mechanistic modeling. The kernels above introduced could be thus used to include such effects in the pathways considered in the present model.
(ii) Regarding the *pairwise interaction rate* $\textrm{b}$, it is natural to assume that $\textrm{b}$ depends only on the separation distance between two lesions, that is, it exists a function
  $$\begin{aligned} \bar{\textrm{b}} :\mathbb {R}\rightarrow \mathbb {R}_+\,,\quad \bar{\textrm{b}}\left( \mathfrak {q}\right) \,, \end{aligned}$$
  such that
  $$\begin{aligned} \textrm{b}\left( q_1,q_2\right) = \textrm{b}\left( q_2,q_1\right) = \bar{\textrm{b}}\left( |q_1 - q_2|\right) \,. \end{aligned}$$
  Further, it is natural to assume that the closer two lesions are, the more likely they interact. Therefore, relevant choices for the rate $\textrm{b}$ are, for instance a step interaction rate
  16 $$\begin{aligned} \bar{\textrm{b}}\left( \mathfrak {q}\right) := \hat{\textrm{b}} \mathbb {1}_{\left\{ |\mathfrak {q}|<\varepsilon \right\} }\,, \end{aligned}$$
  or a Gaussian rate
  17 $$\begin{aligned} \bar{\textrm{b}}\left( \mathfrak {q}\right) := \frac{\hat{\textrm{b}}}{\sqrt{2 \pi \varepsilon ^2}}e^{-\frac{|\mathfrak {q}|^2}{2\varepsilon ^2}} \,. \end{aligned}$$
  Whereas the former rate (16) models the case where only lesion closer than $\varepsilon$ can interact, the latter rate (17) considers that the rate of interaction decreases exponentially as the lesions are more distant from each other. At last, as noted in Kellerer and Rossi (1974), enhanced short-range interaction can be modelled using
  18 $$\begin{aligned} \bar{\textrm{b}}\left( \mathfrak {q}\right) := \frac{\hat{\textrm{b}}_1}{\sqrt{2 \pi \varepsilon ^2_1}}e^{-\frac{|\mathfrak {q}|^2}{2\varepsilon ^2_1}} + \frac{\hat{\textrm{b}}_2}{\sqrt{2 \pi \varepsilon ^2_2}}e^{-\frac{|\mathfrak {q}|^2}{2\varepsilon ^2_2}} \,, \end{aligned}$$
  for suitable constants, so that the interaction rate declines fast but still has a fat tail at larger distances. Similarly, it is reasonable to assume that $\textrm{p}$ depends on the distance between the interacting lesions.
(iii) The sampling measures introduced in Eqs. (12)–(13) can be approximated to recover the case of absolute continuous measure considered in (*iii*).1. Denote in fact, by $\mathfrak {m}_\varepsilon$ a standard mollified (Friedrichs 1944), that is a positive, smooth, and compactly supported function on [0, 1], so that
  (i) $$\int _{\textrm{Q}} \mathfrak {m}_\varepsilon (q) dq =1$$;
  (ii) $\lim _{\varepsilon \rightarrow 0} \mathfrak {m}_\varepsilon (q) = \delta _0(q)$, where $\delta _0$ is the Dirac delta centered in 0 and the limit is taken in the sense of Schwartz distribution.
  Equation (12) can be represented compactly as
  $$\begin{aligned} m^\textrm{b}(q|q_1,q_2) = \tilde{m}^\textrm{b}(q)\delta _{\alpha q_1 + (1-\alpha )q_2}(q)\,,\quad \alpha \in [0,1]\,. \end{aligned}$$
  Therefore, the sampling measure
  $$\begin{aligned} \tilde{m}^\textrm{b}(q)\delta _{\alpha q_1 + (1-\alpha )q_2}(q)*\mathfrak {m}_\varepsilon (q)\,, \end{aligned}$$
  where we have defined by $*$ the convolution operator, is absolutely continuous with respect to the Lebesgue measure, so that it is included in the case (*iii*).1 and converges to $\tilde{m}^b$ as $\varepsilon \rightarrow 0$. A similar argument holds for the case (*iii*).3, where the sampling measure reads as
  $$\begin{aligned} m^b(q|q_1,q_2) = \sum _{j=1}^J p_j \delta _{\alpha _j q_1 + (1-\alpha _j)q_2}(q)\,,\quad \alpha _j \in [0,1]\quad \text{ and } \quad \sum _{j=1}^J p_j=1\,, \end{aligned}$$
  and considering
  $$\begin{aligned} \sum _{j=1}^J p_j \delta _{\alpha _j q_1 + (1-\alpha _j)q_2}(q)*\mathfrak {m}_\varepsilon (q)\,, \end{aligned}$$
  it is possible to approximate $m^b$ with an absolutely continuous measure.
(iv) regarding the sampling measure $m^\textrm{a}(q|q_1)$, a reasonable choice would be to assume that the new particle is generated in an open ball centered around $q_1$, that is
  $$\begin{aligned} m^\textrm{a}(q|q_1) = \tilde{m}^\textrm{a}(q) \mathbb {1}_{\left\{ |q-q_1|<\varepsilon \right\} }\,,\quad \varepsilon >0\,, \end{aligned}$$
  for a given probability density $\tilde{m}^\textrm{a}(q)$. The density $\tilde{m}^\textrm{a}(q)$ can take for instance two different assumptions: it can be uniform, implying that a new lesion can occur with equal probability at any point within a distance of $\varepsilon$ from the initial lesion’s position that initiated the reaction. Alternatively, it can follow a bell-shaped distribution centered around $q_1$, indicating a higher likelihood of generating a new lesion in close proximity to the original lesion’s location.

$$\triangle $$

With the previous notation, we can thus introduce the following weak representation for the spatial radiation-induced DNA lesion repair model, given $f^\textrm{X}$ and $f^\textrm{Y}\in C^2_0(\mathbb {R})$, we have19

#### Remark 3.7

For the sake of brevity, Eq. (19) is formulated using the case 3.4(*iii*).1. Nonetheless:

(iii).2 If the sampling measure (12) is considered, then the integral terms with respect to the Poisson point measure $\textrm{N}^{\textrm{b};p}$ becomes
  $$\begin{aligned} \begin{aligned}&\int _0^t \int _{\mathbb {N}_0^2} \int _{0}^1 \int _{\mathbb {R}_+^2} \left[ \left\langle f^\textrm{Y},\nu ^{\textrm{Y}}(s_-) + \delta _{q} \right\rangle - \left\langle f^\textrm{Y},\nu ^{\textrm{Y}}(s_-) \right\rangle \right] \mathbb {1}_{\left\{ i_1< i_2 \le N^\textrm{X}(s_-)\right\} } \\&\quad \times \mathbb {1}_{\left\{ \theta _1 \le p\left( \textrm{H}^{i_1}\left( \nu ^\textrm{X}(s_-)\right) ,\textrm{H}^{i_2}\left( \nu ^\textrm{X}(s_-)\right) \right) \textrm{b}\left( \textrm{H}^{i_1}\left( \nu ^\textrm{X}(s_-)\right) ,\textrm{H}^{i_2}\left( \nu ^\textrm{X}(s_-)\right) \right) \right\} } \mathbb {1}_{\left\{ \theta _2 \le \tilde{m}^b\left( \alpha \textrm{H}^{i_1}\left( \nu ^\textrm{X}(s_-)\right) + (1-\alpha )\textrm{H}^{i_2}\left( \nu ^\textrm{X}(s_-)\right) \right) \right\} } \\&\quad \times \textrm{N}^{\textrm{b};p}(ds,di_1,di_2,d\alpha ,d\theta _1,d\theta _2)\,.\\&\quad \int _0^t \int _{\mathbb {N}_0^2} \int _{0}^1 \int _{\mathbb {R}_+^2} \left[ \left\langle f^\textrm{Y},\nu ^{\textrm{Y}}(s_-) + \delta _{\alpha \textrm{H}^{i_1}\left( \nu ^\textrm{X}(s_-)\right) + (1-\alpha )\textrm{H}^{i_2}\left( \nu ^\textrm{X}(s_-)\right) } \right\rangle - \left\langle f^\textrm{Y},\nu ^{\textrm{Y}}(s_-) \right\rangle \right] \\&\quad \times \mathbb {1}_{\left\{ i_1 < i_2 \le N^\textrm{X}(s_-)\right\} }\mathbb {1}_{\left\{ \theta _1 \le p\left( \textrm{H}^{i_1}\left( \nu ^\textrm{X}(s_-)\right) ,\textrm{H}^{i_2}\left( \nu ^\textrm{X}(s_-)\right) \right) \textrm{b}\left( \textrm{H}^{i_1}\left( \nu ^\textrm{X}(s_-)\right) ,\textrm{H}^{i_2}\left( \nu ^\textrm{X}(s_-)\right) , \left\langle \Gamma ^\textrm{b},\nu \right\rangle \right) \right\} } \mathbb {1}_{\left\{ \theta _2 \le m(q|\textrm{H}^{i_1}\left( \nu ^\textrm{X}(s_-)\right) ,\textrm{H}^{i_2}\left( \nu ^\textrm{X}(s_-)\right) )\right\} } \\&\quad \times \textrm{N}^{\textrm{b};p}(ds,di_1,di_2,dq,d\theta _1,d\theta _2) \end{aligned} \end{aligned}$$
(iii).3 If the sampling measure (13) is considered, then the integral terms with respect to the Poisson point measure $\textrm{N}^{\textrm{b};p}$ becomes
  $$\begin{aligned} \begin{aligned}&\int _0^t \int _{\mathbb {N}_0^2} \int _{\{1,\dots ,J\}} \int _{\mathbb {R}_+^2} \left[ \left\langle f^\textrm{Y},\nu ^{\textrm{Y}}(s_-) + \delta _{q} \right\rangle - \left\langle f^\textrm{Y},\nu ^{\textrm{Y}}(s_-) \right\rangle \right] \mathbb {1}_{\left\{ i_1< i_2 \le N^\textrm{X}(s_-)\right\} } \\&\quad \times \mathbb {1}_{\left\{ \theta _1 \le p\left( \textrm{H}^{i_1}\left( \nu ^\textrm{X}(s_-)\right) ,\textrm{H}^{i_2}\left( \nu ^\textrm{X}(s_-)\right) \right) \textrm{b}\left( \textrm{H}^{i_1}\left( \nu ^\textrm{X}(s_-)\right) ,\textrm{H}^{i_2}\left( \nu ^\textrm{X}(s_-)\right) \right) \right\} } \mathbb {1}_{\left\{ \theta _2 \le \tilde{m}^b\left( \alpha _j \textrm{H}^{i_1}\left( \nu ^\textrm{X}(s_-)\right) + (1-\alpha _j)\textrm{H}^{i_2}\left( \nu ^\textrm{X}(s_-)\right) \right) \right\} } \\&\quad \times \textrm{N}^{\textrm{b};p}(ds,di_1,di_2,d\alpha _j,d\theta _1,d\theta _2)\,.\\&\quad \int _0^t \int _{\mathbb {N}_0^2} \int _{\{1,\dots ,J\}} \int _{\mathbb {R}_+^2} \left[ \left\langle f^\textrm{Y},\nu ^{\textrm{Y}}(s_-) + \delta _{\alpha _j \textrm{H}^{i_1}\left( \nu ^\textrm{X}(s_-)\right) + (1-\alpha _j)\textrm{H}^{i_2}\left( \nu ^\textrm{X}(s_-)\right) } \right\rangle - \left\langle f^\textrm{Y},\nu ^{\textrm{Y}}(s_-) \right\rangle \right] \\&\quad \times \mathbb {1}_{\left\{ i_1 < i_2 \le N^\textrm{X}(s_-)\right\} }\mathbb {1}_{\left\{ \theta _1 \le p\left( \textrm{H}^{i_1}\left( \nu ^\textrm{X}(s_-)\right) ,\textrm{H}^{i_2}\left( \nu ^\textrm{X}(s_-)\right) \right) \textrm{b}\left( \textrm{H}^{i_1}\left( \nu ^\textrm{X}(s_-)\right) ,\textrm{H}^{i_2}\left( \nu ^\textrm{X}(s_-)\right) , \left\langle \Gamma ^\textrm{b},\nu \right\rangle \right) \right\} }\\&\quad \times \mathbb {1}_{\left\{ \theta _2 \le m(q|\textrm{H}^{i_1}\left( \nu ^\textrm{X}(s_-)\right) ,\textrm{H}^{i_2}\left( \nu ^\textrm{X}(s_-)\right) )\right\} } \textrm{N}^{\textrm{b};p}(ds,di_1,di_2,dq,d\theta _1,d\theta _2)\,. \end{aligned} \end{aligned}$$
  Above, to be coherent with the notation employed throughout the paper, we employed the notation
  $$\begin{aligned} \int _{\{1,\dots ,J\}}\cdot \,\,, \end{aligned}$$
  to denote the summation
  $$\begin{aligned} \sum _{j=1}^J\cdot \,\,. \end{aligned}$$

#### Definition 3.7.1

We say that ${\varvec{\nu }}(t) = (\nu ^\textrm{X}(t),\nu ^\textrm{Y}(t))$ as defined in Eqs. 9–10 is a *spatial radiation-induced DNA damage repair model* if ${\varvec{\nu }} = \left( {\varvec{\nu }}(t)\right) _{t \in \mathbb {R}_+}$ is $\left( \mathcal {F}_t\right) _{t \in \mathbb {R}_+}-$adapted and for any $f^\textrm{X}$ and $f^\textrm{Y}\in C^2_0(\textrm{Q})$ Eq. (19) holds $\mathbb {P}-$a.s.

The above process is characterized by the following infinitesimal generator (Kallenberg 1997, Chapter 12),

20 $$\begin{aligned} \mathcal {L}F_{(f^\textrm{X},f^\textrm{Y})}(\nu ) = \mathcal {L}_d F_{(f^\textrm{X},f^\textrm{Y})}(\mathbf {\nu }) + \sum _{h \in \{\textrm{r},\textrm{a},\textrm{b}\}} \mathcal {L}_{h} F_{(f^\textrm{X},f^\textrm{Y})}(\nu )\,, \end{aligned}$$

where ${\mathcal {L}_h} F_{(f^\textrm{X},f^\textrm{Y})}(\mathbf {\nu })$ is the infinitesimal generator of the reaction terms, whereas $\mathcal {L}_d F_{(f^\textrm{X},f^\textrm{Y})}(\nu )$ is the infinitesimal generator of the diffusive part of Eq. (19).

In particular, we have

21 $$\begin{aligned} \begin{aligned} \mathcal {L}_d^\textrm{X}f^\textrm{X}(q)&= \mu ^\textrm{X}\left( q\right) \cdot \nabla f^\textrm{X}\left( q\right) ds +\frac{1}{2} Tr\left[ \Sigma ^\textrm{X}\left( q\right) \text{ Hess } \,f^\textrm{X}\left( q\right) \right] \,,\\ {\mathcal {L}_d^\textrm{Y}} f^\textrm{Y}(q)&= \mu ^\textrm{Y}\left( q\right) \cdot \nabla f^\textrm{Y}\left( q\right) ds +\frac{1}{2} Tr\left[ \Sigma ^\textrm{Y}\left( q\right) \text{ Hess }\, f^\textrm{Y}\left( q\right) \right] \,. \end{aligned} \end{aligned}$$

It further holds that

22 $$\begin{aligned} \begin{aligned} \mathcal {L}_d F_{(f^\textrm{X},f^\textrm{Y})}(\nu )&= \left\langle \mathcal {L}_d^\textrm{X}f^\textrm{X},\nu ^\textrm{X} \right\rangle \frac{\partial }{\partial x} F \left( \left\langle f^\textrm{X},\nu \right\rangle , \left\langle f^\textrm{Y},\nu \right\rangle \right) \\&\quad + \left\langle \mathcal {L}_d^\textrm{Y}f^\textrm{Y},\nu ^\textrm{Y} \right\rangle \frac{\partial }{\partial y} F \left( \left\langle f^\textrm{X},\nu \right\rangle , \left\langle f^\textrm{Y},\nu \right\rangle \right) + \\&\quad + \left\langle \nabla ^T f^\textrm{X}\, \sigma ^\textrm{X}\,\nabla f^\textrm{X},\nu ^\textrm{X} \right\rangle \frac{\partial ^2}{\partial x^2} F \left( \left\langle f^\textrm{X},\nu \right\rangle , \left\langle f^\textrm{Y},\nu \right\rangle \right) \\&\quad + \left\langle \nabla ^T f^\textrm{Y}\, \sigma ^\textrm{Y}\,\nabla f^\textrm{Y},\nu ^\textrm{Y} \right\rangle \frac{\partial ^2}{\partial y^2} F \left( \left\langle f^\textrm{X},\nu \right\rangle , \left\langle f^\textrm{Y},\nu \right\rangle \right) \,. \end{aligned} \end{aligned}$$

Regarding the infinitesimal generator of the reaction terms, it holds

23 $$\begin{aligned} \begin{aligned}&\mathcal {L}_{\textrm{r}} F_{(f^\textrm{X},f^\textrm{Y})}(\nu )\\&\quad = \int _{\textrm{Q}} \textrm{r}(q,\nu )\left[ F_{(f^\textrm{X},f^\textrm{Y})}(\nu ^\textrm{X}- \delta _{q},\nu ^\textrm{Y}) - F_{(f^\textrm{X},f^\textrm{Y})}(\nu ) \right] \nu ^\textrm{X}(dq)\,,\\&\mathcal {L}_{\textrm{a}} F_{(f^\textrm{X},f^\textrm{Y})}(\nu )\\&\quad = \int _{\textrm{Q}} \int _{\textrm{Q}} \textrm{a}(q,\nu )\left[ F_{(f^\textrm{X},f^\textrm{Y})}((\nu ^\textrm{X}- \delta _{q},\nu ^\textrm{Y}+ \delta _{\bar{q}})) - F_{(f^\textrm{X},f^\textrm{Y})}(\nu ) \right] m^{\textrm{a}}(\bar{q}|q)d\bar{q}\nu ^\textrm{X}(dq)\,,\\&\mathcal {L}_{\textrm{b}} F_{(f^\textrm{X},f^\textrm{Y})}(\nu ) \\&\quad = \int _{\tilde{\textrm{Q}}^2} \int _{\textrm{Q}} \textrm{p}(q_1,q_2)\textrm{b}(q_1,q_2,\nu )\left[ F_{(f^\textrm{X},f^\textrm{Y})}((\nu ^\textrm{X}- \delta _{q_1} - \delta _{q_2},\nu ^\textrm{Y}+ \delta _{\bar{q}})) - F_{(f^\textrm{X},f^\textrm{Y})}(\nu ) \right] \\&\qquad \times m^{\textrm{b}}(\bar{q}|q_1,q_2)d\bar{q}\nu ^\textrm{X}(dq_1)\nu ^\textrm{X}(dq_2) + \\&\qquad +\int _{\tilde{\textrm{Q}}^2} \left( 1-\textrm{p}(q_1,q_2)\right) \textrm{b}(q_1,q_2,\nu )\left[ F_{(f^\textrm{X},f^\textrm{Y})}((\nu ^\textrm{X}- \delta _{q_1} - \delta _{q_2},\nu ^\textrm{Y})) - F_{(f^\textrm{X},f^\textrm{Y})}(\nu ) \right] \nu ^\textrm{X}(dq_1)\nu ^\textrm{X}(dq_2)\,, \end{aligned} \end{aligned}$$

where we have denoted by

$$\begin{aligned} \tilde{\textrm{Q}}^2 := \textrm{Q}^2 \setminus \left\{ (q_1,q_2) \,:\, q_1=q_2 \right\} \,. \end{aligned}$$

### Stepwise construction of the process

In the present Section, we provide a step-wise construction of the process. Such construction, besides being relevant from a theoretical point of view, is particularly important in implementing a simulation algorithm for the process defined in the previous section. Notice that, using assumptions 3.4, we have that the rates *a*, *b* and *r* are bounded in the uniform norm. Thus, between the occurrence times of the jump components, each lesion moves according to the diffusive generator $\mathcal {D}_X$ and $\mathcal {D}_y$.

1. starts with a random measure
  $$\begin{aligned} {\varvec{\nu }}_0 := \left( \nu ^\textrm{X}_0 , \nu ^\textrm{Y}_0 \right) := \left( \sum _{i=1}^{N^\textrm{X}_0} \delta _{\textrm{X}^i(0)},\sum _{i=1}^{N^\textrm{Y}_0} \delta _{\textrm{Y}^i(0)}\right) \,, \end{aligned}$$
  and set $t=\tau ^0 = 0$. The initial distribution $M_0$ will be treated explicitly and in detail in Sect. 2.1;
2. every jump reaction $h \in \{a,b,r\}$ has an exponential clock; set thus the random time of the first reaction happening
  $$\begin{aligned} \begin{aligned} \tau _h^1 := \inf \left\{ t > 0 \, : \, \int _0^t \bar{\textrm{h}}(\nu (s))ds \ge \mathcal {E}^1_h \right\} \,,\quad \bar{h}\in \left\{ \bar{\textrm{r}},\bar{\textrm{a}},\bar{\textrm{b}}\right\} \end{aligned} \end{aligned}$$
  with $\mathcal {E}^1_h$ is an exponential random variable with parameter 1. Also, we have defined
  $$\begin{aligned} \begin{aligned} \bar{\textrm{r}}(\nu (s))&:= \sum _{i=1}^{N^\textrm{X}(s)} \textrm{r}\left( \textrm{H}^i\left( \nu ^\textrm{X}(s)\right) , \left\langle \Gamma ^{\textrm{r}}_{\textrm{H}^i\left( \nu ^\textrm{X}(s)\right) },\nu \right\rangle \right) \,,\\ \bar{\textrm{a}}(\nu (s))&:= \sum _{i=1}^{N^\textrm{X}(s)} \textrm{a}\left( \textrm{H}^i\left( \nu ^\textrm{X}(s)\right) , \left\langle \Gamma ^{\textrm{a}}_{\textrm{H}^i\left( \nu ^\textrm{X}(s)\right) },\nu \right\rangle \right) \,,\\ \bar{\textrm{b}}(\nu (s))&:= \sum _{\begin{array}{c} i_1,i_2=1\\ i_1< i_2 \end{array}}^{N^\textrm{X}(s)} \textrm{b}\left( \textrm{H}^{i_1}\left( \nu ^\textrm{X}(s)\right) ,\textrm{H}^{i_2}\left( \nu ^\textrm{X}(s)\right) , \left\langle \Gamma ^{\textrm{b}}_{\textrm{H}^{i_1}\left( \nu ^\textrm{X}(s)\right) ,\textrm{H}^{i_2}\left( \nu ^\textrm{X}(s)\right) },\nu \right\rangle \right) \,.\\ \end{aligned} \end{aligned}$$
3. set $\tau ^1 := \min _{h \in \{a,b,r\}}\tau _h^1$ and consider $h^1 \in \{a,b,r\}$ the reaction that triggers the random time $\tau ^1_{h^1}$;
4. let any lesion move according to the diffusion and drift coefficients as described in Eq. (14), until time $T \wedge \tau _1$ is reached. If *T* is reached exit, otherwise go to the next step;
5. sample the lesions and positions of the lesions that triggered the reaction and, in case either $\textrm{a}$ or $\textrm{b}$ fired, sample the position *q* of the new lesion $\textrm{Y}$ created;
6. at time $\tau ^1$, if:
  6.i $\textrm{r}$ has been triggered, set $N^\textrm{X}(\tau ^1) = N^\textrm{X}(\tau ^1_-) -1$ and $N^\textrm{Y}(\tau ^1) = N^\textrm{Y}(\tau ^1_-)$ and remove the $i-$th component of $\textrm{X}$, that is we have
    $$\begin{aligned} (\textrm{X}^1(\tau ^1),\dots ,\textrm{X}^{i-1}(\tau ^1),\textrm{X}^{i+1}(\tau ^1),\dots ,\textrm{X}^{N^X}(\tau ^1))\,; \end{aligned}$$
  6.ii $\textrm{a}$ has been triggered, set $N^\textrm{X}(\tau ^1) = N^\textrm{X}(\tau ^1_-) - 1$ and $N^\textrm{Y}(\tau ^1) = N^\textrm{Y}(\tau ^1_-)+1$, remove the $i-$th component of $\textrm{X}$ and create a new lesion $\textrm{Y}$ at $q \in \textrm{Q}$ sampled according to $m^\textrm{a}$. We thus have
    $$\begin{aligned} \begin{aligned}&(\textrm{X}^1(\tau ^1),\dots ,\textrm{X}^{i-1}(\tau ^1),\textrm{X}^{i+1}(\tau ^1),\dots ,\textrm{X}^{N^\textrm{X}}(\tau ^1))\,,\\&(\textrm{Y}^1(\tau ^1),\dots ,\textrm{Y}^{N^\textrm{Y}}(\tau ^1_-),\textrm{Y}^{N^\textrm{Y}+1}(\tau ^1))\,. \end{aligned} \end{aligned}$$
  6.iii $\textrm{b}$ has been triggered, and simulate a number $\tilde{p}$ from a random variable $P \sim U(0,1)$, if:
    6.iii.a $\tilde{p} \le \textrm{p}\left( \textrm{X}^{i_1}(\tau ^1),\textrm{X}^{i_2}(\tau ^1)\right)$, then set $N^\textrm{X}(\tau ^1) = N^\textrm{X}(\tau ^1_-) - 2$ and $N^\textrm{Y}(\tau ^1) = N^\textrm{Y}(\tau ^1_-)+1$, remove the $i_1-$th and $i_2-$th component of $\textrm{X}$ and create a new lesion $\textrm{Y}$ in position $q \in \textrm{Q}$ sampled according to $m^\textrm{b}$. We thus have
      $$\begin{aligned} \begin{aligned}&(\textrm{X}^1(\tau ^1),\dots ,\textrm{X}^{i_1-1}(\tau ^1),\textrm{X}^{i_1+1}(\tau ^1),\dots ,\textrm{X}^{i_2-1}(\tau ^1),\textrm{X}^{i_2+1}(\tau ^1),\dots ,\textrm{X}^{N^\textrm{X}}(\tau ^1))\,,\\&(\textrm{Y}^1(\tau ^1),\dots ,\textrm{Y}^{N^\textrm{Y}}(\tau ^1_-),\textrm{Y}^{N^\textrm{Y}+1}(\tau ^1))\,. \end{aligned} \end{aligned}$$
    6.iii.b $\tilde{p} > \textrm{p}\left( \textrm{X}^{i_1}(\tau ^1),\textrm{X}^{i_2}(\tau ^1)\right)$, then set $N^\textrm{X}(\tau ^1) = N^\textrm{X}(\tau ^1_-) - 2$ and $N^\textrm{Y}(\tau ^1) = N^\textrm{Y}(\tau ^1_-)$, remove the $i_1-$th and $i_2-$th component of $\textrm{X}$ remove lesions $i_1$ and $i_2$ from the system. We thus have
      $$\begin{aligned} \begin{aligned}&(\textrm{X}^1(\tau ^1),\dots ,\textrm{X}^{i_1-1}(\tau ^1),\textrm{X}^{i_1+1}(\tau ^1),\dots ,\textrm{X}^{i_2-1}(\tau ^1),\textrm{X}^{i_2+1}(\tau ^1),\dots ,\textrm{X}^{N^\textrm{X}}(\tau ^1))\,,\\&(\textrm{Y}^1(\tau ^1),\dots ,\textrm{Y}^{N^\textrm{Y}}(\tau ^1_-))\,. \end{aligned} \end{aligned}$$
7. update $$t = t + \tau ^1$$;
8. if $t<T$, go to step 1 and repeat until *T* is reached.

### Well-posedness and martingale properties

In the present Section, we prove the existence and uniqueness of solutions to the above-introduced model.

#### Theorem 3.8

Let $\nu ^\textrm{X}_0$ and $\nu ^\textrm{Y}_0$ two independent random measures with finite $p-$th moment, $p \ge 1$, that is it holds

24 $$\begin{aligned} \mathbb {E} \left\langle \textbf{1},\nu ^\textrm{X}_0 \right\rangle ^p< \infty \,,\quad \mathbb {E} \left\langle \textbf{1},\nu ^\textrm{Y}_0 \right\rangle ^p < \infty \,. \end{aligned}$$

Then, under Hypothesis 3.4, for any $T>0$, there exists a pathwise unique strong solution to the system (19) in $\mathcal {D}\left( [0,T],\mathcal {M}\times \mathcal {M}\right)$. Also, it holds

25 $$\begin{aligned} \mathbb {E} \sup _{t \le T} \left\langle \textbf{1},\nu (t) \right\rangle ^p < \infty \,. \end{aligned}$$

In particular, the process $\nu$ in Definition 3.7.1 is well-defined on $\mathbb {R}_+$.

#### Proof

Since the jump times are isolated, [Kallenberg (1997), Chapter 12], the construction of $\nu ^\textrm{X}(t)$ and $\nu ^\textrm{Y}(t)$ can be done pathwise inductively along the successive jump times. In particular, denote by $T_{m}$ and $T_{m+1}$ two successive jump times of the process $\nu$, and assume that at time $T_m$ we have $N^\textrm{X}(T_m)$, resp. $N^\textrm{Y}(T_m)$, lesions of type $\textrm{X}$, resp. $\textrm{Y}$. As noted above, for $t \in [T_{m},T_{m+1})$ the number of lesions remains constant so that the process is solely subject to the diffusive component as described in Eq. (14). Using Hypothesis 3.4, conditionally on $\mathcal {F}_{T_m}$, Eq. (14) can be seen as a purely diffusive SDE with globally Lipschitz coefficients on $\mathbb {R}^{d \times N^\textrm{X}(T_m)} \times \mathbb {R}^{d \times N^\textrm{Y}(T_m)}$, so that the process

$$\begin{aligned} \left( \textrm{X}^{i_\textrm{X}}(t),\textrm{Y}^{i_\textrm{Y}}(t) \right) _{i_\textrm{X}=1,\dots ,N^\textrm{X}(T_n);\, i_\textrm{Y}=1,\dots ,N^\textrm{Y}(T_n)}\,, \end{aligned}$$

admits a unique strong solution for $t \in [T_{m},T_{m+1})$.

Define then, for $n \ge 0$,

$$\begin{aligned} \tau _n^\textrm{X}:= \inf \{t \ge 0 \,:\, \left\langle \textbf{1},\nu ^\textrm{X}(t) \right\rangle \ge n\}\,, \end{aligned}$$

and set for short $\bar{\tau }_n^\textrm{X}:= t \wedge \tau _n^\textrm{X}$.

We can construct a solution algorithmically in [0, *T*). For $t \ge 0$, noticing that the number of lesions in $\textrm{X}$ can only decrease, we have,

26 $$\begin{aligned} \begin{aligned} \sup _{s \in [0,\bar{\tau }_n^\textrm{X}]} \left\langle \textbf{1},\nu ^\textrm{X}(s) \right\rangle ^p&\le \left\langle \textbf{1},\nu ^\textrm{X}_0 \right\rangle ^p \,. \end{aligned} \end{aligned}$$

Regarding $\textrm{Y}$, using Itô formula and taking the supremum over the interval $[0,\bar{\tau }_n^\textrm{Y}]$, we have that,

27 $$\begin{aligned} \begin{aligned}&\sup _{s \in [0,\bar{\tau }_n^\textrm{Y}]} \left\langle \textbf{1},\nu ^\textrm{Y}(s) \right\rangle ^p \le \left\langle \textbf{1},\nu ^\textrm{Y}_0 \right\rangle ^p\\&+\int _0^{\bar{\tau }_n^\textrm{Y}} \int _{\mathbb {N}_0} \int _{\textrm{Q}} \int _{\mathbb {R}_+^2} \left[ \left( \left\langle \textbf{1},\nu ^{\textrm{Y}}(s_-) \right\rangle + 1\right) ^p - \left\langle \textbf{1},\nu ^{\textrm{Y}}(s_-) \right\rangle ^p\right] \\&\times \mathbb {1}_{\left\{ i \le N^\textrm{X}(s_-)\right\} }\mathbb {1}_{\left\{ \theta _1 \le \textrm{a}\left( \textrm{H}^i\left( \nu ^\textrm{X}(s_-)\right) , \left\langle \Gamma ^\textrm{a},\nu \right\rangle \right) \right\} } \mathbb {1}_{\left\{ \theta _2 \le m^\textrm{a}\left( q|\textrm{H}^i\left( \nu ^\textrm{X}(s_-)\right) \right) \right\} } \textrm{N}^\textrm{a}(ds,di,dq,d\theta _1,d\theta _2)\\&+ \int _0^{\bar{\tau }_n^\textrm{Y}} \int _{\mathbb {N}_0^2} \int _{\textrm{Q}} \int _{\mathbb {R}_+^2} \left[ \left( \left\langle \textbf{1},\nu ^{\textrm{Y}}(s_-) \right\rangle + 1\right) ^p - \left\langle \textbf{1},\nu ^{\textrm{Y}}(s_-) \right\rangle ^p\right] \\&\times \mathbb {1}_{\left\{ i_1 < i_2 \le N^\textrm{X}(s_-)\right\} }\mathbb {1}_{\left\{ \theta _1 \le \textrm{b}\left( \textrm{H}^{i_1}\left( \nu ^\textrm{X}(s_-)\right) ,\textrm{H}^{i_2}\left( \nu ^\textrm{X}(s_-)\right) , \left\langle \Gamma ^\textrm{b},\nu \right\rangle \right) \right\} } \mathbb {1}_{\left\{ \theta _2 \le m^\textrm{b}(q|\textrm{H}^{i_1}\left( \nu ^\textrm{X}(s_-)\right) ,\textrm{H}^{i_2}\left( \nu ^\textrm{X}(s_-)\right) )\right\} } \textrm{N}^{\textrm{b};p}(ds,di_1,di_2,dq,d\theta _1,d\theta _2)\,. \end{aligned} \end{aligned}$$

Taking the expectation in Eq. (27), using estimate (26) and Hypothesis 3.4, together with

$$\begin{aligned} (1+y)^p - y^p \le C(1+y^{p-1})\,,\quad \forall \, y \ge 0\,. \end{aligned}$$

we have that, for some $C>0$ that can take possibly different values,

28 $$\begin{aligned}{} & {} \mathbb {E}\sup _{s \in [0,\bar{\tau }_n^\textrm{Y}]} \left\langle \textbf{1},\nu ^\textrm{Y}(s) \right\rangle ^p \le \mathbb {E} \left\langle \textbf{1},\nu ^\textrm{Y}_0 \right\rangle ^p \nonumber \\{} & {} \qquad + \mathbb {E}\int _0^{\bar{\tau }_n^\textrm{Y}} \int _{\mathbb {N}_0} \int _{\textrm{Q}} \int _{\mathbb {R}_+^2} \left[ \left( \left\langle \textbf{1},\nu ^{\textrm{Y}}(s_-) \right\rangle + 1\right) ^p - \left\langle \textbf{1},\nu ^{\textrm{Y}}(s_-) \right\rangle ^p\right] \nonumber \\{} & {} \qquad \times \mathbb {1}_{\left\{ i \le N^\textrm{X}(s_-)\right\} }\mathbb {1}_{\left\{ \theta _1 \le \textrm{a}\left( \textrm{H}^i\left( \nu ^\textrm{X}(s_-)\right) , \left\langle \Gamma ^\textrm{a},\nu \right\rangle \right) \right\} } \mathbb {1}_{\left\{ \theta _2 \le m^\textrm{a}\left( q|\textrm{H}^i\left( \nu ^\textrm{X}(s_-)\right) \right) \right\} } \textrm{N}^\textrm{a}(ds,di,dq,d\theta _1,d\theta _2)\nonumber \\{} & {} \qquad + \mathbb {E}\int _0^{\bar{\tau }_n^\textrm{Y}} \int _{\mathbb {N}_0^2} \int _{\textrm{Q}} \int _{\mathbb {R}_+^2} \left[ \left( \left\langle \textbf{1},\nu ^{\textrm{Y}}(s_-) \right\rangle + 1\right) ^p - \left\langle \textbf{1},\nu ^{\textrm{Y}}(s_-) \right\rangle ^p\right] \nonumber \\{} & {} \qquad \times \mathbb {1}_{\left\{ i_1 < i_2 \le N^\textrm{X}(s_-)\right\} }\mathbb {1}_{\left\{ \theta _1 \le \textrm{b}\left( \textrm{H}^{i_1}\left( \nu ^\textrm{X}(s_-)\right) ,\textrm{H}^{i_2}\left( \nu ^\textrm{X}(s_-)\right) , \left\langle \Gamma ^\textrm{b},\nu \right\rangle \right) \right\} }\nonumber \\{} & {} \qquad \mathbb {1}_{\left\{ \theta _2 \le m^\textrm{b}(q|\textrm{H}^{i_1}\left( \nu ^\textrm{X}(s_-)\right) ,\textrm{H}^{i_2}\left( \nu ^\textrm{X}(s_-)\right) )\right\} } \textrm{N}^{\textrm{b};p}(ds,di_1,di_2,dq,d\theta _1,d\theta _2) \le \nonumber \\{} & {} \quad \le \mathbb {E} \left\langle \textbf{1},\nu ^\textrm{Y}_0 \right\rangle ^p + \mathbb {E}\int _0^{\bar{\tau }_n^\textrm{Y}} \sum _{i = 1}^{ \left\langle \textbf{1},\nu ^\textrm{X}(s) \right\rangle } \int _{\textrm{Q}} \left[ \left( \left\langle \textbf{1},\nu ^{\textrm{Y}}(s_-) \right\rangle + 1\right) ^p - \left\langle \textbf{1},\nu ^{\textrm{Y}}(s_-) \right\rangle ^p\right] \nonumber \\{} & {} \qquad \times \textrm{a}\left( \textrm{H}^i\left( \nu ^\textrm{X}(s_-)\right) , \left\langle \Gamma ^\textrm{a},\nu \right\rangle \right) m^\textrm{a}\left( q|\textrm{H}^i\left( \nu ^\textrm{X}(s_-)\right) \right) ds\nonumber \\{} & {} \qquad + \mathbb {E}\int _0^{\bar{\tau }_n^\textrm{Y}} \sum _{\begin{array}{c} i_1\,,\,i_2 = 1,\\ i_1 \le i_2 \end{array}}^{ \left\langle \textbf{1},\nu ^\textrm{X}(s) \right\rangle } \int _{\textrm{Q}} \left[ \left( \left\langle \textbf{1},\nu ^{\textrm{Y}}(s_-) \right\rangle + 1\right) ^p - \left\langle \textbf{1},\nu ^{\textrm{Y}}(s_-) \right\rangle ^p\right] \nonumber \\{} & {} \qquad \times \textrm{b}\left( \textrm{H}^{i_1}\left( \nu ^\textrm{X}(s_-)\right) ,\textrm{H}^{i_2}\left( \nu ^\textrm{X}(s_-)\right) , \left\langle \Gamma ^\textrm{a},\nu \right\rangle \right) m^\textrm{b}(q|\textrm{H}^{i_1}\left( \nu ^\textrm{X}(s_-)\right) ,\textrm{H}^{i_2}\left( \nu ^\textrm{X}(s_-)\right) ) ds \le \nonumber \\{} & {} \quad \le \mathbb {E} \left\langle \textbf{1},\nu ^\textrm{Y}_0 \right\rangle ^p +C \, \bar{\textrm{a}}\, \bar{\Gamma }^\textrm{a}\, \mathbb {E} \left\langle \textbf{1},\nu ^{\textrm{X}}_0 \right\rangle \mathbb {E}\int _0^{\bar{\tau }_n^\textrm{Y}} \left( 1+ \left\langle \textbf{1},\nu ^{\textrm{Y}}(s_-) \right\rangle ^{p-1} \right) \left\langle \textbf{1},\nu ^{\textrm{Y}}(s_-) \right\rangle ds \nonumber \\{} & {} \qquad + C \, \bar{\textrm{b}}\, \bar{\Gamma }^\textrm{b}\, \mathbb {E} \left\langle \textbf{1},\nu ^{\textrm{X}}_0 \right\rangle ^2\mathbb {E}\int _0^{\bar{\tau }_n^\textrm{Y}} \left( 1+ \left\langle \textbf{1},\nu ^{\textrm{Y}}(s_-) \right\rangle ^{p-1} \right) \left\langle \textbf{1},\nu ^{\textrm{Y}}(s_-) \right\rangle ds \le \nonumber \\{} & {} \quad \le C \left( 1+ \mathbb {E}\int _0^t \left\langle \textbf{1},\nu ^{\textrm{Y}}(s \wedge \tau _n^\textrm{Y}) \right\rangle ^p \right) \,. \end{aligned}$$

From Gronwall lemma it thus follows that it exists $C>0$ depending on *p* and *T* but independent of *n*, such that

29 $$\begin{aligned} \mathbb {E}\sup _{s \in [0,\bar{\tau }_n^\textrm{Y}]} \left\langle \textbf{1},\nu ^\textrm{Y}(s) \right\rangle ^p \le C\,. \end{aligned}$$

Letting thus $n \rightarrow \infty$, we have that

30 $$\begin{aligned} \tau _n^\textrm{Y}\rightarrow \infty \quad \text{ a.s. }\,. \end{aligned}$$

In fact, if that was not the case, we can find $T_0<\infty$ such that

$$\begin{aligned} \mathbb {P}\left( \sup _n \tau _n^\textrm{Y}< T_0\right) = \varepsilon (T_0) >0\,. \end{aligned}$$

This would in turn yields

$$\begin{aligned} \mathbb {E}\sup _{s \in [0,T_0 \wedge {\tau _n^\textrm{Y}}]} \left\langle \textbf{1},\nu ^\textrm{Y}(s) \right\rangle ^p \ge \varepsilon (T_0) n^p\,, \end{aligned}$$

which contradicts Eq. (29). A similar argument holds for $\textrm{X}$. Using Fatou’s lemma we can let $n \rightarrow \infty$ as

$$\begin{aligned} \mathbb {E}\lim \inf _{n \rightarrow \infty } \sup _{s \in [0,T \wedge \tau _n^\textrm{X}]} \left\langle \textbf{1},\nu ^\textrm{Y}(s) \right\rangle ^p \le \lim \inf _{n \rightarrow \infty } \mathbb {E}\sup _{s \in [0,T \wedge \tau _n^\textrm{X}]} \left\langle \textbf{1},\nu ^\textrm{Y}(s) \right\rangle ^p \le C < \infty \,, \end{aligned}$$

proving thus (25).

At last, since the above claim holds also for $p=1$, we have that

31 $$\begin{aligned} \mathbb {E} \sup _{t \le T} \left\langle \textbf{1},\nu (t) \right\rangle < \infty \,, \end{aligned}$$

so that the process $\nu$ can be constructed step by step between consecutive jumps and the sequence of jump times $\left( T_m\right) _{m \in \mathbb {N}}$ goes to infinity and the process in well-defined. The proof is thus complete. $\square$

#### Remark 3.9

Notice that if, instead of conditions $(2.ii)-(2.iii)$ in Hypothesis 3.4 we require the weaker conditions

$$\begin{aligned} \begin{aligned} \sup _{q \in \textrm{Q}} \sup _{v \in [0,N]} \textrm{a}(q,v)< \bar{\textrm{a}}< \infty \,,\\ \sup _{q_1,q_2 \in \textrm{Q}} \sup _{v \in [0,N]} \textrm{b}(q_1,q_2,v)< \bar{\textrm{b}} < \infty \,,\\ \end{aligned} \end{aligned}$$

Theorem 3.8 would follow analogously with the only difference that existence and uniqueness can be proved only up to a sufficiently small finite horizon time $T_0<\infty$ rather than on the whole real line $\mathbb {R}_+$. In particular, Eq. (30) does not hold. To see that, consider the truncated *death rate* and *pairwise interaction rate*

$$\begin{aligned} \textrm{a}_n(q,v):= \textrm{a}(q,v) \mathbb {1}_{\left\{ v \le n\right\} }\,,\quad \textrm{b}_n(q,v):= \textrm{b}(q,v) \mathbb {1}_{\left\{ v \le n\right\} }\,. \end{aligned}$$

By the boundedness of the rates $\textrm{a}_n$ and $\textrm{b}_n$, we have that Theorem 3.8 is valid and existence and uniqueness hold true up to a stopping time $\tau _n$. We further clearly have that $\tau _n \le \tau _{n+1}$, so that, if $\tau _n \rightarrow \infty$ as $n \rightarrow \infty$, we have the existence and uniqueness for any time horizon *T*, whereas if on the contrary we have that $\tau _n \rightarrow T_0$, we have an explosion of the solution in finite time. $\triangle$

The next result states a martingale property for the spatial GSM$^2$introduced in previous sections.

#### Theorem 3.10

Assume that Hypothesis 3.4 holds true and that $\nu ^\textrm{X}_0$ and $\nu ^\textrm{Y}_0$ are two random measures independent with finite $p-$th moment, $p \ge 2$. Then:

(i) ${\varvec{\nu }}$ is a Markov process with infinitesimal generator $\mathcal {L}$ defined by (20);
(ii) assume that for $F \in C^2_b (\mathbb {R}\times \mathbb {R})$ and for $f^\textrm{X}\,,\,f^\textrm{Y}\in {\mathcal {C}^{2}_0}(\textrm{Q})$ such that for all ${\varvec{\nu }} \in \mathcal {M}\times \mathcal {M}$, it holds
  32 $$\begin{aligned} |F_{(f^\textrm{X},f^\textrm{Y})}({\varvec{\nu }})| + |\mathcal {L}F_{(f^\textrm{X},f^\textrm{Y})}({\varvec{\nu }})| \le C \left( 1+ \left\langle \textbf{1},{\varvec{\nu }}_0 \right\rangle ^p \right) \,. \end{aligned}$$
  Then, the process
  33 $$\begin{aligned} F_{(f^\textrm{X},f^\textrm{Y})}({\varvec{\nu }}(t)) - F_{(f^\textrm{X},f^\textrm{Y})}({\varvec{\nu }}_0) - \int _0^t \mathcal {L}F_{(f^\textrm{X},f^\textrm{Y})}({\varvec{\nu }}(s))ds\,, \end{aligned}$$
  is a cádlág martingale starting at 0;
(iii) the processes $\textrm{M}^\textrm{X}$ and $\textrm{M}^\textrm{Y}$ defined for $f^\textrm{X}\,,\,f^\textrm{Y}\in C^2_0$ by
  34 $$\begin{aligned} {\left\{ \begin{array}{ll} \textrm{M}^\textrm{X}(t) &{}= \left\langle f^\textrm{X},\nu ^\textrm{X}(t) \right\rangle - \left\langle f^\textrm{X},\nu ^\textrm{X}_0 \right\rangle - \int _0^t \left\langle \mathcal {L}^\textrm{X}_d f^\textrm{X}(x),\nu ^\textrm{X}(s) \right\rangle ds \\ &{}\quad + \int _0^t\int _{\textrm{Q}} \left[ \textrm{r}(q,\nu ) + \textrm{a}(q,\nu ) \right] f^\textrm{X}(q)\nu ^\textrm{X}(s)(dq)ds\\ &{}\quad +\int _0^t\int _{\tilde{\textrm{Q}}^2} \textrm{b}(q_1,q_2,\nu ) (f^\textrm{X}(q_1)+f^\textrm{X}(q_2))\nu ^\textrm{X}(s)(dq_1)\nu ^\textrm{X}(s)(dq_2)ds\\ \textrm{M}^\textrm{Y}(t) &{}= \left\langle f^\textrm{Y},\nu ^\textrm{Y}(t) \right\rangle - \left\langle f^\textrm{Y},\nu ^\textrm{Y} \right\rangle - \int _0^t \left\langle \mathcal {L}^\textrm{Y}_d f^\textrm{Y}(x),\nu ^\textrm{Y}(s) \right\rangle ds \\ &{}\quad - \int _0^t \int _{\textrm{Q}} \textrm{a}(q,\nu ) \int _{\textrm{Q}} m^{\textrm{a}}(\bar{q}|q) f^\textrm{Y}(\bar{q})d\bar{q} \nu ^\textrm{X}(s)(dq)ds\\ &{}\quad - \int _0^t\int _{\tilde{\textrm{Q}}^2} \int _{\textrm{Q}} \textrm{p}(q_1,q_2) \textrm{b}(q_1,q_2,\nu ) f^\textrm{Y}(\bar{q}) m^{\textrm{b}}(\bar{q}|q_1,q_2)d\bar{q}\nu ^\textrm{X}(s)(dq_1)\nu ^\textrm{X}(s)(dq_2)ds\,, \end{array}\right. } \end{aligned}$$
  are cádlág $L^2-$martingale starting at 0 with predictable quadratic variation given by
  35 $$\begin{aligned} {\left\{ \begin{array}{ll} \left\langle \textrm{M}^\textrm{X} \right\rangle (t) &{}= \int _0^t \left\langle \nabla ^T f^\textrm{X}\sigma ^\textrm{X}\nabla f^\textrm{X}, \nu ^\textrm{X} \right\rangle ds \\ &{}\quad + \int _0^t \int _{\textrm{Q}} \left[ \textrm{r}(q,\nu (s)) + \textrm{a}(q,\nu ) \right] \left( f^\textrm{X}(q) \right) ^2 \nu ^\textrm{X}(s)(dq)ds\\ &{}\quad +\int _0^t\int _{\tilde{\textrm{Q}}^2} \textrm{b}(q_1,q_2,\nu ) (f^\textrm{X}(q_1)+f^\textrm{X}(q_2))^2 \nu ^\textrm{X}(s)(dq_1)\nu ^\textrm{X}(s)(dq_2)ds\\ \left\langle \textrm{M}^\textrm{Y} \right\rangle (t) &{}= \int _0^t \left\langle \nabla ^T f^\textrm{Y}\sigma ^\textrm{Y}\nabla f^\textrm{Y}, \nu ^\textrm{Y} \right\rangle ds \\ &{}\quad - \int _0^t \int _{\textrm{Q}} \textrm{a}(q,\nu ) \int _{\textrm{Q}} m^{\textrm{a}}(\bar{q}|q) \left( f^\textrm{Y}(\bar{q})\right) ^2 d\bar{q} \nu ^\textrm{X}(s)(dq)ds\\ &{}\quad - \int _0^t\int _{\tilde{\textrm{Q}}^2} \int _{\textrm{Q}} \textrm{p}(q_1,q_2) \textrm{b}(q_1,q_2,\nu ) \left( f^\textrm{Y}(\bar{q})\right) ^2 m^{\textrm{b}}(\bar{q}|q_1,q_2)d\bar{q}\nu ^\textrm{X}(s)(dq_1)\nu ^\textrm{X}(s)(dq_2)ds\,. \end{array}\right. } \end{aligned}$$

#### Proof

(i) to show that $\nu$ is a Markov process is standard using the fact Poisson point processes have independent increments. Then, for any function $f^\textrm{X}$ and $f^\textrm{Y}\in C^2_0(\textrm{Q})$, we have the representation given in Eq. (19). By compensation, we can reformulate Eq. (19) as
  36 $$\begin{aligned} {\left\{ \begin{array}{ll} \left\langle f^\textrm{X},\nu ^\textrm{X}(t) \right\rangle &{}= \left\langle f^\textrm{X},\nu ^\textrm{X}(0) \right\rangle + \int _0^t \left\langle \mathcal {L}^\textrm{X}_d f^\textrm{X},\nu ^\textrm{X}(s) \right\rangle ds \\ &{}+ \int _0^t \left\langle \left[ \textrm{r}(\cdot ,\nu ) + \textrm{a}(\cdot ,\nu ) \right] f^\textrm{X},\nu ^\textrm{X}(s) \right\rangle ds\\ &{}+\int _0^t \left\langle \left\langle \textrm{b}(\cdot ,\cdot ,\nu (s)) (f^\textrm{X}+f^\textrm{X}),\nu ^\textrm{X}(s) \right\rangle ,\nu ^\textrm{X}(s) \right\rangle ds \\ \left\langle f^\textrm{Y},\nu ^\textrm{Y}(t) \right\rangle &{}= \left\langle f^\textrm{Y},\nu ^\textrm{Y}(0) \right\rangle + \int _0^t \left\langle \mathcal {L}^\textrm{Y}_d f^\textrm{Y},\nu ^\textrm{Y} \right\rangle ds \\ &{}- \int _0^t \left\langle \textrm{a}(\cdot ,\nu ) \int _{\textrm{Q}} m^{\textrm{a}}(\bar{q}|\cdot ) f^\textrm{Y}(\bar{q})d\bar{q}, \nu ^\textrm{X}(s) \right\rangle ds\\ &{}- \int _0^t \left\langle \left\langle \int _{\textrm{Q}} \textrm{p}(\cdot ,\cdot )\textrm{b}(\cdot ,\cdot ) f^\textrm{Y}(\bar{q}) m^{\textrm{b}}(\bar{q}|\cdot ,\cdot )d\bar{q},\nu ^\textrm{X}(s) \right\rangle ,\nu ^\textrm{X}(s) \right\rangle ds + \tilde{\mathcal {M}}^\textrm{Y}(t) \,, \end{array}\right. } \end{aligned}$$
  where $\tilde{\mathcal {M}}^\textrm{X}$ and $\tilde{\mathcal {M}}^\textrm{Y}$ are local-martingales accounting for the noises *W*, $\textrm{N}^\textrm{r}$, $\textrm{N}^\textrm{a}$ and $\textrm{N}^\textrm{b}$. A straightforward computation shows that for $F \in C^2_b (\mathbb {R}\times \mathbb {R})$, dividing Eq. (36) by *t*, taking the limit as $t \downarrow 0$ and taking the expectation we finally have that $\mathcal {L}F_{(f^\textrm{X},f^\textrm{Y})}(\nu )$ has the expression as given in Eq. (20).
(ii) using condition (32) we have can infer that (33) is integrable and well-defined. Using point (i) we can finally conclude that (33) is a cádlág martingale.
(iii) notice first that point (ii) holds true for any $F_f(\nu ) = \left\langle f,\nu \right\rangle ^q$, $q \in \{1,\dots ,p-1\}$, since by Eq. (25) it follows the estimate (32). Therefore, choosing $q=1$ we immediately have that $\textrm{M}^\textrm{X}(t)$ and $\textrm{M}^\textrm{Y}(t)$ are martingales. Using $p=2$ we obtain computing $F_f(\nu ) = \left\langle f,\nu \right\rangle ^2$ from equations (22)-(23),
  37 $$\begin{aligned} {\left\{ \begin{array}{ll} &{} \left\langle f^\textrm{X},\nu ^\textrm{X}(t) \right\rangle ^2 - \left\langle f^\textrm{X},\nu ^\textrm{X}(0) \right\rangle ^2 \\ &{} - \int _0^t \int _\textrm{Q}\left[ 2 \left\langle f^\textrm{X},\nu ^\textrm{X}(s) \right\rangle \left( \mu ^\textrm{X}\left( q, \left\langle \Gamma ^{\mu ,\textrm{X}}_{q},\nu \right\rangle \right) \cdot \nabla f^\textrm{X}\left( q\right) ds + \frac{1}{2} Tr\left[ \Sigma ^\textrm{X}\left( q, \left\langle \Gamma ^{\sigma ,\textrm{X}}_{q},\nu \right\rangle \right) \text{ Hess } \,f^\textrm{X}\left( q\right) \right] \right) \right] \nu ^\textrm{X}(s)(dq)ds\\ &{}-\int _0^t \int _\textrm{Q}2 \nabla ^T f^\textrm{X}(q)\, \sigma ^\textrm{X}\,\nabla f^\textrm{X}(q) \nu ^\textrm{X}(s)(dq)ds\\ &{}+\int _0^t \int _\textrm{Q}\textrm{r}(q,\nu )\left[ \left( f^\textrm{X}(q)\right) ^2 - 2 f^\textrm{X}(q) \left\langle f^\textrm{X},\nu ^\textrm{X}(s) \right\rangle \right] \nu ^\textrm{X}(s)(dq)ds\\ &{}+ \int _0^t \int _{\textrm{Q}} \textrm{a}(q,\nu )\left[ \left( f^\textrm{X}(q)\right) ^2 - 2 f^\textrm{X}(q) \left\langle f^\textrm{X},\nu ^\textrm{X}(s) \right\rangle \right] \nu ^\textrm{X}(s)(dq)ds\\ &{}+ \int _0^t \int _{\tilde{\textrm{Q}}^2}\left[ \left( f^\textrm{X}(q_1)\right) ^2 + \left( f^\textrm{X}(q_1)\right) ^2 +f^\textrm{X}(q_1)f^\textrm{X}(q_2) - 2 f^\textrm{X}(q_1) \left\langle f^\textrm{X},\nu ^\textrm{X}(s) \right\rangle - 2 f^\textrm{X}(q_2) \left\langle f^\textrm{X},\nu ^\textrm{X}(s) \right\rangle \right] \\ &{}\qquad \qquad \qquad \qquad \times \textrm{b}(q_1,q_2,\nu ) \nu ^\textrm{X}(s)(dq_1)\nu ^\textrm{X}(s)(dq_2) \,ds\\ &{} \left\langle f^\textrm{Y},\nu ^\textrm{Y}(t) \right\rangle ^2 - \left\langle f^\textrm{Y},\nu ^\textrm{Y}(0) \right\rangle ^2 \\ &{} - \int _0^t \int _\textrm{Q}\left[ 2 \left\langle f^\textrm{Y},\nu ^\textrm{Y}(s) \right\rangle \left( \mu ^\textrm{Y}\left( q, \left\langle \Gamma ^{\mu ,\textrm{Y}}_{q},\nu \right\rangle \right) \cdot \nabla f^\textrm{Y}\left( q\right) ds + \frac{1}{2} Tr\left[ \Sigma ^\textrm{Y}\left( q, \left\langle \Gamma ^{\sigma ,\textrm{Y}}_{q},\nu \right\rangle \right) \text{ Hess } \,f^\textrm{Y}\left( q\right) \right] \right) \right] \nu ^\textrm{Y}(s)(dq)ds\\ &{}-\int _0^t \int _\textrm{Q}2 \nabla ^T f^\textrm{Y}(q)\, \sigma ^\textrm{Y}\,\nabla f^\textrm{Y}(q) \nu ^\textrm{Y}(s)(dq)ds\\ &{}-\int _0^t \int _{\textrm{Q}} \int _{\textrm{Q}} \textrm{a}(q,\nu )\left[ \left( f^\textrm{Y}(\bar{q})\right) ^2 + 2 f^\textrm{Y}(\bar{q}) \left\langle f^\textrm{Y},\nu ^\textrm{Y}(s) \right\rangle \right] m^{\textrm{a}}(\bar{q}|q)d\bar{q}\nu ^\textrm{X}(s)(dq)ds\\ &{}-\int _0^t \int _{\tilde{\textrm{Q}}^2} \int _{\textrm{Q}} \textrm{p}(q_1,q_2)\textrm{b}(q_1,q_2,\nu )\left[ \left( f^\textrm{Y}(\bar{q})\right) ^2 + 2 f^\textrm{Y}(\bar{q}) \left\langle f^\textrm{Y},\nu ^\textrm{Y}(s) \right\rangle \right] m^{\textrm{b}}(\bar{q}|q_1,q_2)d\bar{q}\nu ^\textrm{X}(s)(dq_1)\nu ^\textrm{X}(s)(dq_2)ds\,. \end{array}\right. } \end{aligned}$$
  At the same time, applying Itô formula to compute $\left\langle f,\nu \right\rangle ^2$, Eq. (32) yields that
  38 $$\begin{aligned} {\left\{ \begin{array}{ll} &{} \left\langle f^\textrm{X},\nu ^\textrm{X}(t) \right\rangle ^2 - \left\langle f^\textrm{X},\nu ^\textrm{X}_0 \right\rangle ^2 \\ &{}- \int _0^t \int _\textrm{Q}\left[ 2 \left\langle f^\textrm{X},\nu ^\textrm{X}(s) \right\rangle \left( \mu ^\textrm{X}\left( q, \left\langle \Gamma ^{\mu ,\textrm{X}}_{q},\nu \right\rangle \right) \cdot \nabla f^\textrm{X}\left( q\right) ds + \frac{1}{2} Tr\left[ \Sigma ^\textrm{X}\left( q, \left\langle \Gamma ^{\sigma ,\textrm{X}}_{q},\nu \right\rangle \right) \text{ Hess } \,f^\textrm{X}\left( q\right) \right] \right) \right] \nu ^\textrm{X}(s)(dq) \\ &{}+ \int _0^t\int _{\textrm{Q}} \left[ \textrm{r}(q,\nu ) + \textrm{a}(q,\nu )\right] f^\textrm{X}(q) \left\langle f^\textrm{X},\nu ^\textrm{X}(s) \right\rangle \nu ^\textrm{X}(s)(dq)ds\\ &{}+\int _0^t\int _{\tilde{\textrm{Q}}^2}\textrm{b}(q_1,q_2,\nu ) (f^\textrm{X}(q_1)+f^\textrm{X}(q_2)) \left\langle f^\textrm{X},\nu ^\textrm{X}(s) \right\rangle \nu ^\textrm{X}(s)(dq_1)\nu ^\textrm{X}(s)(dq_2)ds\\ &{} \left\langle f^\textrm{Y},\nu ^\textrm{Y}(t) \right\rangle - \left\langle f^\textrm{Y},\nu ^\textrm{Y}_0 \right\rangle \\ &{}- \int _0^t \int _\textrm{Q}\left[ 2 \left\langle f^\textrm{Y},\nu ^\textrm{Y}(s) \right\rangle \left( \mu ^\textrm{Y}\left( q, \left\langle \Gamma ^{\mu ,\textrm{Y}}_{q},\nu \right\rangle \right) \cdot \nabla f^\textrm{Y}\left( q\right) ds + \frac{1}{2} Tr\left[ \Sigma ^\textrm{Y}\left( q, \left\langle \Gamma ^{\sigma ,\textrm{Y}}_{q},\nu \right\rangle \right) \text{ Hess } \,f^\textrm{Y}\left( q\right) \right] \right) \right] \nu ^\textrm{Y}(s)(dq) ds\\ &{}- \int _0^t \int _{\textrm{Q}} \textrm{a}(q,\nu ) \int _{\textrm{Q}} m^{\textrm{a}}(\bar{q}|q) f^\textrm{Y}(\bar{q})d\bar{q} \left\langle f^\textrm{Y},\nu ^\textrm{Y}(s) \right\rangle \nu ^\textrm{X}(s)(dq)ds\\ &{}- \int _0^t\int _{\tilde{\textrm{Q}}^2} \int _{\textrm{Q}} \textrm{p}(q_1,q_2) \textrm{b}(q_1,q_2,\nu ) f^\textrm{Y}(\bar{q}) m^{\textrm{b}}(\bar{q}|q_1,q_2)d\bar{q} \left\langle f^\textrm{Y},\nu ^\textrm{Y}(s) \right\rangle \nu ^\textrm{X}(s)(dq_1)\nu ^\textrm{X}(s)(dq_2)ds - \textrm{M}^\textrm{Y}(t)\,, \end{array}\right. } \end{aligned}$$
  is a cádlág martingale. Comparing equations (37) and (38) implies Eq. (35).

$$\square $$

### On the initial distribution

As clear by the description given in Sect. 2.1, the initial distribution considered lacks any spatial distribution on the dose and on the formation of the lesion in the cell nucleus. To compute the initial lesion distribution $\nu _0$, we need to generalize the treatment given in Sect. 2.1 to include in Eq. (7) a spatial description. A possible mathematical formulation of the initial lesion computation would be the following. For a better understanding, we will provide a step-wise construction of such distribution:

(i) given a certain dose *D* and fluence average specific energy $z_F$, a random number of events $\nu$ in a cell nucleus is sampled from a distribution $p_e$. A typical assumption would be, due to the independence of events, to assume $p_e$ a Poisson distribution with average $\frac{D}{z_F}$;
(ii) the $\nu$ events are distributed randomly over the cell nucleus. Under an isotropic and uniform random field, the distribution can be assumed to be uniform over the domain, or in a more general setting, the distribution can be sampled from an *a priori* calculated distribution of tracks using a *condensed history* MC code (Agostinelli et al. 2003). A similar distribution has been for instance calculated in Missiaggia et al. (2021, 2022);
(iii) for any event *i*, $i=1,\dots ,\nu$, a certain specific energy $z_i$ is sampled according to the single-event microdosimetric specific energy distribution $f_1(z)$;
(iv) for any event *i*, $i=1,\dots ,\nu$, with specific energy deposition $z_i$, the number $\xi ^\textrm{X}_i$ and $\xi ^\textrm{Y}_i$ of sub-lethal and lethal lesion respectively is sampled from a distribution *p*. A typical assumption would be to assume such distribution the product of two independent Poisson distributions of average $\kappa (z_i)$ and $\lambda (z_i)$ respectively, for some suitable functions $\kappa$ and $\lambda$;
(v) denote by $\xi ^\textrm{X}:= \sum _{i=1}^\nu \xi ^\textrm{X}_i$ and $\xi ^\textrm{Y}:= \sum _{i=1}^\nu \xi ^\textrm{Y}_i$ the number of sub-lethal and lethal lesion respectively. Thus sample the positions $(q^\textrm{X},q^\textrm{Y}) \in \textrm{Q}^{|(\xi ^\textrm{X},\xi ^\textrm{Y})|}$, according to a distribution $\zeta _\xi (\left. q^\textrm{X},q^\textrm{Y}\right| \xi ^\textrm{X},\xi ^\textrm{Y})$. A reasonable choice for such distribution would be to distribute $z_i$ spatially around the track using the Amorphous Track model (Kase et al. 2007), which is a parametrization of the radial dose distribution around a particle track. In particular, denoting by $AT_i(q)$ the normalized radial dose distribution representing the probability of depositing a certain dose in a domain, for any $\textrm{Q}_1 \subset \textrm{Q}$, the relative dose absorbed in $\textrm{Q}_1$ is thus given by
  $$\begin{aligned} z_i\int _{\textrm{Q}_1}AT_i(q)dq\,. \end{aligned}$$
  Then, the probability density distribution describing the probability of creating a lesion in $\textrm{Q}_1$ is given by
  $$\begin{aligned} \sum _{i=1}^\nu z_i \int _{\textrm{Q}_1}AT_i(q)dq\,. \end{aligned}$$

#### Remark 3.11

As mentioned in the introduction, a further choice would be to use track structure code to simulate the spatial distribution of lesions within a cell nucleus. Several papers have been published in the literature showing how this can be achieved (Chatzipapas et al. 2022; Kyriakou et al. 2022; Zhu et al. 2020; Thibaut et al. 2023). Nonetheless, none of these papers then asses the biological effect of given radiation using a true spatial biological model, so the accuracy in describing the geometry of the biological target is lost.

## The protracted irradiation

In the present Section, we assume a further rate besides the interaction rates described in Sect. 3. Such a rate accounts for the formation of a new random number of both lethal and sub-lethal lesions due to protracted irradiation. We thus have the following system of possible pathways

39 $$\begin{aligned} {\left\{ \begin{array}{ll} &{} \textrm{X}\xrightarrow {\textrm{r}} \emptyset \,,\\ &{} \textrm{X}\xrightarrow {\textrm{a}} Y\,,\\ &{} \textrm{X}+ \textrm{X}\xrightarrow {\textrm{b}} {\left\{ \begin{array}{ll} \textrm{Y}&{} \text{ with } \text{ probability } \quad p \in [0,1]\,,\\ \emptyset &{} \text{ with } \text{ probability } \quad 1-p \in [0,1]\,,\\ \end{array}\right. }\,,\\ &{} \emptyset \xrightarrow {\dot{\textrm{d}}} {\left\{ \begin{array}{ll} \xi ^\textrm{X}\,\textrm{X}\,,\\ \xi ^\textrm{Y}\,\textrm{Y}\,, \end{array}\right. }\,. \end{array}\right. } \end{aligned}$$

where $\xi ^\textrm{X}$ and $\xi ^\textrm{Y}$ are two suitable (possibly correlated) $\mathbb {N}-$valued random variables. The last pathway, namely the dose-rate $\dot{\textrm{d}}$ is reasonable to be assumed strictly positive up to a certain time horizon $T_{irr}$, representing the irradiation period.

We thus have the following:

(v) - protracted irradiation at a certain *dose rate* $\dot{\textrm{d}}$ a random number $\xi ^\textrm{X}$ and $\xi ^\textrm{Y}$ sub-lethal and lethal lesions, respectively, are created in $\textrm{Q}$. This process can be described by a spatial compound random measure
  $$\begin{aligned} \zeta = (\zeta ^\textrm{X},\zeta ^\textrm{Y}) = \left( \sum _{i=0}^{\xi ^\textrm{X}} {\delta _{q^\textrm{X}_i},\sum _{j=0}^{\xi ^\textrm{Y}} \delta _{q^\textrm{Y}_j}} \right) \in \mathcal {M}\times \mathcal {M}\,. \end{aligned}$$
  We assume that the random measure $\zeta$ admits a probability measure of the form
  $$\begin{aligned} \zeta _\xi (\left. q^\textrm{X},q^\textrm{Y}\right| \xi ^\textrm{X},\xi ^\textrm{Y}) p(\xi ^\textrm{X},\xi ^\textrm{Y})\,, \end{aligned}$$
  with $p(\xi ^\textrm{X},\xi ^\textrm{Y})$ a discrete probability distribution on $\mathbb {N}^2$ representing the probability of inducing $(\xi ^\textrm{X},\xi ^\textrm{Y})$ sub-lethal and lethal lesions and $\zeta _\xi$ a spatial distribution representing the probability of creating $(\xi ^\textrm{X},\xi ^\textrm{Y})$ sub-lethal and lethal lesions at positions $( q^\textrm{X},q^\textrm{Y}) \in \textrm{Q}^{|(\xi ^\textrm{X},\xi ^\textrm{Y})|}$. We will further denote for short the marginal distributions by
  $$\begin{aligned} \begin{aligned} \zeta _{\xi ^\textrm{X}}(\left. q^\textrm{X}\right| \xi ^\textrm{X}) \,,\quad p_{\textrm{X}}(\xi ^\textrm{X})\,,\\ \zeta _{\xi ^\textrm{Y}}(\left. q^\textrm{Y}\right| \xi ^\textrm{Y}) \,,\quad p_{\textrm{Y}}(\xi ^\textrm{Y})\,. \end{aligned} \end{aligned}$$
  The *protracted irradiation rate* $\dot{\textrm{d}}$ is associated to a Poisson point measure
  $$\begin{aligned} \textrm{N}^{\dot{\textrm{d}}}(ds,d\xi ^\textrm{X},d\xi ^\textrm{Y},dq,d\theta _1,d\theta _2) \quad \text{ on } \quad \mathbb {R}_+ \times \mathbb {N}^2 \times \textrm{Q}^{|(\xi ^\textrm{X},\xi ^\textrm{Y})|} \times \mathbb {R}\times \mathbb {R}\,. \end{aligned}$$
  The corresponding intensity measure associated to $\textrm{N}^{\dot{\textrm{d}}}$ is
  $$\begin{aligned} \lambda ^{\dot{\textrm{d}}} (ds,d\xi ^\textrm{X},d\xi ^\textrm{Y},dq,d\theta _1,d\theta _2) := ds \otimes dp(\xi ^\textrm{X},\xi ^\textrm{Y}) \otimes dq \otimes d\theta _1 \otimes d\theta _2\,. \end{aligned}$$
  We denote with $\tilde{\textrm{N}}^\textrm{b}$ the compensated Poisson measure defined as
  $$\begin{aligned} \tilde{\textrm{N}}^{\dot{\textrm{d}}}(ds,d\xi ^\textrm{X},d\xi ^\textrm{Y},dq,d\theta _1,d\theta _2):= & {} \textrm{N}^{\dot{\textrm{d}}}(ds,d\xi ^\textrm{X},d\xi ^\textrm{Y},dq,d\theta _1,d\theta _2)\\{} & {} - \lambda ^{\dot{\textrm{d}}} (ds,d\xi ^\textrm{X},d\xi ^\textrm{Y},dq,d\theta _1,d\theta _2)\,. \end{aligned}$$

### Remark 4.1

The protracted irradiation can be interpreted as an improved description of the initial distribution. In particular, if $\dot{\textrm{d}}$ is sufficiently large and $T_{\text{ irr }}$ is sufficiently small, only the creation of new damages due to the protracted irradiation happens before any of the other pathways can happen. This is typically the case in the clinical scenario, where the *dose rate* usually dominates the biological interaction rates; such a situation is referred to as *conventional dose rate*. In this case, it is reasonable to assume that the initial distribution of lesions $\nu ^\textrm{X}_0$ and $\nu ^\textrm{Y}_0$ in the instantaneous irradiation, that is $\dot{\textrm{d}}= 0$, coincides with the distribution of lesions under protracted irradiation at time $T_0$. For this reason, a typical distribution $\zeta$ can be obtained following the description provided in Sect. 2.1 in the particular case of a single event hitting the domain, that is $\nu =1$. It is further worth stressing that there are some relevant situations where an explicit treatment of the effect of protracted irradiation can play a relevant role: (i) a *split dose* irradiation treatment, where the treatment is split into several treatments with a smaller dose to favorite normal tissue recovery between treatments, (ii) space radioprotection, characterized by extremely low dose rates exposure over a long period and (iii) FLASH radiotherapy. Both (i) and (ii) are situations where it is fundamental to model the entire spatial distribution of radiation-induced damages over a relatively long time period so that the inclusion of a specific protracted irradiation rate is necessary. The case that concerns (iii) will be explicitly treated in Sect. 4.1. $\triangle$

We will assume the following to hold.

### Hypothesis 4.2

4. protracted dose rate components:
  (4.i) the *protracted irradiation rate* $\dot{\textrm{d}}$ is positive and finite;
  (4.ii) for any $\xi ^\textrm{X}\,,\xi ^\textrm{Y}\in \mathbb {N}$, $\zeta _\xi$ is a probability measure, i.e.
    $$\begin{aligned} \int _{\textrm{Q}^{|(\xi ^\textrm{X},\xi ^\textrm{Y})|}} \zeta _\xi (\left. q^\textrm{X},q^\textrm{Y}\right| \xi ^\textrm{X},\xi ^\textrm{Y})dq^\textrm{X}\, dq^\textrm{Y}=1\,; \end{aligned}$$
  (4.iii) the random measure *p* admits finite $p-$moments, that is, for $p \ge 1$, it holds
    $$\begin{aligned} \int _{\mathbb {N}^2} \left( \xi ^\textrm{X}\right) ^p\,dp(\xi ^\textrm{X},\xi ^\textrm{Y})< \infty \,, \quad \int _{\mathbb {N}^2} \left( \xi ^\textrm{Y}\right) ^p\, dp(\xi ^\textrm{X},\xi ^\textrm{Y}) < \infty \,. \end{aligned}$$

Therefore, the resulting process is characterized by the process defined in Eq. (19) with the addition of the random measure $\textrm{N}^{\dot{\textrm{d}}}$. In particular, denote for short by $\mathcal {L}^\textrm{X}_B$ and $\mathcal {L}^\textrm{Y}_B$ the process introduced in equation (19), then the process under the effect of protracted irradiation is characterized by the following weak representation, given $f^\textrm{X}$ and $f^\textrm{Y}\in C^2_0(\mathbb {R})$, we have

40 $$\begin{aligned} {\left\{ \begin{array}{ll} \left\langle f^\textrm{X},\nu ^\textrm{X}(t) \right\rangle &{}= \mathcal {L}^\textrm{X}_B \nu ^\textrm{X}(t) \\ &{}+ \int _0^t \int _{\mathbb {N}^2} \int _{\textrm{Q}^{|(\xi ^\textrm{X},\xi ^\textrm{Y})|}} \int _{\mathbb {R}}\int _{\mathbb {R}} \left[ \left\langle f^\textrm{X},\nu ^{\textrm{X}}(s_-) + \sum _{i=1}^{\xi ^\textrm{X}} \delta _{q_i} \right\rangle - \left\langle f^\textrm{X},\nu ^{\textrm{X}}(s_-) \right\rangle \right] \\ &{}\qquad \times \mathbb {1}_{\left\{ \theta _1 \le \dot{\textrm{d}}\right\} }\mathbb {1}_{\left\{ \theta _2 \le {\zeta _\xi (q^\textrm{X},q^\textrm{Y}|\xi ^\textrm{X},\xi ^\textrm{Y})}\right\} } \textrm{N}^{\dot{\textrm{d}}}(ds,d\xi ^\textrm{X},d\xi ^\textrm{Y},dq,d\theta _1,d\theta _2)\,,\\ \left\langle f^\textrm{Y},\nu ^\textrm{Y}(t) \right\rangle &{}= \mathcal {L}^\textrm{Y}_B \nu ^\textrm{Y}(t) \\ &{}+\int _0^t \int _{\mathbb {N}^2} \int _{\textrm{Q}^{|(\xi ^\textrm{X},\xi ^\textrm{Y})|}} \int _{\mathbb {R}} \int _{\mathbb {R}} \left[ \left\langle f^\textrm{Y},\nu ^{\textrm{Y}}(s_-) + \sum _{i=1}^{\xi ^\textrm{Y}} \delta _{q_i} \right\rangle - \left\langle f^\textrm{Y},\nu ^{\textrm{Y}}(s_-) \right\rangle \right] \\ &{}\qquad \times \mathbb {1}_{\left\{ \theta _1 \le \dot{\textrm{d}}\right\} } \mathbb {1}_{\left\{ \theta _2 \le {\zeta _\xi (q^\textrm{X},q^\textrm{Y}|\xi ^\textrm{X},\xi ^\textrm{Y})}\right\} } \textrm{N}^{\dot{\textrm{d}}}(ds,d\xi ^\textrm{X},d\xi ^\textrm{Y},dq,d\theta _1,d\theta _2)\,. \end{array}\right. } \end{aligned}$$

We thus augment the probability space with the processes defined in (*v*). We thus have the following definition.

### Definition 4.2.1

We say that ${\varvec{\nu }}(t) = (\nu ^\textrm{X}(t),\nu ^\textrm{Y}(t))$ as defined in equations 9–10 is a *spatial radiation-induced DNA damage repair model under protracted irradiation* if ${\varvec{\nu }} = \left( {\varvec{\nu }}(t)\right) _{t \in \mathbb {R}_+}$ is $\left( \mathcal {F}_t\right) _{t \in \mathbb {R}_+}-$adapted and for any $f^\textrm{X}$ and $f^\textrm{Y}\in C^2_0(\textrm{Q})$ Eq. (40) holds $\mathbb {P}-$a.s.

We thus have the following well-posedness result.

### Theorem 4.3

Let $\nu ^\textrm{X}_0$ and $\nu ^\textrm{Y}_0$ two random measures with finite $p-$th moment, $p \ge 1$, that is it holds

41 $$\begin{aligned} \mathbb {E} \left\langle \textbf{1},\nu ^\textrm{X}_0 \right\rangle ^p< \infty \,,\quad \mathbb {E} \left\langle \textbf{1},\nu ^\textrm{Y}_0 \right\rangle ^p < \infty \,. \end{aligned}$$

Then, under Hypothesis 3.4–4.2, for any $T>0$ it exists a unique strong solution in $\mathcal {D}\left( [0,T],\mathcal {M}\times \mathcal {M}\right)$ to the system (19). Also, it holds

42 $$\begin{aligned} \mathbb {E} \sup _{t \le T} \left\langle \textbf{1},\nu (t) \right\rangle ^p < \infty \,. \end{aligned}$$

### Proof

The proof proceeds with similar arguments as in the proof of Theorem 3.8, taking into account the protracted irradiation term.

For $t \ge 0$, we have,

43 $$\begin{aligned} \begin{aligned}&\sup _{s \in [0,\bar{\tau }_n^\textrm{X}]} \left\langle \textbf{1},\nu ^\textrm{X}(s) \right\rangle ^p \le \left\langle \textbf{1},\nu ^\textrm{X}_0 \right\rangle ^p + \\&+\int _0^{\bar{\tau }_n^\textrm{Y}} \int _{\mathbb {N}_0} \int _{\textrm{Q}} \int _{\mathbb {R}_+^2} \left[ \left( \left\langle \textbf{1},\nu ^{\textrm{Y}}(s_-) \right\rangle + 1\right) ^p - \left\langle \textbf{1},\nu ^{\textrm{Y}}(s_-) \right\rangle ^p\right] \\&\times \mathbb {1}_{\left\{ i \le N^\textrm{X}(s_-)\right\} }\mathbb {1}_{\left\{ \theta _1 \le \textrm{a}\left( \textrm{H}^i\left( \nu ^\textrm{X}(s_-)\right) , \left\langle \Gamma ^\textrm{a},\nu \right\rangle \right) \right\} } \mathbb {1}_{\left\{ \theta _2 \le m^\textrm{a}\left( q|\textrm{H}^i\left( \nu ^\textrm{X}(s_-)\right) \right) \right\} } \textrm{N}^\textrm{a}(ds,di,dq,d\theta _1,d\theta _2)\\&+ \int _0^{\bar{\tau }_n^\textrm{Y}} \int _{\mathbb {N}_0^2} \int _{\textrm{Q}} \int _{\mathbb {R}_+^2} \left[ \left( \left\langle \textbf{1},\nu ^{\textrm{Y}}(s_-) \right\rangle + 1\right) ^p - \left\langle \textbf{1},\nu ^{\textrm{Y}}(s_-) \right\rangle ^p\right] \\&\times \mathbb {1}_{\left\{ i_1 < i_2 \le N^\textrm{X}(s_-)\right\} }\mathbb {1}_{\left\{ \theta _1 \le \textrm{b}\left( \textrm{H}^{i_1}\left( \nu ^\textrm{X}(s_-)\right) ,\textrm{H}^{i_2}\left( \nu ^\textrm{X}(s_-)\right) , \left\langle \Gamma ^\textrm{b},\nu \right\rangle \right) \right\} } \mathbb {1}_{\left\{ \theta _2 \le m^\textrm{b}(q|\textrm{H}^{i_1}\left( \nu ^\textrm{X}(s_-)\right) ,\textrm{H}^{i_2}\left( \nu ^\textrm{X}(s_-)\right) )\right\} } \textrm{N}^{\textrm{b};p}(ds,di_1,di_2,dq,d\theta _1,d\theta _2)\\&+\int _0^{\bar{\tau }_n^\textrm{X}}\int _{\mathbb {N}^2} \int _{\textrm{Q}^{|(\xi ^\textrm{X},\xi ^\textrm{Y})|}} \int _\mathbb {R}\int _\mathbb {R}\left[ \left\langle f^\textrm{X},\nu ^{\textrm{X}}(s_-) + \sum _{i=1}^{\xi ^\textrm{X}} \delta _{q_i} \right\rangle - \left\langle f^\textrm{X},\nu ^{\textrm{X}}(s_-) \right\rangle \right] ^p \\&\qquad \times \mathbb {1}_{\left\{ \theta _1 \le \dot{\textrm{d}}\right\} }\mathbb {1}_{\left\{ \theta _2 \le \zeta _\xi (q^\textrm{X},q^\textrm{Y}|\xi ^\textrm{X},\xi ^\textrm{Y})\right\} } \textrm{N}^{\dot{\textrm{d}}}(ds,d\xi ^\textrm{X},d\xi ^\textrm{Y},dq,d\theta _1,d\theta _2)\,. \end{aligned} \end{aligned}$$

Notice that, for any positive integer *x* and *y* it holds

44 $$\begin{aligned} (x+y)^p - y^p \le C_{p}y^{p-1}x^{p}\,, \end{aligned}$$

so that, using Eq. (44) into equation (43), we have that, for some $C>0$ that can take possibly different values, and using the bound proven in equation (28),

45 $$\begin{aligned} \begin{aligned}&\mathbb {E}\sup _{s \in [0,\bar{\tau }_n^\textrm{X}]} \left\langle \textbf{1},\nu ^\textrm{X}(s) \right\rangle ^p\\&\quad \le \mathbb {E} \left\langle \textbf{1},\nu ^\textrm{X}_0 \right\rangle ^p \\&\qquad +C \mathbb {E}\int _0^{\bar{\tau }_n^\textrm{Y}} \left( 1+ \left\langle \textbf{1},\nu ^{\textrm{Y}}(s_-) \right\rangle ^{p-1} \right) \left\langle \textbf{1},\nu ^{\textrm{Y}}(s_-) \right\rangle ds \\&\qquad +C \dot{\textrm{d}}\mathbb {E}\int _0^{\bar{\tau }_n^\textrm{X}} \int _{\mathbb {N}^2}\int _{\textrm{Q}^{|(\xi ^\textrm{X},\xi ^\textrm{Y})|}} \left( \xi ^\textrm{X}\right) ^p \left\langle \textbf{1},\nu ^{\textrm{X}}(s_-) \right\rangle ^{p-1}\zeta _\xi (\left. q^\textrm{X},q^\textrm{Y}\right| \xi ^\textrm{X},\xi ^\textrm{Y}) dq^\textrm{X}\,dq^\textrm{Y}\,dp(\xi ^\textrm{X},\xi ^\textrm{Y})ds \le \\&\quad \le C \left( 1+ \mathbb {E}\int _0^t \left\langle \textbf{1},\nu ^{\textrm{X}}(s \wedge \tau _n^\textrm{Y}) \right\rangle ^p \right) \,. \end{aligned} \end{aligned}$$

Gronwall lemma implies that there exists $C>0$ depending on p and *T* but independent of *n*, such that

46 $$\begin{aligned} \mathbb {E}\sup _{s \in [0,\bar{\tau }_n^\textrm{X}]} \left\langle \textbf{1},\nu ^\textrm{X}(s) \right\rangle ^p \le C\,. \end{aligned}$$

Similar arguments can be used to prove that

47 $$\begin{aligned} \mathbb {E}\sup _{s \in [0,\bar{\tau }_n^\textrm{Y}]} \left\langle \textbf{1},\nu ^\textrm{Y}(s) \right\rangle ^p \le C\,. \end{aligned}$$

The proof thus follows in a straightforward manner proceeding as in the proof of Theorem 3.8. $\square$

The above process is characterized by the following infinitesimal generator

48 $$\begin{aligned} \mathcal {L}F_{(f^\textrm{X},f^\textrm{Y})}(\nu ) = \mathcal {L}_d F_{(f^\textrm{X},f^\textrm{Y})}(\nu ) + \sum _{h \in \{\textrm{r},\textrm{a},\textrm{b},\dot{\textrm{d}}\}} \mathcal {L}_{h} F_{(f^\textrm{X},f^\textrm{Y})}(\nu )\,, \end{aligned}$$

where $\mathcal {L}_d F_{(f^\textrm{X},f^\textrm{Y})}(\nu )$ and $\mathcal {L}_h F_{(f^\textrm{X},f^\textrm{Y})}(\nu )$, $h \in \{\textrm{r},\textrm{a},\textrm{b},\dot{\textrm{d}}\}$, are the infinitesimal generators introduced in equations (22)–(23), whereas $\mathcal {L}_{\dot{\textrm{d}}}$ is defined as

49 $$\begin{aligned} \mathcal {L}_{\dot{\textrm{d}}} F_{(f^\textrm{X},f^\textrm{Y})}(\nu )= & {} \dot{\textrm{d}}\int _{\mathbb {N}^2} \int _{\textrm{Q}^{|(\xi ^\textrm{X},\xi ^\textrm{Y})|}} \left[ F_{(f^\textrm{X},f^\textrm{Y})}\left( \nu ^\textrm{X}+\sum _{i=1}^{\xi ^\textrm{X}}\delta _{q_i^\textrm{X}},\nu ^\textrm{Y}+\sum _{i=1}^{\xi ^\textrm{Y}}\delta _{q_i^\textrm{Y}}\right) - F_{(f^\textrm{X},f^\textrm{Y})}(\nu ) \right] \nonumber \\{} & {} \times \zeta _\xi (\left. q^\textrm{X},q^\textrm{Y}\right| \xi ^\textrm{X},\xi ^\textrm{Y})dq^\textrm{X}\,dq^\textrm{Y}dp(\xi ^\textrm{X},\xi ^\textrm{Y}) \,. \end{aligned}$$

We thus have the martingale properties and representation corresponding to Theorem 3.10.

### Theorem 4.4

Assume that Hypothesis 3.4–4.2 holds true and that $\nu ^\textrm{X}_0$ and $\nu ^\textrm{Y}_0$ are two random measures independent with finite $p-$th moment, $p \ge 2$. Then:

(i) ${\varvec{\nu }}$ is a Markov process with infinitesimal generator $\mathcal {L}$ defined by (48);
(ii) assume that for $F \in C^2_b (\mathbb {R}\times \mathbb {R})$ and for $f^\textrm{X}\,,\,f^\textrm{Y}\in {\mathcal {C}^{2}_0}(\textrm{Q})$ such that for all ${\varvec{\nu }} \in \mathcal {M}\times \mathcal {M}$, it holds
  50 $$\begin{aligned} |F_{(f^\textrm{X},f^\textrm{Y})}(\nu )| + |\mathcal {L}F_{(f^\textrm{X},f^\textrm{Y})}(\nu )| \le C \left( 1+ \left\langle \textbf{1},\nu _0 \right\rangle ^p \right) \,. \end{aligned}$$
  Then, the process
  51 $$\begin{aligned} F_{(f^\textrm{X},f^\textrm{Y})}({\varvec{\nu }}(t)) - F_{(f^\textrm{X},f^\textrm{Y})}({\varvec{\nu }}_0) - \int _0^t \mathcal {L}F_{(f^\textrm{X},f^\textrm{Y})}({\varvec{\nu }}(s))ds\,, \end{aligned}$$
  is a cádlág martingale starting at 0;
(iii) the processes $\textrm{M}^\textrm{X}$ and $\textrm{M}^\textrm{Y}$ defined for $f^\textrm{X}\,,\,f^\textrm{Y}\in C^2_0$ by
  52 $$\begin{aligned} {\left\{ \begin{array}{ll} \textrm{M}^\textrm{X}(t) &{}= \left\langle f^\textrm{X},\nu ^\textrm{X}(t) \right\rangle - \left\langle f^\textrm{X},\nu ^\textrm{X}_0 \right\rangle - \int _0^t \left\langle \mathcal {L}^\textrm{X}_d f^\textrm{X}(x),\nu ^\textrm{X}(s) \right\rangle ds \\ &{}+ \int _0^t\int _{\textrm{Q}} \left[ \textrm{r}(q,\nu ) + \textrm{a}(q,\nu ) \right] f^\textrm{X}(q)\nu ^\textrm{X}(s)(dq)ds\\ &{}+\int _0^t\int _{\tilde{\textrm{Q}}^2} \textrm{b}(q_1,q_2,\nu ) (f^\textrm{X}(q_1)+f^\textrm{X}(q_2))\nu ^\textrm{X}(s)(dq_1)\nu ^\textrm{X}(s)(dq_2)ds\\ &{}-\int _0^t \int _{\mathbb {N}} \int _{\tilde{\textrm{Q}}^{\xi ^\textrm{X}}} \dot{\textrm{d}}\left( \sum _{i=1}^{\xi ^\textrm{X}}f^\textrm{X}(q_i^\textrm{X}) \right) \zeta _{\xi ^\textrm{X}}(\left. q^\textrm{X}\right| \xi ^\textrm{X}) dq^\textrm{X}dp(\xi ^\textrm{X},\xi ^\textrm{Y}) ds\,,\\ \textrm{M}^\textrm{Y}(t) &{}= \left\langle f^\textrm{Y},\nu ^\textrm{Y}(t) \right\rangle - \left\langle f^\textrm{Y},\nu ^\textrm{Y} \right\rangle - \int _0^t \left\langle \mathcal {L}^\textrm{Y}_d f^\textrm{Y}(x),\nu ^\textrm{Y}(s) \right\rangle ds \\ &{}- \int _0^t \int _{\textrm{Q}} \textrm{a}(q,\nu ) \int _{\textrm{Q}} m^{\textrm{a}}(\bar{q}|q) f^\textrm{Y}(\bar{q})d\bar{q} \nu ^\textrm{X}(s)(dq)ds\\ &{}- \int _0^t\int _{\tilde{\textrm{Q}}^2} \int _{\textrm{Q}} \textrm{p}(q_1,q_2) \textrm{b}(q_1,q_2,\nu ) f^\textrm{Y}(\bar{q}) m^{\textrm{b}}(\bar{q}|q_1,q_2)d\bar{q}\nu ^\textrm{X}(s)(dq_1)\nu ^\textrm{X}(s)(dq_2)ds\\ &{}-\int _0^t \int _{\mathbb {N}} \int _{\tilde{\textrm{Q}}^{\xi ^\textrm{Y}}} \dot{\textrm{d}}\left( \sum _{i=1}^{\xi ^\textrm{Y}} f^\textrm{Y}(q_i^\textrm{Y}) \right) \zeta _{\xi ^\textrm{Y}}(\left. q^\textrm{Y}\right| \xi ^\textrm{Y}) dq^\textrm{Y}dp(\xi ^\textrm{X},\xi ^\textrm{Y}) ds\,, \end{array}\right. } \end{aligned}$$
  are cádlág $L^2-$martingale starting at 0 with predictable quadratic variation given by
  53 $$\begin{aligned} {\left\{ \begin{array}{ll} \left\langle \textrm{M}^\textrm{X} \right\rangle (t) &{}= \int _0^t \left\langle \nabla ^T f^\textrm{X}\sigma ^\textrm{X}\nabla f^\textrm{X}, \nu ^\textrm{X} \right\rangle ds \\ &{}+ \int _0^t \int _{\textrm{Q}} \left[ \textrm{r}(q,\nu ) + \textrm{a}(q,\nu )\right] \left( f^\textrm{X}(q) \right) ^2 \nu ^\textrm{X}(s)(dq)ds\\ &{} +\int _0^t\int _{\tilde{\textrm{Q}}^2} \textrm{b}(q_1,q_2,\nu ) (f^\textrm{X}(q_1)+f^\textrm{X}(q_2))^2 \nu ^\textrm{X}(s)(dq_1)\nu ^\textrm{X}(s)(dq_2)ds\\ &{}-\int _0^t \int _{\mathbb {N}} \int _{\tilde{\textrm{Q}}^{\xi ^\textrm{X}}} \dot{\textrm{d}}\left( \sum _{i=1}^{\xi ^\textrm{X}}f^\textrm{X}(q_i^\textrm{X}) \right) ^2 \zeta _{\xi ^\textrm{X}}(\left. q^\textrm{X}\right| \xi ^\textrm{X})dq^\textrm{X}dp(\xi ^\textrm{X},\xi ^\textrm{Y}) ds\,,\\ \left\langle \textrm{M}^\textrm{Y} \right\rangle (t) &{}= \int _0^t \left\langle \nabla ^T f^\textrm{Y}\sigma ^\textrm{Y}\nabla f^\textrm{Y}, \nu ^\textrm{Y} \right\rangle ds \\ &{}- \int _0^t \int _{\textrm{Q}} \textrm{a}(q,\nu ) \int _{\textrm{Q}} m^{\textrm{a}}(\bar{q}|q) \left( f^\textrm{Y}(\bar{q})\right) ^2 d\bar{q} \nu ^\textrm{X}(s)(dq)ds\\ &{}- \int _0^t\int _{\tilde{\textrm{Q}}^2} \int _{\textrm{Q}} \textrm{p}(q_1,q_2) \textrm{b}(q_1,q_2,\nu ) \left( f^\textrm{Y}(\bar{q})\right) ^2 m^{\textrm{b}}(\bar{q}|q_1,q_2)d\bar{q}\nu ^\textrm{X}(s)(dq_1)\nu ^\textrm{X}(s)(dq_2)ds\\ &{}-\int _0^t \int _{\mathbb {N}} \int _{\tilde{\textrm{Q}}^{\xi ^\textrm{Y}}} \dot{\textrm{d}}\left( \sum _{i=1}^{\xi ^\textrm{Y}} f^\textrm{Y}(q_i^\textrm{Y}) \right) ^2 \zeta _{\xi ^\textrm{Y}}(\left. q^\textrm{Y}\right| \xi ^\textrm{Y})dq^\textrm{Y}dp(\xi ^\textrm{X},\xi ^\textrm{Y}) ds\,, \end{array}\right. } \end{aligned}$$

### Proof

The proof is analogous to the proof of Theorem 3.10. $\square$

### The bio-chemical system under protracted irradiation

As mentioned above, before focusing on a macroscopic limit of the spatial DNA repair model, we will consider a different setting relevant to the considered application. As commented in Sect. 2.1, the functions $\kappa$ and $\lambda$ usually include information regarding the chemical environment and radical formation. In the following treatment, we make this assumption explicit, assuming that the formation of new damages depends on the chemical environment described by a set of reaction–diffusion equations. It is worth stressing that in general, the energy deposition of the particle also affects the chemical environment so the above-mentioned reaction–diffusion equation also includes a term dependent on the energy deposition. As the chemical evolves on a much faster time scale, the concentration of chemical species will be described by a set of parabolic PDE, with a random discontinuous inhomogeneous term due to the effect of radiation. We will not consider a specific model for the chemical environment, but on the contrary, we will assume a general *mass control hypothesis* that includes many possible systems proposed in the literature. Future investigation will be specifically devoted to analyzing and implementing the highly dimensional chemical system, including the homogeneous chemical stage also the heterogeneous one.

Assume a set of *L* chemical species, then, for $i=1,\dots ,L$, the concentration of the $i-$th species $\rho _i$ evolves according to

54 $$\begin{aligned} {\left\{ \begin{array}{ll} \frac{\partial }{\partial t} \rho _i(q, t)= D_i \Delta _q \rho _i(q, t) + f_i(\rho ) + G_i(q)\,, &{} \quad \text{ in } \quad \textrm{Q}\times [0,T]\,,\\ \nabla _q \rho _i \cdot n(q) = 0 \,, &{} \quad \text{ in } \quad \partial \textrm{Q}\times (0,T)\,,\\ \rho _i(0,q)= \rho _{0;i}(q) \,, &{} \quad \text{ in } \quad \textrm{Q}\,.\\ \end{array}\right. } \end{aligned}$$

We consider the following.

$$(v') - protracted irradiation$$
at a certain *dose rate* $\dot{\textrm{d}}$ a random number $\xi ^\textrm{X}$ and $\xi ^\textrm{Y}$ sub-lethal and lethal lesions, respectively, are created in $\textrm{Q}$. This process is described by a random measure that depends on chemical concentration
  $$\begin{aligned} \zeta = (\zeta ^\textrm{X},\zeta ^\textrm{Y}) = \left( \sum _{i=0}^{\xi ^\textrm{X}} \delta _{\textrm{Q}_i},\sum _{i=0}^{\xi ^\textrm{Y}} \delta _{\textrm{Q}_i} \right) \in \mathcal {M}\times \mathcal {M}\,. \end{aligned}$$
  We assume that the random measure $\zeta$ admits a decomposition of the form
  $$\begin{aligned} \zeta _\xi (\left. q^\textrm{X},q^\textrm{Y}\right| \xi ^\textrm{X},\xi ^\textrm{Y}) p\left( \left. \xi ^\textrm{X},\xi ^\textrm{Y}\right| \rho (q, t) \right) \,, \end{aligned}$$
  with $p(\xi ^\textrm{X},\xi ^\textrm{Y})$ a discrete probability distribution on $\mathbb {N}^2$.
$$(vi) - chemical environment$$
for all $i=1,\dots ,L$, the random discontinuous inhomogeneous term *G* is defined as
  $$\begin{aligned} G_i : \Omega \times \textrm{Q}\rightarrow \mathbb {R}_+\,,\quad \Omega \times \textrm{Q}\ni (\omega ,q) \mapsto \sum _{k=1}^{\infty } \textrm{Z}^{k;i}(q,\omega )\delta _{\tau ^k_{\dot{\textrm{d}}(\omega )}}\,. \end{aligned}$$

#### Remark 4.5

The random function $Z^i$ represents the energy deposited by an event and can be computed as described in Sect. 2.1. Regarding the dependence of *p* on the chemical environment, several possible choices can be made. What is currently known is that the actual number of damages created by certain radiation depends on the chemical environment. Therefore, considering the description of the initial damage distribution given in Sect. 2.1, a meaningful choice would be to assume that given a certain specific energy deposition, the average number of induced lethal and sub-lethal lesions described in (*iv*) depends on the chemical environment, that is we have $\lambda (z_i,\rho )$ and $\kappa (z_i,\rho _i)$. $\triangle$

We will assume the following to hold.

#### Hypothesis 4.6

5. chemical environment components:
  (5.i) for all $i=1,\dots ,L$, the random initial condition $\rho _{0;i}$ is bounded and non-negative $\mathbb {P}-$a.s., that is
    $$\begin{aligned} \rho _{0;i}(q,\omega ) \in L^\infty (\textrm{Q})\,,\quad \text{ and } \quad \rho _{0;i}(q,\omega ) \ge 0\,, \quad \text{ for } \text{ a.e. } q \in \textrm{Q}\,,\quad \mathbb {P}-\text{ a.s. }\,, \end{aligned}$$
    and has finite $p-$th moment, $p \ge 1$, that is
    $$\begin{aligned} \mathbb {E}\left\| \rho _{0;i}\right\| ^p_\infty < \infty \,. \end{aligned}$$
  (5.ii) there exist constants $C_0$ and $C_1$ such that
    $$\begin{aligned} \sum _{i=1}^L f_i(\rho ) \le C_0 + C_1 \sum _{i=1}^L \rho _i\,; \end{aligned}$$
  (5.iii) for all $i=1,\dots ,L$, $f_i$ is locally Lipschitz and
    $$\begin{aligned} f_i(\rho ) \ge 0 \,,\quad \text{ for } \text{ all } \quad \rho =0\,; \end{aligned}$$
  (5.iv) for all $i=1,\dots ,L$, there exist $\varepsilon >0$ and $K>0$ such that
    $$\begin{aligned} |f_i(\rho )| \le K(1+|\rho |^{2+\varepsilon })\,; \end{aligned}$$
  (5.v) for all $i=1,\dots ,L$, the random function $Z^i$ is bounded and non-negative $\mathbb {P}-$a.s., that is
    $$\begin{aligned} Z^i(q,\omega ) \in L^\infty (\textrm{Q}) \,,\quad \text{ and } \quad Z^i(q,\omega ) \ge 0\,, \quad \text{ for } \text{ a.e. } q \in \textrm{Q}\,, \quad \mathbb {P}-\text{ a.s. }\,, \end{aligned}$$
    and has finite $p-$th moment, $p \ge 1$, that is
    $$\begin{aligned} \mathbb {E}\left\| Z^i\right\| ^p_\infty < \infty \,. \end{aligned}$$

#### Remark 4.7

As mentioned in the Introduction, we will not focus on specific examples of chemical environments. Nonetheless, since almost any chemical model contains second-order reaction rates, we are forced to consider more general assumptions than the standard global Lipschitz condition. For this reason, we rather consider a mass control condition in Hypothesis 4.6(ii). Such assumptions can be immediately seen to hold in a reaction–diffusion description of the systems introduced in Abolfath et al. (2020a); Labarbe et al. (2020). $\triangle$

Therefore, the resulting process is characterized by the process defined in Eq. (40) with the addition of the chemical system as defined in Eq. (54). We thus have the following representation

55 $$\begin{aligned} {\left\{ \begin{array}{ll} \left\langle f^\textrm{X},\nu ^\textrm{X}(t) \right\rangle &{}= \mathcal {L}^\textrm{X}_B \nu ^\textrm{X}(t) \\ &{}+ \int _0^t \int _{\textrm{Q}^{|(\xi ^\textrm{X},\xi ^\textrm{Y})|}} \int _{\mathbb {N}^2} \int _{\mathbb {R}}\int _{\mathbb {R}} \left[ \left\langle f^\textrm{X},\nu ^{\textrm{X}}(s_-) + \sum _{i=1}^{\xi ^\textrm{X}} \delta _{q_i^\textrm{X}} \right\rangle - \left\langle f^\textrm{X},\nu ^{\textrm{X}}(s_-) \right\rangle \right] \\ &{}\qquad \times \mathbb {1}_{\left\{ \theta _1 \le \dot{\textrm{d}}\right\} }\mathbb {1}_{\left\{ \theta _2 \le \zeta _\xi ^\textrm{X}(q^\textrm{X},q^\textrm{Y}|,\xi ^\textrm{X},\xi ^\textrm{Y},\rho )\right\} } \textrm{N}^{\dot{\textrm{d}}}(ds,dq,d\xi ^\textrm{X},d\xi ^\textrm{Y},d\theta _1,d\theta _2)\,,\\ \left\langle f^\textrm{Y},\nu ^\textrm{Y}(t) \right\rangle &{}= \mathcal {L}^\textrm{Y}_B \nu ^\textrm{Y}(t) \\ &{}+\int _0^t \int _{\textrm{Q}^{|(\xi ^\textrm{X},\xi ^\textrm{Y})|}} \int _{\mathbb {N}^2} \int _{\mathbb {R}} \int _{\mathbb {R}} \left[ \left\langle f^\textrm{Y},\nu ^{\textrm{Y}}(s_-) + \sum _{i=1}^{\xi ^\textrm{Y}} \delta _{q_i^\textrm{Y}} \right\rangle - \left\langle f^\textrm{Y},\nu ^{\textrm{Y}}(s_-) \right\rangle \right] \\ &{}\qquad \times \mathbb {1}_{\left\{ \theta _1 \le \dot{\textrm{d}}\right\} } \mathbb {1}_{\left\{ \theta _2 \le \zeta _\xi (q^\textrm{X},q^\textrm{Y}|,\xi ^\textrm{X},\xi ^\textrm{Y},\rho )\right\} } \textrm{N}^{\dot{\textrm{d}}}(ds,dq,d\xi ^\textrm{X},d\xi ^\textrm{Y},d\theta _1,d\theta _2)\,,\\ \rho _i(q, t) &{}= \rho _{0;i}(q) + \int _0^t \left( D_i \Delta _q \rho _i(q, s) + f_i(\rho )\right) ds + \sum _{s<t} \sum _{k=1}^{\infty } \textrm{Z}^k(q) \mathbb {1}_{\left\{ s = \tau ^k_{\dot{\textrm{d}}}\right\} }\,. \end{array}\right. } \end{aligned}$$

Augment then the filtration with the processes defined in $(v')-(vi)$.

#### Definition 4.7.1

We say that ${\varvec{\nu }}(t) = (\nu ^\textrm{X}(t),\nu ^\textrm{Y}(t))$ as defined in equations 9–10 is a *spatial radiation-induced DNA damage repair model under protracted irradiation* if ${\varvec{\nu }} = \left( {\varvec{\nu }}(t)\right) _{t \in \mathbb {R}_+}$ is $\left( \mathcal {F}_t\right) _{t \in \mathbb {R}_+}-$adapted and for any $f^\textrm{X}$ and $f^\textrm{Y}\in C^2_0(\textrm{Q})$ Eq. (55) holds $\mathbb {P}-$a.s.

We thus have the following well-posedness result.

#### Theorem 4.8

Let $\nu ^\textrm{X}_0$ and $\nu ^\textrm{Y}_0$ two random measures with finite $p-$th moment, $p \ge 1$, that is it holds

56 $$\begin{aligned} \mathbb {E} \left\langle \textbf{1},\nu ^\textrm{X}_0 \right\rangle ^p< \infty \,,\quad \mathbb {E} \left\langle \textbf{1},\nu ^\textrm{Y}_0 \right\rangle ^p < \infty \,. \end{aligned}$$

Then, under Hypothesis 3.4–4.2–4.6, for any $T>0$ it exists a unique strong solution of the system (19) in $\mathcal {D}\left( [0,T],\mathcal {M}\times \mathcal {M}\times \left( L^\infty (\textrm{Q}) \right) ^L \right)$. Also, it holds

57 $$\begin{aligned} \mathbb {E} \sup _{t \le T} \left\langle \textbf{1},\nu (t) \right\rangle ^p< \infty \,, \quad \mathbb {E} \sup _{t \le T} \sup _{i=1\,,\dots ,L} \left\| \rho _i\right\| ^p_\infty < \infty \,. \end{aligned}$$

#### Proof

The main step of the proof follows alike Theorems 3.8–4.3, noticing that between consecutive jump times $T_m$ and $T_{m+1}$, under Hypothesis 4.6 using (Fellner et al. 2020, Theorem 1.1), equation (54) admits a unique strong solution in $C\left( [T_m,T_{m+1});\left( L^\infty (\textrm{Q}) \right) ^L \right)$. In fact, since *Z* is bounded and non-negative $\mathbb {P}-$a.s. we can infer that for any jump time $T_m$ it holds

$$\begin{aligned} \begin{aligned} \rho _i(q,T_{m})&:= \lim _{t \uparrow T_{m}}\rho _i(q,t) + \textrm{Z}(q)\,,\\ \rho _i(q,T_{m},\omega ) \in L^\infty (\textrm{Q})\,,&\quad \text{ and } \quad \rho _i(q,T_{m},\omega ) \ge 0\,, \quad \text{ for } \text{ a.e. } q \in \textrm{Q}\,,\quad \mathbb {P}-\text{ a.s. }\,. \end{aligned} \end{aligned}$$

The rest of the proof follows Theorems 3.8–4.3. $\square$

#### Remark 4.9

It is worth noticing that, using (Fellner et al. 2020, Theorem1.1), we can infer that, between consecutive jump times $T_m$ and $T_{m+1}$, it holds $\rho \in C\left( (T_m,T_{m+1});\left( C^2_0(\textrm{Q}) \right) ^L \right)$. Nonetheless, since we cannot require *Z* to be smooth, we cannot conclude that $\rho \in \mathcal {D}\left( (0,T);\left( C^2_0(\textrm{Q}) \right) ^L \right)$ as at the jump times $T_m$, $\rho (T_m,q)$ can only be shown to be bounded. $\triangle$

## The large population limit

In the following, we assume the model coefficients depend on a parameter $\textrm{K}$ and study the behavior of the measure-valued solution $\nu ^\textrm{K}= \left( \nu ^{\textrm{X};\textrm{K}},\nu ^{\textrm{Y};\textrm{K}}\right)$ studied in previous Sections as $\textrm{K}\rightarrow \infty$.

We thus consider a sequence of the initial measure so that $\frac{1}{\textrm{K}}\left( \nu ^{\textrm{X};\textrm{K}}_0,\nu ^{\textrm{Y};\textrm{K}}_0\right) \rightarrow \left( u^\textrm{X}_0,u^\textrm{Y}_0\right)$ and we assume that the rates of the model as introduced in Sects. 3–4 are rescaled as follows

$$\begin{aligned} \begin{aligned} \textrm{a}^\textrm{K}(q,v) := \textrm{a}\left( q,\frac{v}{\textrm{K}}\right) \,,\quad&\textrm{r}^\textrm{K}(q,v) := \textrm{r}\left( q,\frac{v}{\textrm{K}}\right) \,,\\ \textrm{b}^\textrm{K}(q_1,q_2,v) := \frac{1}{\textrm{K}}\textrm{b}\left( q_1,q_2,\frac{v}{\textrm{K}}\right) \,,\quad&\dot{\textrm{d}}^\textrm{K}:= \textrm{K}\dot{\textrm{d}}\,.\\ \end{aligned} \end{aligned}$$

### Remark 5.1

The typical assumption for the dose rate is $\dot{d} = \frac{D}{T_{irr}} \frac{1}{z_F}$ (Cordoni et al. 2021). Since the single event mean specific energy $z_F$ is defined as energy over mass, it decreases with the increase of the mass, so it is reasonable to assume that $\dot{d}$ scale with the parameter $\textrm{K}$. Nonetheless, other reasonable choices can be made, such as assuming that $\dot{d}$ is independent of $\textrm{K}$. This assumption would imply that the limiting equation is no longer deterministic as the quadratic variation of the process would not vanish, and the irradiation times remain discrete. Such a case could be relevant for the case of extremely low dose rates and low doses considered in space radioprotection. Nonetheless, in such a case it would be more relevant to look at less severe biological endpoints than DSB and clustered DSB, where particular interest would be on SSB or potentially cancerogenic mutations. For this reason, the case of $\dot{d}$ independent of $\textrm{K}$ will be left for a future study.

We thus aim at characterizing the limit as $\textrm{K}\rightarrow \infty$ of the rescaled measure

58 $$\begin{aligned} u^\textrm{K}(t):=\frac{1}{\textrm{K}}\sum _{i=1}^{N(t)} \delta _{Q_i(t)}\delta _{s_i} = \frac{1}{\textrm{K}}\nu ^\textrm{K}(t)\,. \end{aligned}$$

As above we also define for short the marginal distributions

59 $$\begin{aligned} u^{\textrm{X};\textrm{K}}(t)(\cdot ) := u^\textrm{K}(t)\left( \, \cdot \,,\textrm{X}\right) \,,\quad u^{\textrm{Y};\textrm{K}}(t)(\cdot ) := u^\textrm{K}(t)\left( \, \cdot \,,\textrm{Y}\right) \,. \end{aligned}$$

Analogous arguments to the one derived in Sects. 3–4 show that $\big (\nu ^{\textrm{X};\textrm{K}}(t),\nu ^{\textrm{Y};\textrm{K}}(t)\big )_{t \ge 0}$ is a Markov process with infinitesimal generator of the form

60 $$\begin{aligned} \mathcal {L}^\textrm{K}F_{(f^\textrm{X},f^\textrm{Y})}(\nu ) = \mathcal {L}_d^\textrm{K}F_{(f^\textrm{X},f^\textrm{Y})}(\nu ) + \sum _{h \in \{\textrm{r},\textrm{a},\textrm{b},\dot{\textrm{d}}\}} \mathcal {L}_{h}^\textrm{K}F_{(f^\textrm{X},f^\textrm{Y})}(\nu )\,, \end{aligned}$$

with

61 $$\begin{aligned} \begin{aligned} \mathcal {L}_d^\textrm{K}F_{(f^\textrm{X},f^\textrm{Y})}(\nu )&= \left\langle \mathcal {L}_d^\textrm{X}f^\textrm{X},\nu ^\textrm{X} \right\rangle \frac{\partial }{\partial x} F \left( \left\langle f^\textrm{X},\nu ^XX \right\rangle , \left\langle f^\textrm{Y},\nu ^XX \right\rangle \right) \\&\quad + \left\langle \mathcal {L}_d^\textrm{Y}f^\textrm{Y},\nu ^\textrm{Y} \right\rangle \frac{\partial }{\partial y} F \left( \left\langle f^\textrm{X},\nu \right\rangle , \left\langle f^\textrm{Y},\nu \right\rangle \right) \\&\quad + \left\langle \nabla ^T f^\textrm{X}\, \sigma ^\textrm{X}\,\nabla f^\textrm{X},\nu ^\textrm{X} \right\rangle \frac{1}{\textrm{K}}\frac{\partial ^2}{\partial x^2} F \left( \left\langle f^\textrm{X},\nu \right\rangle , \left\langle f^\textrm{Y},\nu \right\rangle \right) \\&\quad + \left\langle \nabla ^T f^\textrm{Y}\, \sigma ^\textrm{Y}\,\nabla f^\textrm{Y},\nu ^\textrm{Y} \right\rangle \frac{1}{\textrm{K}} \frac{\partial ^2}{\partial y^2} F \left( \left\langle f^\textrm{X},\nu \right\rangle , \left\langle f^\textrm{Y},\nu \right\rangle \right) \,, \end{aligned} \end{aligned}$$

and

62 $$\begin{aligned} \begin{aligned}&\mathcal {L}_{\textrm{r}}^\textrm{K}F_{(f^\textrm{X},f^\textrm{Y})}({\varvec{\nu }})\\&\quad = \textrm{K}\int _{\textrm{Q}} \textrm{r}(q,\nu )\left[ F_{(f^\textrm{X},f^\textrm{Y})}\left( \nu ^\textrm{X}- \frac{1}{\textrm{K}}\delta _{q},\nu ^\textrm{Y}\right) - F_{(f^\textrm{X},f^\textrm{Y})}(\nu ) \right] \nu ^\textrm{X}(dq)\,,\\&\mathcal {L}_{\textrm{a}}^\textrm{K}F_{(f^\textrm{X},f^\textrm{Y})}({\varvec{\nu }})\\&\quad = \textrm{K}\int _{\textrm{Q}} \int _{\textrm{Q}} \textrm{a}(q,\nu )\left[ F_{(f^\textrm{X},f^\textrm{Y})}\left( \nu ^\textrm{X}- \frac{1}{\textrm{K}} \delta _{q},\nu ^\textrm{Y}+ \frac{1}{\textrm{K}} \delta _{\bar{q}}\right) - F_{(f^\textrm{X},f^\textrm{Y})}(\nu ) \right] m^{\textrm{a}}(\bar{q}|q)d\bar{q}\nu ^\textrm{X}(dq)\,,\\&\mathcal {L}_{\textrm{b}}^\textrm{K}F_{(f^\textrm{X},f^\textrm{Y})}({\varvec{\nu }})\\&\quad = \textrm{K}^2 \int _{\tilde{\textrm{Q}}^2} \int _{\textrm{Q}} \textrm{p}(q_1,q_2)\textrm{b}(q_1,q_2,\nu )\left[ F_{(f^\textrm{X},f^\textrm{Y})}\left( \nu ^\textrm{X}- \frac{1}{\textrm{K}} \delta _{q_1} - \frac{1}{\textrm{K}} \delta _{q_2},\nu ^\textrm{Y}+ \frac{1}{\textrm{K}} \delta _{\bar{q}}\right) - F_{(f^\textrm{X},f^\textrm{Y})}(\nu ) \right] \\&\qquad \times m^{\textrm{b}}(\bar{q}|q_1,q_2)d\bar{q}\nu ^\textrm{X}(dq_1)\nu ^\textrm{X}(dq_2) \\&\qquad +\int _{\tilde{\textrm{Q}}^2} \left( 1-\textrm{p}(q_1,q_2)\right) \textrm{b}(q_1,q_2,\nu )\left[ F_{(f^\textrm{X},f^\textrm{Y})}\left( \nu ^\textrm{X}-\frac{1}{\textrm{K}} \delta _{q_1} -\frac{1}{\textrm{K}} \delta _{q_2},\nu ^\textrm{Y}\right) - F_{(f^\textrm{X},f^\textrm{Y})}(\nu ) \right] \nu ^\textrm{X}(dq_1)\nu ^\textrm{X}(dq_2)\,,\\&\mathcal {L}_{\dot{\textrm{d}}}^\textrm{K}F_{(f^\textrm{X},f^\textrm{Y})}({\varvec{\nu }})\\&\quad = \dot{\textrm{d}}\int _{\mathbb {N}^2} \int _{\textrm{Q}^{|(\xi ^\textrm{X},\xi ^\textrm{Y})|}} \left[ F_{(f^\textrm{X},f^\textrm{Y})}\left( \nu ^\textrm{X}+\frac{1}{\textrm{K}}\sum _{i=1}^{\xi ^\textrm{X}}\delta _{q_i^\textrm{X}},\nu ^\textrm{Y}+\frac{1}{\textrm{K}}\sum _{i=1}^{\xi ^\textrm{Y}}\delta _{q_i^\textrm{Y}}\right) - F_{(f^\textrm{X},f^\textrm{Y})}(\nu ) \right] \\&\qquad \times \zeta _\xi (\left. q^\textrm{X},q^\textrm{Y}\right| \xi ^\textrm{X},\xi ^\textrm{Y}) dp(\xi ^\textrm{X},\xi ^\textrm{Y}) dq^\textrm{X}\,dq^\textrm{Y}\,.\\ \end{aligned} \end{aligned}$$

We thus can infer from Theorem 4.4 the following martingale property for the rescaled system.

### Lemma 5.2

Consider $\textrm{K}\ge 1$ fixed, assume that Hypothesis 3.4–4.2 holds true and that $\nu ^\textrm{X}_0$ and $\nu ^\textrm{Y}_0$ are two random measures independent with finite $p-$th moment, $p \ge 2$. Then, the processes $\textrm{M}^\textrm{X}$ and $\textrm{M}^\textrm{Y}$ defined for $f^\textrm{X}\,,\,f^\textrm{Y}\in C^2_0$ by

63 $$\begin{aligned} {\left\{ \begin{array}{ll} \textrm{M}^\textrm{X}(t) &{}= \left\langle f^\textrm{X},u^{\textrm{X};\textrm{K}}(t) \right\rangle - \left\langle f^\textrm{X},u^{\textrm{X};\textrm{K}}_0 \right\rangle - \int _0^t \left\langle \mathcal {L}^\textrm{X}_d f^\textrm{X}(x),u^{\textrm{X};\textrm{K}}(s) \right\rangle ds \\ &{}+ \int _0^t\int _{\textrm{Q}} \left[ \textrm{r}^\textrm{K}(q,\nu ) + \textrm{a}^\textrm{K}(q,\nu )\right] f^\textrm{X}(q)u^{\textrm{X};\textrm{K}}(s)(dq)ds\\ &{}+\int _0^t\int _{\tilde{\textrm{Q}}^2}\textrm{b}^\textrm{K}(q_1,q_2,\nu ) (f^\textrm{X}(q_1)+f^\textrm{X}(q_2))u^{\textrm{X};\textrm{K}}(s)(dq_1)u^{\textrm{X};\textrm{K}}(s)(dq_2)ds\\ &{}-\int _0^t \int _{\mathbb {N}} \int _{\tilde{\textrm{Q}}^{\xi ^\textrm{X}}} \dot{\textrm{d}}^\textrm{K}\left( \sum _{i=1}^{\xi ^\textrm{X}}f^\textrm{X}(q_i^\textrm{X}) \right) \zeta _{\xi ^\textrm{X}}(\left. q^\textrm{X}\right| \xi ^\textrm{X}) dq^\textrm{X}dp_{\textrm{X}}(\xi ^\textrm{X}) ds\,,\\ \textrm{M}^\textrm{Y}(t) &{}= \left\langle f^\textrm{Y},u^{\textrm{Y};\textrm{K}}(t) \right\rangle - \left\langle f^\textrm{Y},u^{\textrm{Y};\textrm{K}} \right\rangle - \int _0^t \left\langle \mathcal {L}^\textrm{Y}_d f^\textrm{Y}(x),u^{\textrm{Y};\textrm{K}}(s) \right\rangle ds \\ &{}- \int _0^t \int _{\textrm{Q}} \textrm{a}^\textrm{K}(q,\nu ) \int _{\textrm{Q}} m^{\textrm{a}}(\bar{q}|q) f^\textrm{Y}(\bar{q})d\bar{q} u^{\textrm{X};\textrm{K}}(s)(dq)ds\\ &{}- \int _0^t\int _{\tilde{\textrm{Q}}^2} \int _{\textrm{Q}} \textrm{p}(q_1,q_2) \textrm{b}^\textrm{K}(q_1,q_2,\nu ) f^\textrm{Y}(\bar{q}) m^{\textrm{b}}(\bar{q}|q_1,q_2)d\bar{q}u^{\textrm{X};\textrm{K}}(s)(dq_1)u^{\textrm{X};\textrm{K}}(s)(dq_2)ds\\ &{}-\int _0^t \int _{\mathbb {N}} \int _{\tilde{\textrm{Q}}^{\xi ^\textrm{Y}}} \dot{\textrm{d}}^\textrm{K}\left( \sum _{i=1}^{\xi ^\textrm{Y}} f^\textrm{Y}(q_i^\textrm{Y}) \right) \zeta _{\xi ^\textrm{Y}}(\left. q^\textrm{Y}\right| \xi ^\textrm{Y})dq^\textrm{Y}dp_{\textrm{Y}}(\xi ^\textrm{Y}) ds\,, \end{array}\right. } \end{aligned}$$

are cádlág $L^2-$martingales starting at 0 with predictable quadratic variation given by

64 $$\begin{aligned} {\left\{ \begin{array}{ll} \left\langle \textrm{M}^\textrm{X} \right\rangle (t) &{}= \frac{1}{\textrm{K}}\int _0^t \left\langle \nabla ^T f^\textrm{X}\sigma ^\textrm{X}\nabla f^\textrm{X}, u^{\textrm{X};\textrm{K}} \right\rangle ds \\ &{}+ \frac{1}{\textrm{K}}\int _0^t \int _{\textrm{Q}} \left[ \textrm{r}^\textrm{K}(q,\nu ) + \textrm{a}^\textrm{K}(q,\nu ) \int _{\textrm{Q}} m^{\textrm{a}}(\bar{q}|q)d\bar{q} \right] \left( f^\textrm{X}(q) \right) ^2 u^{\textrm{X};\textrm{K}}(s)(dq)ds\\ &{} +\frac{1}{\textrm{K}}\int _0^t\int _{\tilde{\textrm{Q}}^2} \int _{\textrm{Q}} \textrm{p}(q_1,q_2)\textrm{b}^\textrm{K}(q_1,q_2,\nu ) (f^\textrm{X}(q_1)+f^\textrm{X}(q_2))^2 m^{\textrm{b}}(\bar{q}|q_1,q_2)d\bar{q}u^{\textrm{X};\textrm{K}}(s)(dq_1)u^{\textrm{X};\textrm{K}}(s)(dq_2)ds\\ &{} +\frac{1}{\textrm{K}}\int _0^t\int _{\tilde{\textrm{Q}}^2} \left( 1-\textrm{p}(q_1,q_2)\right) \textrm{b}^\textrm{K}(q_1,q_2,\nu ) (f^\textrm{X}(q_1)+f^\textrm{X}(q_2))^2 u^{\textrm{X};\textrm{K}}(s)(dq_1)u^{\textrm{X};\textrm{K}}(s)(dq_2)ds\\ &{}-\frac{1}{\textrm{K}}\int _0^t \int _{\mathbb {N}} \int _{\tilde{\textrm{Q}}^{\xi ^\textrm{X}}} \dot{\textrm{d}}^\textrm{K}\left( \sum _{i=1}^{\xi ^\textrm{X}}f^\textrm{X}(q_i^\textrm{X}) \right) ^2 \zeta _{\xi ^\textrm{X}}(\left. q^\textrm{X}\right| \xi ^\textrm{X}) dq^\textrm{X}dp_{\textrm{X}}(\xi ^\textrm{X}) ds\,,\\ \left\langle \textrm{M}^\textrm{Y} \right\rangle (t) &{}= \frac{1}{\textrm{K}}\int _0^t \left\langle \nabla ^T f^\textrm{Y}\sigma ^\textrm{Y}\nabla f^\textrm{Y}, u^{\textrm{Y};\textrm{K}} \right\rangle ds \\ &{}- \frac{1}{\textrm{K}}\int _0^t \int _{\textrm{Q}} \textrm{a}^\textrm{K}(q,\nu ) \int _{\textrm{Q}} m^{\textrm{a}}(\bar{q}|q) \left( f^\textrm{Y}(\bar{q})\right) ^2 d\bar{q} u^{\textrm{X};\textrm{K}}(s)(dq)ds\\ &{}-\frac{1}{\textrm{K}} \int _0^t\int _{\tilde{\textrm{Q}}^2} \int _{\textrm{Q}} \textrm{p}(q_1,q_2) \textrm{b}^\textrm{K}(q_1,q_2,\nu ) \left( f^\textrm{Y}(\bar{q})\right) ^2 m^{\textrm{b}}(\bar{q}|q_1,q_2)d\bar{q}u^{\textrm{X};\textrm{K}}(s)(dq_1)u^{\textrm{X};\textrm{K}}(s)(dq_2)ds\\ &{}-\frac{1}{\textrm{K}}\int _0^t \int _{\mathbb {N}} \int _{\tilde{\textrm{Q}}^{\xi ^\textrm{Y}}} \dot{\textrm{d}}^\textrm{K}\left( \sum _{i=1}^{\xi ^\textrm{Y}} f^\textrm{Y}(q_i^\textrm{Y}) \right) ^2 \zeta _{\xi ^\textrm{Y}}(\left. q^\textrm{Y}\right| \xi ^\textrm{Y})dq^\textrm{Y}dp_{\textrm{Y}}(\xi ^\textrm{Y}) ds\,. \end{array}\right. } \end{aligned}$$

### Proof

The proof is analogous to the proof of Theorem 3.10. $\square$

We assume the following.

### Hypothesis 5.3

6. rescaled system:
  (6.i) the initial measure $u_0^\textrm{K}= (u_0^{\textrm{X};\textrm{K}},u_0^{\textrm{Y};\textrm{K}})$ converges in law for the weak topology on $\mathcal {M}_{\textrm{F}}(\textrm{Q}) \times \mathcal {M}_{\textrm{F}}(\textrm{Q})$ to some deterministic finite measure $u_0 = (u_0^{\textrm{X}},u_0^{\textrm{Y}}) \in \mathcal {M}_{\textrm{F}}(\textrm{Q}) \times \mathcal {M}_{\textrm{F}}(\textrm{Q})$; also we assume $\sup _{\textrm{K}} \mathbb {E} \left\langle \textbf{1},u^{\textrm{K}}_0 \right\rangle ^3 < \infty$;
  (6.ii) all the parameters $\textrm{r}^\textrm{K}$, $\textrm{a}^\textrm{K}$ and $\textrm{b}^\textrm{K}$ are continuous on the corresponding space, that is either $\textrm{Q}\times \mathbb {R}$ or $\textrm{Q}\times \textrm{Q}\times \mathbb {R}$ and they are Lipschitz continuous w.r.t. the last variable, that is there exist positive constants $L_\textrm{r}$, $L_\textrm{a}$ and $L_\textrm{b}$ such that, for all $q\,,\,q_1\,,\,q_2 \in \textrm{Q}$ and $v_1\,,\,v_2\in \mathbb {R}$ it holds
    $$\begin{aligned} \begin{aligned}&\left| \textrm{r}(q,v_1)-\textrm{r}(q,v_2)\right| \le L_\textrm{r}\left| v_1-v_2 \right| \,,\\&\left| \textrm{a}(q,v_1)-\textrm{r}(q,v_2)\right| \le L_\textrm{a}\left| v_1-v_2 \right| \,,\\&\left| \textrm{b}(q_1,q_2,v_1)-\textrm{r}(q_1,q_2,v_2)\right| \le L_\textrm{b}\left| v_1-v_2 \right| \,.\\ \end{aligned} \end{aligned}$$

Next is the main result of the present section.

### Theorem 5.4

Assume that Hypothesis 3.4–4.2–5.3 holds and consider $u^\textrm{K}$ as defined in Eq. (58). Then for all $T>0$, the sequence $\left( u^\textrm{K}\right) _{\textrm{K}\in \mathbb {N}}$ converges in law in $\mathcal {D}\left( [0,T];\mathcal {M}_{\textrm{F}}(\textrm{Q})\right)$ to a deterministic continuous measure-valued function in $\mathcal {C}\left( [0,T];\mathcal {M}_{\textrm{F}}(\textrm{Q})\right)$, which is the unique weak solution of the following non-linear integro-differential equation, $f^\textrm{X}\,,\,f^\textrm{Y}\in C^2_0$,

65 $$\begin{aligned} {\left\{ \begin{array}{ll} \left\langle f^\textrm{X},u^\textrm{X}(t) \right\rangle &{}= \left\langle f^\textrm{X},u^\textrm{X}_0 \right\rangle + \int _0^t \left\langle \mathcal {L}^\textrm{X}_d f^\textrm{X}(x),u^\textrm{X}(s) \right\rangle ds \\ &{}- \int _0^t\int _{\textrm{Q}} \left[ \textrm{r}(q,u) + \textrm{a}(q,u)\right] f^\textrm{X}(q)u^\textrm{X}(s)(dq)ds\\ &{}-\int _0^t\int _{\tilde{\textrm{Q}}^2}\textrm{b}(q_1,q_2,u) (f^\textrm{X}(q_1)+f^\textrm{X}(q_2))u^\textrm{X}(s)(dq_1)u^\textrm{X}(s)(dq_2)ds\\ &{}+\int _0^t \int _{\mathbb {N}} \int _{\tilde{\textrm{Q}}^{\xi ^\textrm{X}}} \dot{\textrm{d}}\left( \sum _{i=1}^{\xi ^\textrm{X}}f^\textrm{X}(q_i^\textrm{X}) \right) \zeta _{\xi ^\textrm{X}}(\left. q^\textrm{X}\right| \xi ^\textrm{X}) dq^\textrm{X}dp_{\textrm{X}}(\xi ^\textrm{X}) ds\,,\\ \left\langle f^\textrm{Y},u^\textrm{Y}(t) \right\rangle &{}= \left\langle f^\textrm{Y},u^\textrm{Y} \right\rangle + \int _0^t \left\langle \mathcal {L}^\textrm{Y}_d f^\textrm{Y}(x),u^\textrm{Y}(s) \right\rangle ds \\ &{}+ \int _0^t \int _{\textrm{Q}} \textrm{a}(q,u) \int _{\textrm{Q}} m^{\textrm{a}}(\bar{q}|q) f^\textrm{Y}(\bar{q})d\bar{q} u^\textrm{X}(s)(dq)ds\\ &{}+ \int _0^t\int _{\tilde{\textrm{Q}}^2} \int _{\textrm{Q}} \textrm{p}(q_1,q_2) \textrm{b}(q_1,q_2,u) f^\textrm{Y}(\bar{q}) m^{\textrm{b}}(\bar{q}|q_1,q_2)d\bar{q}u^\textrm{X}(s)(dq_1)u^\textrm{X}(s)(dq_2)ds\\ &{}+\int _0^t \int _{\mathbb {N}} \int _{\tilde{\textrm{Q}}^{\xi ^\textrm{Y}}} \dot{\textrm{d}}\left( \sum _{i=1}^{\xi ^\textrm{Y}} f^\textrm{Y}(q_i^\textrm{Y}) \right) \zeta _{\xi ^\textrm{Y}}(\left. q^\textrm{Y}\right| \xi ^\textrm{Y})dq^\textrm{Y}dp_{\textrm{Y}}(\xi ^\textrm{Y}) ds\,. \end{array}\right. } \end{aligned}$$

Further, *u* satisfies

66 $$\begin{aligned} \sup _{t \in [0,T]} \left\langle \textbf{1},u(t) \right\rangle <\infty \,. \end{aligned}$$

The steps of the proof of Theorem 5.4 are standard in the literature (Popovic and Véber 2023; Bansaye and Méléard 2015), or also (Isaacson et al. 2022) for the case of a bimolecular reaction. However, it is worth noting that comprehensive results encompassing the case addressed in this paper are not yet fully accessible. For instance (Isaacson et al. 2022) cannot account for zeroth-order reactions. The most extensive findings in this context have been established in Popovic and Véber (2023), although it is important to mention that the random generation of lesions has not been incorporated. For this reason, we will in the next Sections prove the large-population limit for the spatial model developed in Sect. 4. As customary, for the sake of readability the proof of Theorem 5.4 will be divided into four steps.

### Step 1: uniqueness of solution

The first result concerns the proof of the uniqueness of the limiting process (65).

#### Theorem 5.5

There exists a unique solution to the Eq. (65) in $\mathcal {C}\left( [0,T];\mathcal {M}_{\textrm{F}}(\textrm{Q})\right)$.

#### Proof

Arguments similar to the proof in Theorem 4.3 imply that neglecting negative terms and using Grownall’s lemma, the following estimate holds

$$\begin{aligned} \sup _{t \in [0,T]} \left\langle \textbf{1},u^\textrm{X}(t) \right\rangle \le C\,,\quad \sup _{t \in [0,T]} \left\langle \textbf{1},u^\textrm{Y}(t) \right\rangle \le C\,. \end{aligned}$$

Consider then two different solutions $u_1=(u^\textrm{X}_1,u^\textrm{Y}_1)$ and $u_2=(u^\textrm{X}_2,u^\textrm{Y}_2)$ to the Eq. (65), satisfying

$$\begin{aligned} \sup _{t \in [0,T]} \left\langle \textbf{1},u^\textrm{X}_1(t)-u^\textrm{X}_2(t) \right\rangle< C(T)< \infty \,. \end{aligned}$$

‘ $T^\textrm{X}$ and $T^\textrm{Y}$ the semigroups generated by the operators $\mathcal {L}^\textrm{X}_d$ and $\mathcal {L}^\textrm{Y}_d$. We thus have for any bounded and measurable functions *f* such that $\Vert f\Vert _\infty \le 1$,

67 $$\begin{aligned} {\left\{ \begin{array}{ll} \left\langle f^\textrm{X},u^\textrm{X}(t) \right\rangle &{}= \left\langle T^\textrm{X}(t)f^\textrm{X},u^\textrm{X}_0 \right\rangle \\ &{}\quad - \int _0^t\int _{\textrm{Q}} \left[ \textrm{r}(q,u) + \textrm{a}(q,u) \int _{\textrm{Q}} \right] T^\textrm{X}(t-s)f^\textrm{X}(q)u^\textrm{X}(s)(dq)ds\\ &{}\quad -\int _0^t\int _{\tilde{\textrm{Q}}^2}\textrm{b}(q_1,q_2,u) T^\textrm{X}(t-s)(f^\textrm{X}(q_1)+f^\textrm{X}(q_2))u^\textrm{X}(s)(dq_1)u^\textrm{X}(s)(dq_2)ds\\ &{}\quad +\int _0^t \int _{\mathbb {N}} \int _{\tilde{\textrm{Q}}^{\xi ^\textrm{X}}} \dot{\textrm{d}}\left( \sum _{i=1}^{\xi ^\textrm{X}}f^\textrm{X}(q_i^\textrm{X}) \right) \zeta _{\xi ^\textrm{X}}(\left. q^\textrm{X}\right| \xi ^\textrm{X}) dq^\textrm{X}dp_{\textrm{X}}(\xi ^\textrm{X}) ds\,,\\ \left\langle f^\textrm{Y},u^\textrm{Y}(t) \right\rangle &{}= \left\langle T^\textrm{Y}(t)f^\textrm{Y},u^\textrm{Y} \right\rangle \\ &{}\quad + \int _0^t \int _{\textrm{Q}} \textrm{a}(q,u) \int _{\textrm{Q}} m^{\textrm{a}}(\bar{q}|q) T^\textrm{Y}(t-s)f^\textrm{Y}(\bar{q})d\bar{q} u^\textrm{X}(s)(dq)ds\\ &{}\quad + \int _0^t\int _{\tilde{\textrm{Q}}^2} \int _{\textrm{Q}} \textrm{p}(q_1,q_2) \textrm{b}(q_1,q_2,u) T^\textrm{Y}(t-s)f^\textrm{Y}(\bar{q}) m^{\textrm{b}}(\bar{q}|q_1,q_2)d\bar{q}u^\textrm{X}(s)(dq_1)u^\textrm{X}(s)(dq_2)ds\\ &{}\quad +\int _0^t \int _{\mathbb {N}} \int _{\tilde{\textrm{Q}}^{\xi ^\textrm{Y}}} \dot{\textrm{d}}\left( \sum _{i=1}^{\xi ^\textrm{Y}} f^\textrm{Y}(q_i^\textrm{Y}) \right) \zeta _{\xi ^\textrm{Y}}(\left. q^\textrm{Y}\right| \xi ^\textrm{Y})dq^\textrm{Y}dp_{\textrm{Y}}(\xi ^\textrm{Y}) ds\,. \end{array}\right. } \end{aligned}$$

We thus have

68 $$\begin{aligned} {\left\{ \begin{array}{ll} &{}\left| \left\langle f^\textrm{X},u^\textrm{X}_1(t)-u^\textrm{X}_2(t) \right\rangle \right| \le \int _0^t\left| \int _{\textrm{Q}} \left[ \textrm{r}(q,u_1) + \textrm{a}(q,u_1) \right] T^\textrm{X}(t-s)f^\textrm{X}(q)\left( u_1^\textrm{X}(s)(dq)-u_2^\textrm{X}(s)(dq)\right) \right| ds\\ &{}\quad +\int _0^t\left| \int _{\tilde{\textrm{Q}}^2} \textrm{b}(q_1,q_2,u_1) T^\textrm{X}(t-s)(f^\textrm{X}(q_1)+f^\textrm{X}(q_2))\left( u_1^\textrm{X}(s)(dq_1)u_1^\textrm{X}(s)(dq_q)-u_2^\textrm{X}(s)(dq_1)u_2^\textrm{X}(s)(dq_1)\right) \right| ds\\ &{}\quad +\int _0^t\left| \int _{\mathbb {N}} \int _{\tilde{\textrm{Q}}^{\xi ^\textrm{X}}} \dot{\textrm{d}}\left( \sum _{i=1}^{\xi ^\textrm{X}}T^\textrm{X}(t-s)f^\textrm{X}(q_i^\textrm{X}) \right) \zeta _{\xi ^\textrm{X}}(\left. q^\textrm{X}\right| \xi ^\textrm{X}) dp_{\textrm{X}}(\xi ^\textrm{X}) dq^\textrm{X}\right| ds \\ &{}\quad +\int _0^t\left| \int _{\textrm{Q}} \left[ \textrm{r}(q,u_2) + \textrm{a}(q,u_2) - \textrm{r}(q,u_1) - \textrm{a}(q,u_1)\int _{\textrm{Q}}\right] T^\textrm{X}(t-s)f^\textrm{X}(q)u_2^\textrm{X}(s)(dq)\right| ds\\ &{}\quad +\int _0^t\left| \int _{\tilde{\textrm{Q}}^2}\left( \textrm{b}(q_1,q_2,u_2)-\textrm{b}(q_1,q_2,u_1)\right) T^\textrm{X}(t-s)(f^\textrm{X}(q_1)+f^\textrm{X}(q_2))u_2^\textrm{X}(s)(dq_1)u_2^\textrm{X}(s)(dq_1)\right| ds\,. \end{array}\right. } \end{aligned}$$

Since it holds that $\Vert T^\textrm{X}(t-s)f^\textrm{X}(q)\Vert _\infty \le 1$, we have that

69 $$\begin{aligned} \begin{aligned}&\left| \left[ \textrm{r}(q,u_1) + \textrm{a}(q,u_1) \right] T^\textrm{X}(t-s)f^\textrm{X}(q)\right| \le \bar{\textrm{r}} + \bar{\textrm{a}}(1+|v|) \le C\,,\\&\left| \textrm{b}(q_1,q_2,u_1) T^\textrm{X}(t-s)(f^\textrm{X}(q_1)+f^\textrm{X}(q_2))\right| \le \bar{\textrm{b}}(1+|v|) \le C\,,\\&\left| \dot{\textrm{d}}\left( \sum _{i=1}^{\xi ^\textrm{X}}T^\textrm{X}(t-s)f^\textrm{X}(q_i^\textrm{X})\right) \right| \le C\,,\\&\left| \left[ \textrm{r}(q,u_2) + \textrm{a}(q,u_2) - \textrm{r}(q,u_1) - \textrm{a}(q,u_1)\right] T^\textrm{X}(t-s)f^\textrm{X}(q) \right| \\&\quad \le C \sup _{\Vert f^\textrm{X}\Vert _\infty \le 1} \left\langle \textbf{1},u_1(t)-u_2(t) \right\rangle \,,\\&\left| \left( \textrm{b}(q_1,q_2,u_2)-\textrm{b}(q_1,q_2,u_1)\right) T^\textrm{X}(t-s)(f^\textrm{X}(q_1)+f^\textrm{X}(q_2)) \right| \\&\quad \le C \sup _{\Vert f^\textrm{X}\Vert _\infty \le 1} \left\langle \textbf{1},u_1(t)-u_2(t) \right\rangle \,. \end{aligned} \end{aligned}$$

We therefore can infer, using estimates (69) in Eq. (68), that

$$\begin{aligned} \left| \left\langle f^\textrm{X},u^\textrm{X}_1(t)-u^\textrm{X}_2(t) \right\rangle \right| \le C \int _0^t \sup _{\Vert f^\textrm{X}\Vert _\infty \le 1} \left\langle f^\textrm{X},u_1(s)-u_2(s) \right\rangle ds\,. \end{aligned}$$

Using thus Gronwall’s lemma, we can finally conclude that for all $t\le T$,

$$\begin{aligned} \sup _{\Vert f^\textrm{X}\Vert _\infty \le 1} \left\langle f^\textrm{X},u_1(s)-u_2(s) \right\rangle =0\,, \end{aligned}$$

from which the uniqueness follows. $\square$

### Step 2: propagation of moments

#### Lemma 5.6

Assume that Hypothesis 3.4–4.2–5.3 holds, then for any $T>0$, there exists a constant $C:= C(T)>0$ which depends on *T* such that the following estimates hold

70 $$\begin{aligned} \sup _{\textrm{K}} \mathbb {E}\sup _{t \in [0,T]} \left\langle \textbf{1},u^{\textrm{X},\textrm{K}}(t) \right\rangle ^3 \le C \,,\quad \sup _{\textrm{K}} \mathbb {E}\sup _{t \in [0,T]} \left\langle \textbf{1},u^{\textrm{Y},\textrm{K}}(t) \right\rangle ^3 \le C \,. \end{aligned}$$

In particular, we further have

71 $$\begin{aligned} \sup _{\textrm{K}} \mathbb {E}\sup _{t \in [0,T]} \left\langle f^\textrm{X},u^{\textrm{X},\textrm{K}}(t) \right\rangle ^3 \le C \,,\quad \sup _{\textrm{K}} \mathbb {E}\sup _{t \in [0,T]} \left\langle f^\textrm{Y},u^{\textrm{Y},\textrm{K}}(t) \right\rangle ^3 \le C \,. \end{aligned}$$

#### Proof

The proof follows from computations similar to what is done in the proof of Theorem 4.3 obtaining a similar estimate as in Eq. (29). That is we have,

$$\begin{aligned} \mathbb {E}\sup _{t \in [0,T \wedge \tau ^\textrm{X}_n]} \left\langle \textbf{1},u^{\textrm{X},\textrm{K}}(t) \right\rangle ^3 \le C\,,\quad \mathbb {E}\sup _{t \in [0,T \wedge \tau ^\textrm{Y}_n]} \left\langle \textbf{1},u^{\textrm{Y},\textrm{K}}(t) \right\rangle ^3 \le C\,. \end{aligned}$$

Taking the limit $\tau ^\textrm{X}_n$ and $\tau ^\textrm{Y}_n$ as $\textrm{K}\rightarrow \infty$, Fatou’s lemma implies Eq. (70). Estimate (71) thus follows from the fact that $f^\textrm{X}$ and $f^\textrm{Y}$ are bounded. $\square$

### Step 3: tightness

In the following, we will denote by $\Lambda ^\textrm{K}$ the law of the process $u^\textrm{K}= (u^{\textrm{X};\textrm{K}},u^{\textrm{Y},\textrm{K}})$. We then have the following.

#### Theorem 5.7

Assume Hypothesis 3.4–4.2–5.3 hold, then the sequence of laws $\left( \Lambda ^\textrm{K}\right) _{\textrm{K}\in \mathbb {N}}$ on $\mathcal {D}\left( [0,T];\mathcal {M}_{\textrm{F}}\right)$ is tight when endowed with the vague topology.

#### Proof

We equip $\mathcal {M}_{\textrm{F}}$ with the vague topology; to prove the tightness of the sequence of laws $\Lambda ^\textrm{K}$, using Roelly-Coppoletta (1986), it is enough to show that the sequence of laws of the processes $\left\langle f^\textrm{X},u^{\textrm{X};\textrm{K}} \right\rangle$ and $\left\langle f^\textrm{Y},u^{\textrm{Y};\textrm{K}} \right\rangle$ are tight in $\mathcal {D}\left( [0,T];\mathbb {R}\right)$ for any function $f^\textrm{X}$ and $f^\textrm{Y}$. As standard, in order to accomplish this, we employ the Aldous criterion (Aldous 1978) and Rebolledo criterion (Joffe and Métivier 1986).

Notice first that, using the fact that $f^\textrm{X}$ and $f^\textrm{Y}$ are bounded, we have already proved estimates (71). Consider thus $\delta >0$ and two stopping times $(\tau _1,\tau _2)$ satisfying a.s. $0 \le \tau _1 \le \tau _2 \le \tau _2+\delta \le T$. Using Doob’s inequality, together with estimate (70) and the martingale representation given in Lemma 5.2,

$$\begin{aligned} \begin{aligned} \mathbb {E}\left[ \left\langle \textrm{M}^\textrm{X} \right\rangle (\tau _2) - \left\langle \textrm{M}^\textrm{X} \right\rangle (\tau _1)\right]&\le C \delta \mathbb {E}\left[ 1+\sup _{t \in [0,T]} \left\langle \textbf{1},u^{\textrm{X};\textrm{K}} \right\rangle ^3+\sup _{t \in [0,T]} \left\langle \textbf{1},u^{\textrm{Y};\textrm{K}} \right\rangle ^3 \right] \,,\\ \mathbb {E}\left[ \left\langle \textrm{M}^\textrm{Y} \right\rangle (\tau _2) - \left\langle \textrm{M}^\textrm{Y} \right\rangle (\tau _1)\right]&\le C \delta \mathbb {E}\left[ 1+\sup _{t \in [0,T]} \left\langle \textbf{1},u^{\textrm{X};\textrm{K}} \right\rangle ^3+\sup _{t \in [0,T]} \left\langle \textbf{1},u^{\textrm{Y};\textrm{K}} \right\rangle ^3 \right] \,. \end{aligned} \end{aligned}$$

Similarly, denoting by

72 $$\begin{aligned} {\left\{ \begin{array}{ll} A^\textrm{X}(t) &{}:= \int _0^t \left\langle \mathcal {L}^\textrm{X}_d f^\textrm{X}(x),u^{\textrm{X};\textrm{K}}(s) \right\rangle ds \\ &{}\quad - \int _0^t\int _{\textrm{Q}} \left[ \textrm{r}^\textrm{K}(q,\nu (s)) + \textrm{a}^\textrm{K}(q,\nu ) \right] f^\textrm{X}(q)u^{\textrm{X};\textrm{K}}(s)(dq)ds\\ &{}\quad -\int _0^t\int _{\tilde{\textrm{Q}}^2} \textrm{b}^\textrm{K}(q_1,q_2,\nu ) (f^\textrm{X}(q_1)+f^\textrm{X}(q_2))u^{\textrm{X};\textrm{K}}(s)(dq_1)u^{\textrm{X};\textrm{K}}(s)(dq_2)ds\\ &{}\quad +\int _0^t \int _{\mathbb {N}} \int _{\tilde{\textrm{Q}}^{\xi ^\textrm{X}}} \dot{\textrm{d}}^\textrm{K}\left( \sum _{i=1}^{\xi ^\textrm{X}}f^\textrm{X}(q_i^\textrm{X}) \right) \zeta _{\xi ^\textrm{X}}(\left. q^\textrm{X}\right| \xi ^\textrm{X}) dq^\textrm{X}dp_{\textrm{X}}(\xi ^\textrm{X}) ds\,,\\ A^\textrm{Y}(t) &{}:= \int _0^t \left\langle \mathcal {L}^\textrm{Y}_d f^\textrm{Y}(x),u^{\textrm{Y};\textrm{K}}(s) \right\rangle ds \\ &{}\quad + \int _0^t \int _{\textrm{Q}} \textrm{a}^\textrm{K}(q,\nu ) \int _{\textrm{Q}} m^{\textrm{a}}(\bar{q}|q) f^\textrm{Y}(\bar{q})d\bar{q} u^{\textrm{X};\textrm{K}}(s)(dq)ds\\ &{}\quad + \int _0^t\int _{\tilde{\textrm{Q}}^2} \int _{\textrm{Q}} \textrm{p}(q_1,q_2) \textrm{b}^\textrm{K}(q_1,q_2,\nu ) f^\textrm{Y}(\bar{q}) m^{\textrm{b}}(\bar{q}|q_1,q_2)d\bar{q}u^{\textrm{X};\textrm{K}}(s)(dq_1)u^{\textrm{X};\textrm{K}}(s)(dq_2)ds\\ &{}\quad +\int _0^t \int _{\mathbb {N}} \int _{\tilde{\textrm{Q}}^{\xi ^\textrm{Y}}} \dot{\textrm{d}}^\textrm{K}\left( \sum _{i=1}^{\xi ^\textrm{Y}} f^\textrm{Y}(q_i^\textrm{Y}) \right) \zeta _{\xi ^\textrm{Y}}(\left. q^\textrm{Y}\right| \xi ^\textrm{Y}) d q^{\textrm{Y}} dp_{\textrm{Y}}(\xi ^\textrm{Y}) ds\,, \end{array}\right. } \end{aligned}$$

the finite variation part of $\left\langle f^\textrm{X},u^{\textrm{X};\textrm{K}}(\tau _2) \right\rangle - \left\langle f^\textrm{X},u^{\textrm{X};\textrm{K}}(\tau _1) \right\rangle$ and $\left\langle f^\textrm{Y},u^{\textrm{Y};\textrm{K}}(\tau _2) \right\rangle - \left\langle f^\textrm{Y},u^{\textrm{Y};\textrm{K}}(\tau _1) \right\rangle$ we have that

$$\begin{aligned} \begin{aligned} \mathbb {E}\left[ \left\langle A^\textrm{X} \right\rangle (\tau _2) - \left\langle A^\textrm{X} \right\rangle (\tau _1)\right]&\le C \delta \mathbb {E}\left[ 1+\sup _{t \in [0,T]} \left\langle \textbf{1},u^{\textrm{X};\textrm{K}} \right\rangle ^3+\sup _{t \in [0,T]} \left\langle \textbf{1},u^{\textrm{Y};\textrm{K}} \right\rangle ^3 \right] \,,\\ \mathbb {E}\left[ \left\langle A^\textrm{Y} \right\rangle (\tau _2) - \left\langle A^\textrm{Y} \right\rangle (\tau _1)\right]&\le C \delta \mathbb {E}\left[ 1+\sup _{t \in [0,T]} \left\langle \textbf{1},u^{\textrm{X};\textrm{K}} \right\rangle ^3+\sup _{t \in [0,T]} \left\langle \textbf{1},u^{\textrm{Y};\textrm{K}} \right\rangle ^3 \right] \,.\\ \end{aligned} \end{aligned}$$

The claim thus follows. $\square$

### Step 4: identification of the limit

#### Theorem 5.8

Assume Hypothesis 3.4–4.2–5.3 hold, denote by $\Lambda$ the limiting law of the sequence of laws $\left( \Lambda ^\textrm{K}\right) _{\textrm{K}\in \mathbb {N}}$. Denote by *u* the process with law $\Lambda$. Then *u* is a.s. strongly continuous and it solves Eq. (65).

#### Proof

The fact that *u* is a.s. strongly continuous follows from the fact that

$$\begin{aligned} \sup _{t \in [0,T]}\sup _{\Vert f^\textrm{X}\Vert _{\infty } \le 1}\left| \left\langle f^\textrm{X},u^{\textrm{X};\textrm{K}}(t) \right\rangle - \left\langle f^\textrm{X},u^{\textrm{X};\textrm{K}}(t_-) \right\rangle \right| \le \frac{1}{\textrm{K}}\,. \end{aligned}$$

Consider $t\le T$, $f^\textrm{X}$, $f^\textrm{Y}$ and $\textrm{u} = (\textrm{u}^\textrm{X},\textrm{u}^\textrm{Y}) \in \mathcal {D}\left( [0,T],\mathcal {M}_{\textrm{F}}\times \mathcal {M}_{\textrm{F}}\right)$; define the functionals

$$\begin{aligned} \Psi ^\textrm{X}: \mathcal {D}\left( [0,T],\mathcal {M}_{\textrm{F}}\times \mathcal {M}_{\textrm{F}}\right) \rightarrow \mathbb {R}\,, \end{aligned}$$

as

$$\begin{aligned} \begin{aligned}&\Psi ^\textrm{X}_{t,f^\textrm{X}} (\textrm{u}^\textrm{X},\textrm{u}^\textrm{Y}) = \left\langle f^\textrm{X},u^\textrm{X}(t) \right\rangle - \left\langle f^\textrm{X},u^\textrm{X}_0 \right\rangle - \int _0^t \left\langle \mathcal {L}^\textrm{X}_d f^\textrm{X}(x),u^\textrm{X}(s) \right\rangle ds \\&\quad + \int _0^t\int _{\textrm{Q}} \left[ \textrm{r}(q,u) + \textrm{a}(q,u)\right] f^\textrm{X}(q)u^\textrm{X}(s)(dq)ds\\&\quad +\int _0^t\int _{\tilde{\textrm{Q}}^2}\textrm{b}(q_1,q_2,u) (f^\textrm{X}(q_1)+f^\textrm{X}(q_2))u^\textrm{X}(s)(dq_1)u^\textrm{X}(s)(dq_2)ds\\&\quad -\int _0^t \int _{\mathbb {N}} \int _{\tilde{\textrm{Q}}^{\xi ^\textrm{X}}} \dot{\textrm{d}}\left( \sum _{i=1}^{\xi ^\textrm{X}}f^\textrm{X}(q_i^\textrm{X}) \right) \zeta _{\xi ^{\textrm{X}}}(\left. q^\textrm{X}\right| \xi ^\textrm{X})dq^{\textrm{X}} dp_{\textrm{X}}(\xi ^\textrm{X}) ds\,,\\&\Psi ^\textrm{Y}_{t,f^\textrm{Y}} (\textrm{u}^\textrm{X},\textrm{u}^\textrm{Y}) = \left\langle f^\textrm{Y},u^\textrm{Y}(t) \right\rangle - \left\langle f^\textrm{Y},u^\textrm{Y} \right\rangle - \int _0^t \left\langle \mathcal {L}^\textrm{Y}_d f^\textrm{Y}(x),u^\textrm{Y}(s) \right\rangle ds \\&\quad - \int _0^t \int _{\textrm{Q}} \textrm{a}(q,u) \int _{\textrm{Q}} m^{\textrm{a}}(\bar{q}|q) f^\textrm{Y}(\bar{q})d\bar{q} u^\textrm{X}(s)(dq)ds\\&\quad - \int _0^t\int _{\tilde{\textrm{Q}}^2} \int _{\textrm{Q}} \textrm{p}(q_1,q_2) \textrm{b}(q_1,q_2,u) f^\textrm{Y}(\bar{q}) m^{\textrm{b}}(\bar{q}|q_1,q_2)d\bar{q}u^\textrm{X}(s)(dq_1)u^\textrm{X}(s)(dq_2)ds\\&\quad -\int _0^t \int _{\mathbb {N}^2} \int _{\tilde{\textrm{Q}}^{\xi ^\textrm{Y}}} \dot{\textrm{d}}\left( \sum _{i=1}^{\xi ^\textrm{Y}} f^\textrm{Y}(q_i^\textrm{Y}) \right) \zeta _{\xi ^{\textrm{Y}}}(\left. q^\textrm{Y}\right| \xi ^\textrm{Y}) dq^\textrm{Y}dp_{\textrm{Y}}(\xi ^\textrm{Y}) ds\,. \end{aligned} \end{aligned}$$

We aim to show that, for any $t \le T$,

$$\begin{aligned} \mathbb {E}\left| \Psi ^\textrm{X}_{t,f^\textrm{X}} (\textrm{u}^\textrm{X},\textrm{u}^\textrm{Y}) \right| = \mathbb {E}\left| \Psi ^\textrm{Y}_{t,f^\textrm{Y}} (\textrm{u}^\textrm{X},\textrm{u}^\textrm{Y}) \right| = 0\,. \end{aligned}$$

Using Lemma 5.2 we have that

$$\begin{aligned} \mathcal {M}^\textrm{X}(t) = \Psi ^\textrm{X}_{t,f^\textrm{X}} (\textrm{u}^{\textrm{X};\textrm{K}},\textrm{u}^{\textrm{Y};\textrm{K}})\,,\quad \mathcal {M}^\textrm{Y}(t) = \Psi ^\textrm{Y}_{t,f^\textrm{Y}} (\textrm{u}^{\textrm{X};\textrm{K}},\textrm{u}^{\textrm{Y};\textrm{K}})\,. \end{aligned}$$

Using thus Lemma 5.2 together with estimate (70) and Hypothesis 3.4–4.2–5.3, we have that

$$\begin{aligned} \begin{aligned}&\mathbb {E} \left| \mathcal {M}^{\textrm{X};\textrm{K}}(t) \right| ^2 = \mathbb {E} \left\langle \mathcal {M}^{\textrm{X};\textrm{K}} \right\rangle (t) \le \frac{C}{\textrm{K}}\,,\\&\mathbb {E} \left| \mathcal {M}^{\textrm{Y};\textrm{K}}(t) \right| ^2 = \mathbb {E} \left\langle \mathcal {M}^{\textrm{Y};\textrm{K}} \right\rangle (t) \le \frac{C}{\textrm{K}}\,,\\ \end{aligned} \end{aligned}$$

which goes to 0 as $\textrm{K}\rightarrow \infty$. Therefore, we have

$$\begin{aligned} \begin{aligned}&\lim _{\textrm{K}\rightarrow \infty }\mathbb {E} \left| \Psi ^\textrm{X}_{t,f^\textrm{X}} (\textrm{u}^{\textrm{X};\textrm{K}},\textrm{u}^{\textrm{Y};\textrm{K}})\right| = 0\,,\\&\lim _{\textrm{K}\rightarrow \infty }\mathbb {E} \left| \Psi ^\textrm{Y}_{t,f^\textrm{X}} (\textrm{u}^{\textrm{X};\textrm{K}},\textrm{u}^{\textrm{Y};\textrm{K}})\right| = 0\,. \end{aligned} \end{aligned}$$

Since $\textrm{u}$ is a.s. strongly continuous and the functions $f^\textrm{X}$ and $f^\textrm{Y}$ are bounded, then the functionals $\Psi ^\textrm{X}_{t,f^\textrm{X}}$ and $\Psi ^\textrm{Y}_{t,f^\textrm{Y}}$ are a.s. continuous at $\textrm{u}$. Also, for any $\mathcal {D}\left( [0,T],\mathcal {M}_{\textrm{F}}\times \mathcal {M}_{\textrm{F}}\right)$ we have

$$\begin{aligned} \begin{aligned}&\left| \Psi ^\textrm{X}_{t,f^\textrm{X}} (\textrm{u}^{\textrm{X};\textrm{K}},\textrm{u}^{\textrm{Y};\textrm{K}}) \right| \le C \sup _{s \in [0,T]}\left( 1 + \left\langle \textbf{1},u^\textrm{X}(s) \right\rangle ^2 + \left\langle \textbf{1},u^\textrm{Y}(s) \right\rangle ^2 \right) \,,\\&\left| \Psi ^\textrm{Y}_{t,f^\textrm{X}} (\textrm{u}^{\textrm{X};\textrm{K}},\textrm{u}^{\textrm{Y};\textrm{K}})\right| \le C \sup _{s \in [0,T]}\left( 1 + \left\langle \textbf{1},u^\textrm{X}(s) \right\rangle ^2 + \left\langle \textbf{1},u^\textrm{Y}(s) \right\rangle ^2 \right) \,. \end{aligned} \end{aligned}$$

Therefore the sequences $\left( \Psi ^\textrm{X}_{t,f^\textrm{X}} (\textrm{u}^{\textrm{X};\textrm{K}},\textrm{u}^{\textrm{Y};\textrm{K}})\right) _{\textrm{K}\in \mathbb {N}}$ and $\left( \Psi ^\textrm{Y}_{t,f^\textrm{Y}} (\textrm{u}^{\textrm{X};\textrm{K}},\textrm{u}^{\textrm{Y};\textrm{K}})\right) _{\textrm{K}\in \mathbb {N}}$ are uniformly integrable so that

$$\begin{aligned} \begin{aligned}&\lim _{\textrm{K}\rightarrow \infty }\mathbb {E} \left| \Psi ^\textrm{X}_{t,f^\textrm{X}} (\textrm{u}^{\textrm{X};\textrm{K}},\textrm{u}^{\textrm{Y};\textrm{K}})\right| = \mathbb {E} \left| \Psi ^\textrm{X}_{t,f^\textrm{X}} (\textrm{u}^{\textrm{X}},\textrm{u}^{\textrm{Y}})\right| \,,\\&\lim _{\textrm{K}\rightarrow \infty }\mathbb {E} \left| \Psi ^\textrm{Y}_{t,f^\textrm{Y}} (\textrm{u}^{\textrm{X};\textrm{K}},\textrm{u}^{\textrm{Y};\textrm{K}})\right| = \mathbb {E} \left| \Psi ^\textrm{Y}_{t,f^\textrm{Y}} (\textrm{u}^{\textrm{X}},\textrm{u}^{\textrm{Y}})\right| \,,\\ \end{aligned} \end{aligned}$$

which concludes the proof. $\square$

#### Theorem 5.9

Assume Hypothesis 3.4–4.2–5.3 hold, then the sequence of laws $\left( \Lambda ^\textrm{K}\right) _{\textrm{K}\in \mathbb {N}}$ on $\mathcal {D}\left( [0,T];\mathcal {M}_{\textrm{F}}\right)$ is tight when endowed with the weak topology.

#### Proof

Using Méléard and Roelly (1993), since the limiting process is continuous, repeating the above calculations with $f^\textrm{X}= f^\textrm{Y}= 1$, we obtain that the sequences $\left( \left\langle \textbf{1},u^{\textrm{X};\textrm{K}} \right\rangle \right) _{\textrm{K}}$ and $\left( \left\langle \textbf{1},u^{\textrm{Y};\textrm{K}} \right\rangle \right) _{\textrm{K}}$ converge to $\left\langle \textbf{1},u^{\textrm{X}} \right\rangle$ and to $\left\langle \textbf{1},u^{\textrm{Y}} \right\rangle$ in $\mathcal {D}\left( [0,T];\mathbb {R}\right)$. $\square$

### Step 5: proof of the convergence theorem

#### proof of Theorem 5.4

Putting together Theorems 5.5–5.8–5.9, the claim follows.

## Numerical results

The present Section is devoted to the implementation of the *spatial radiation-induced DNA damage repair model* defined above.

For the sake of simplicity, we will implement the model for a circular domain $\textrm{Q}\subset \mathbb {R}^2$ of radius 5 $\mu$m, the case of a more realistic scenario of a domain in $\mathbb {R}^3$ of arbitrary shape is straightforward. We assume that the radiation field is perpendicular to the cell nucleus, with a total absorbed dose of 10 Gy. We consider the radiation field generated by a monoenergetic beam of carbon ions at 40 Mev, with a fluence average specific energy $z_F=0.04$ Gy; a microdosimetric description of such radiation field has been considered for instance in Missiaggia et al. (2024). We simulate the number of impinging lesions according to a Poisson distribution of average $\frac{D}{z_F}$. The radiation field is assumed isotropic and uniform so that each tract is thus distributed uniformly on the nucleus. Then for each track, specific energy is sampled from the microdosimetric single-event distribution $f_1(z)$ and distributed spatially around the track according to the amorphous track model as described in Kase et al. (2007), which prescribes a radial dose distribution as

$$\begin{aligned} D(\rho ) = {\left\{ \begin{array}{ll} C_{\text{ c }} &{} \rho \le R_{\text{ c }}\,,\\ C_{\text{ p }}\frac{1}{\rho ^2} &{} R_{\text{ c }} < \rho \le R_{\text{ p }}\,,\\ 0 &{} \text{ otherwise }\,, \end{array}\right. } \end{aligned}$$

where $\rho$ is the distance from the center of the track, $R_{\text{ c }}$ is the core around the track characterized by a higher energy deposition outside which the dose decreases as $\rho ^{-2}$ until the radius of the penumbra $R_{\text{ p }}$ after which no energy deposition is registered. The constants considered are as defined in Kase et al. (2007) for the case of low-energy carbon ions considered. Thus, a random number of sub-lethal lesions, resp. lethal lesions are sampled around each track according to a Poisson random variable of average $\kappa z_i$, resp. $\lambda z_i$, with $\kappa = 50$ Gy$^{-1}$, resp. $\lambda = \kappa 10^{-2}$ Gy$^{-1}$.

Regarding the model, the following parameters have been chosen

73 $$\begin{aligned} \begin{aligned} \textrm{r}(q,v)&= \textrm{r}\left( 1 + \frac{1}{ \left\langle \mathbb {1}_{\left\{ |q-\bar{q}|< r_d\right\} } (\bar{q},\bar{s}),\nu \right\rangle +1}\right) \,,\\ \textrm{a}(q,v)&= \textrm{a}\left( 1 - \frac{1}{ \left\langle \mathbb {1}_{\left\{ |q-\bar{q}|< r_d\right\} } (\bar{q},\bar{s}),\nu \right\rangle +1}\right) \,,\\ \textrm{b}(q_1,q_2,v)&= \textrm{b}\mathbb {1}_{\left\{ |q_1 - q_2|<r_d\right\} }\,, \end{aligned} \end{aligned}$$

with $\textrm{r}= 4\, h^{-1}$ , $\textrm{a}= 0.1\, h^{-1}$ and $\textrm{b}=0.1\, h^{-1}$. Regarding these last constants, they agree with standard values calibrated in Missiaggia et al. (2024). Concerning instead Eq. (73), we assumed that the *repair rate* decreases, resp. the *death rate* increases, as the number of damages within a radius $r_d=0.5 \, \mu$m increases. We also assume a constant pairwise interaction only for the lesions within $r_d$ distance. It is worth stressing that such aspects are among the major strengths of the proposed model, where rates depend on the local concentration of damages. At last, we assume that in the case of pathway $\textrm{a}$, the new lethal lesion is created in the same position as the sub-lethal lesion was, whereas in the case of $\textrm{b}$ the new lethal lesion is created in the middle point between the two interacting sublethal lesions.

**Fig. 1:**
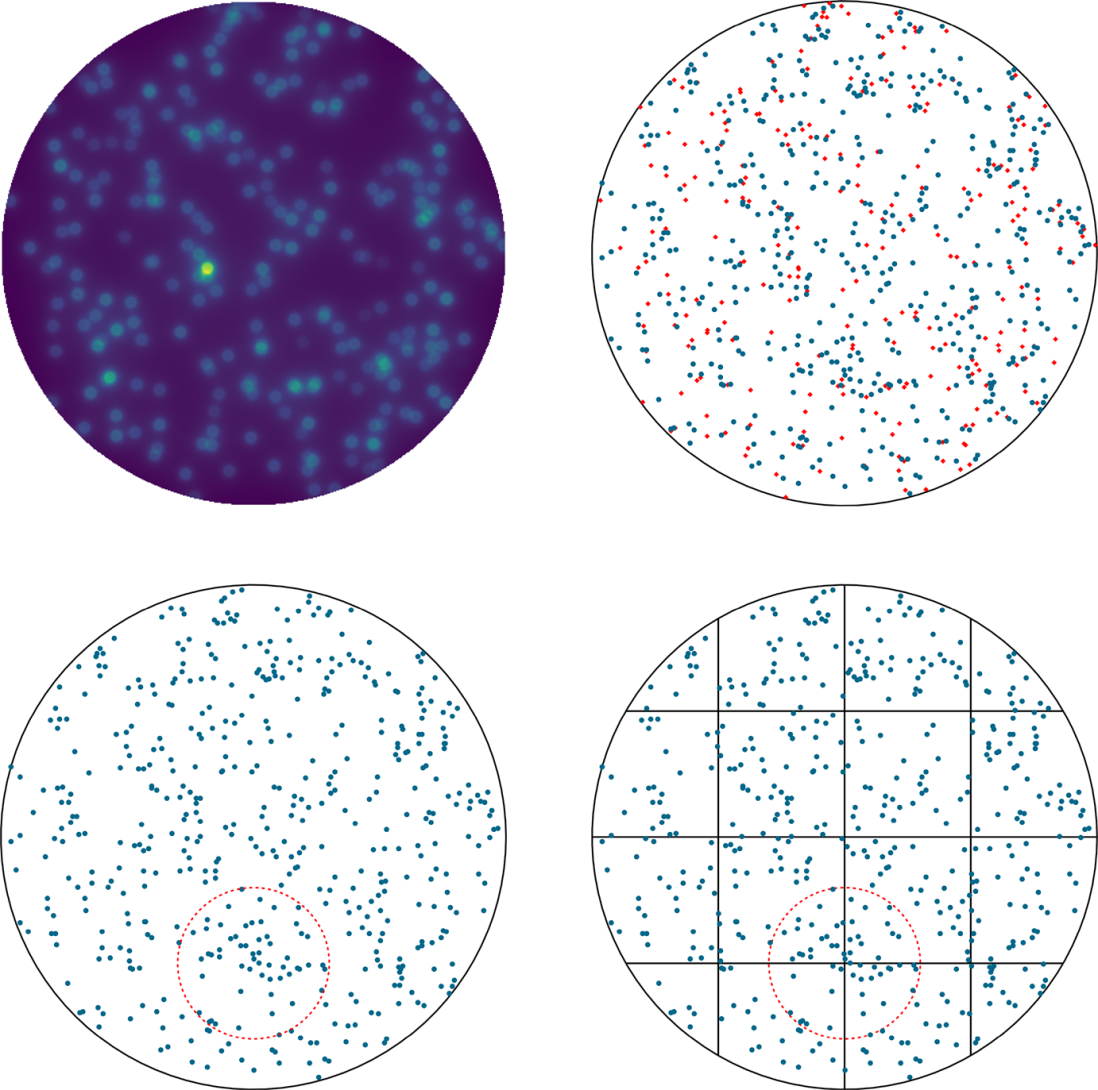
(top left panel) Dose deposited over the cell nucleus, light yellow regions represent high local dose depositions around the core of a track whereas dark purple regions represent low local dose deposition. (top right) Sub-lethal lesions (blue) and tracks hit (red). (bottom left) Sub-lethal lesions (blue) and a high local concentration of lesions within a 1.5 $\mu$m domain (red dashed circle). (bottom right) Discretization of sub-lethal lesions (blue) within fixed domains and a high local concentration of lesions across four different discrete domains (red dashed circle) (color figure online)

Figure 1 top left panel shows the normalized dose deposited over the cell nucleus, where a highly localized dose is deposited around the particle tracks, as depicted by lighter colors. In Fig. 1 top right panel the initial spatial distribution of sublethal lesions (in blue) and the position of track hit (in red) are depicted. It can be clearly seen how damages can be localized around tracks but some lesion clusters also emerge far from tracks. Figure 1 bottom panels show the initial spatial distribution of lesion in the left panel and a possibly discretized version in the right panel. A red circle highlights a dense region with a high number of lesions within a 1.5 $\mu$m radius. In a discrete domain formulation such cluster can be diluted between different domains, reducing the probability that these lesions interact via a pairwise interaction pathway leading to cell inactivation; on the contrary, in the continuous formulation no fixed domain is considered so that pairwise interaction is truly accounted based on the lesions spatial position. Since the pairwise interaction pathways is nonlinear, dividing the number of sublethal lesions in different domain ignoring their true spatial distribution can play a significant role in assessing the overall cell faith.

**Fig. 2:**
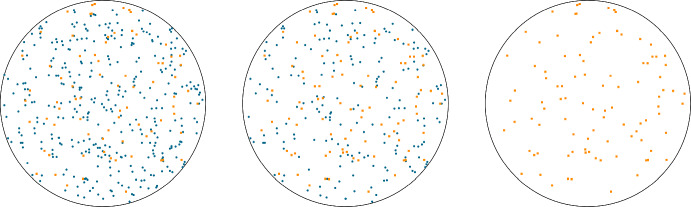
Time evolution, from left to right, of sub-lethal lesions (blue) and lethal lesion (orange) (color figure online)

Figure 2 shows a time evolution, from left to right, on the sublethal and lethal lesions: blue dots represent sublethal lesions whereas yellow dots represent lethal lesions. It is worth noticing how lethal lesions are effectively formed around the cluster highlighted in red in the bottom panels of Fig. 1, so that again considering the real distance between lesions can change the predicted cell faith and cell survival probability.

## Discussion and conclusions

In the present paper, we introduced a general stochastic model that describes the formation and repair of radiation-induced DNA damage. The derived model generalizes the previously studied model (Cordoni et al. 2021, 2022a, b), including a spatial description, allowing for pairwise interaction of cluster damages that might depend on the distances between damages, as well as a general description of the effect of radiation on biological tissue under a broad range of irradiation condition. We studied the mathematical well-posedness of the system and we characterized the large system behavior.

The implemented model represents an innovation in the field of theoretical radiobiology that can have a direct impact on radiotherapy. Indeed, from a theoretical point of view, various models to describe the formation and repair of radiation-induced DNA damage have been developed over the years, showing how various stochastic effects influence the two processes. However, the stochasticity of the spatial interaction of lesions close to each other, despite being recognized in the community to play a key role in the cell’s ability to repair the damage, had never been studied and modeled in a general and rigorous way. Therefore, the developed model is among the first mathematical models capable of providing a general and robust description of the processes of radiation-induced damage formation and repair. Furthermore, it was shown in the work how different time scales can be included in a single mathematical model, which then, in addition to the temporal and spatial stochasticities governing DNA damage repair, can also include different time scales thus providing an extremely general model.

A direct impact of the proposed model may occur in radiation therapy, where efforts have been made over the years to develop accurate models that could describe and predict the effect of radiation on biological tissue. The ultimate goal is to be able to implement optimal treatment plans that maximize the lethal effect of radiation on tumor tissue while minimizing undesirable effects on healthy tissue. This last aspect is indeed crucial: while the effect of the tumor is well studied, the undesirable and unavoidable effects on healthy tissue are much more difficult to predict. In fact, in areas far from the tumor, the radiation that is seen by the biological tissue is very heterogeneous, a fact that implies that the effects of such a varied radiation field are inherently stochastic. However, having a thorough knowledge of the effects of radiation in such areas is critical to increasing the long-term quality of life of patients undergoing radiation therapy. The model developed could have an impact on radiotherapy planning precisely because, given its extreme generality, it allows for knowledge and prediction of possible undesirable effects in areas more peripheral to the tumor. The possibility to also account for a continuous irradiation field could further improve the treatment modalities, since fractionation schemes, meaning that treatments are split and delivered over time, are nowadays commonly used in clinics to allow the healthy tissue to recover between consequent treatments.

Further, we believe that the derived model could play a role in describing an effect of recent interest in radiobiology, called the *FLASH effect*. In particular, at extremely high rates of particle delivery, it has been empirically seen that the unwanted effects of radiation on healthy tissue decrease while the killing effect on the tumor is maintained. Although numerous studies on the topic, the physical and biological mechanism at the very core of this effect is today largely unknown. It is nonetheless believed that spatial interactions of particles can play a major role in the origin of this effect. Therefore, the model introduced in the present research can have the generality to provide a stochastic description of the effect that spatial interdependence between particles can have on biological tissue.
